# Two New Bio-Inspired Particle Swarm Optimisation Algorithms for Single-Objective Continuous Variable Problems Based on Eavesdropping and Altruistic Animal Behaviours

**DOI:** 10.3390/biomimetics9090538

**Published:** 2024-09-05

**Authors:** Fevzi Tugrul Varna, Phil Husbands

**Affiliations:** AI Group, Department of Informatics, University of Sussex, Brighton BN1 9RH, UK

**Keywords:** bio-inspired search algorithm, optimisation, particle swarm optimisation, swarm intelligence, altruism, eavesdropping, group behaviour, metaheuristics

## Abstract

This paper presents two novel bio-inspired particle swarm optimisation (PSO) variants, namely biased eavesdropping PSO (BEPSO) and altruistic heterogeneous PSO (AHPSO). These algorithms are inspired by types of group behaviour found in nature that have not previously been exploited in search algorithms. The primary search behaviour of the BEPSO algorithm is inspired by eavesdropping behaviour observed in nature coupled with a cognitive bias mechanism that enables particles to make decisions on cooperation. The second algorithm, AHPSO, conceptualises particles in the swarm as energy-driven agents with bio-inspired altruistic behaviour, which allows for the formation of lending–borrowing relationships. The mechanisms underlying these algorithms provide new approaches to maintaining swarm diversity, which contributes to the prevention of premature convergence. The new algorithms were tested on the 30, 50 and 100-dimensional CEC’13, CEC’14 and CEC’17 test suites and various constrained real-world optimisation problems, as well as against 13 well-known PSO variants, the CEC competition winner, differential evolution algorithm L-SHADE and the recent bio-inspired I-CPA metaheuristic. The experimental results show that both the BEPSO and AHPSO algorithms provide very competitive performance on the unconstrained test suites and the constrained real-world problems. On the CEC13 test suite, across all dimensions, both BEPSO and AHPSO performed statistically significantly better than 10 of the 15 comparator algorithms, while none of the remaining 5 algorithms performed significantly better than either BEPSO or AHPSO. On the CEC17 test suite, on the 50D and 100D problems, both BEPSO and AHPSO performed statistically significantly better than 11 of the 15 comparator algorithms, while none of the remaining 4 algorithms performed significantly better than either BEPSO or AHPSO. On the constrained problem set, in terms of mean rank across 30 runs on all problems, BEPSO was first, and AHPSO was third.

## 1. Introduction

In recent years, swarm intelligence algorithms have become one of the most widely used class of optimisation methods [1,2,3]. Their effectiveness and convenience have led to many variants [4] and successful applications to diverse real-world problems [5,6,7,8]. One of the best known and most widely applied swarm algorithms is particle swarm optimisation (PSO) [9]. The PSO algorithm has been extensively investigated with regards to its search dynamics [10,11] and theoretical strengths and limitations [12,13], resulting in many recent extensions developed with the intention of improving the performance of the canonical PSO [14,15,16,17,18,19].

Despite the development of many variants with diverse inspirations, the core analogy at the heart of most PSO algorithms is biological—a simple model of flocking behaviour observed in many species. Indeed, bio-inspiration has become the dominant driving force for many new metaheuristic algorithms. However, the canonical PSO algorithm’s model of particle movement [9] is relatively simple compared to natural flocking behaviours. Hence, in most cases, the homogeneous nature of canonical PSO particles’ behaviour moves them towards a common goal using two standard ‘exemplars’ (or guiding positions), namely their ‘cognitive’ (own best position) and ‘social’ (swarm best position) influences, which tends to trigger rapid loss of diversity, leading to premature convergence. Various approaches have been proposed to address this problem over the past decade or so, including hybridisation with other search algorithms [16,20,21], using extended learning strategies [22,23,24] and employing more sophisticated topologies to define the local population structure [25,26,27,28]. Another powerful contemporary way to potentially minimise this issue and to improve the balance between exploitation and exploration within the search process is to design efficient, heterogeneous agent behaviours to avoid the stagnation of particles and improve overall performance by avoiding premature convergence [14,17,29].

In light of this, in the current paper, we propose two novel PSO algorithms that take their inspiration from forms of dynamic animal group behaviour that, as far as we know, have not previously been used in search algorithms, namely eavesdropping and altruism. These analogies are used to develop algorithms that possess heterogeneous behavioural dynamics at the both agent and swarm levels. Through this heterogeneity, more efficient exploration and exploitation search dynamics are enabled while maintaining diversity and avoiding premature convergence. Such effective exploration and exploitation performance is especially important for efficient searches of high-dimensional and complex problem spaces, which feeds into our overall motivation, i.e., the development of powerful new general-purpose optimisers for unconstrained and constrained single-objective, real-valued problems.

The first of these novel algorithms, BEPSO (biased eavesdropping PSO), is inspired by the alert-signalling behaviour of animals used to attract conspecifics (of the same species or group) to a discovered resource location for potential exploitation and the way in which surrounding heterospecific (of a different species or group) eavesdropper animals try to exploit that information themselves to improve their own fitness.

Eavesdropping plays a significant role in animal communication and the evolution of such communication [30]. Briefly, eavesdropping occurs as a result of animals accessing communication signals transmitted by heterospecifics that were not intended for them. In nature, it is more common for signal interceptors to be intraspecific (of the same species) in order to perceive the call and extract the required information accurately. However, it is not uncommon to observe interspecific animals (competitors of a different species) intercept signals and use them as an advantage to increase their own fitness. Interspecific eavesdropping is particularly interesting, as different species may be proficient in distinct areas within the same habitat and capable of recognising different threats through their distinct sensory capabilities. A concrete example of interspecies eavesdropping is illustrated by the relationship between red squirrels and Eurasian jays [31]. In this case, truly astonishing evolutionary dynamics have resulted in communication between a mammal and a bird, which have become positively biased towards one another and are able to warn and guard each other within the same habitat.

BEPSO takes ideas from animal eavesdropping to develop a set of dynamic interacting mechanisms that underlie particle behaviours and generate movement exemplars that prove to be far more efficient than the simple personal best position and population best positions used by the canonical PSO.

The second of the novel algorithms, altruistic heterogeneous PSO (AHPSO), is inspired by a certain kind of altruistic animal group behaviour.

The role of altruism in evolutionary dynamics was first analysed in mathematical detail by W.D. Hamilton in 1964 [32]. He showed how altruism could arise and be maintained within the Darwinian framework, conferring overall benefit to the group if not to the individual. Traditionally, researchers tended to assess the benefit of altruism to an organism by examining the average number of offspring, with contributors exhibiting less reproductive success in comparison to beneficiaries. However, several different types of altruistic behaviours have been discovered in various species. Some exemplary altruistic behaviours are observed in social insect colonies such as ants, wasps, bees and termites. In these colonies, the sterile workers are devoted to the queen by protecting the nest and foraging food. By doing so, sterile workers have no reproduction fitness, but they contribute to the queen’s reproductive efforts. An example of a more complex organism exhibiting altruistic behaviour is the blood-regurgitating vampire bat, which feeds undernourished bats in their group to avoid starvation [33]. Velvet monkeys exhibit similar behaviour to their groups by giving alarm calls to warn of the presence of predators, putting themselves at risk in doing so [34]. The type, level and results of altruistic behaviour vary widely between organisms, as do the relationships evolved between helper and beneficiary actors.

The AHPSO algorithm incorporates a kind of conditional altruistic behaviour in which particles in a behaviourally heterogeneous population can lend and borrow ‘energy’, with such transactions depending on the lender’s judgment of how ‘credit-worthy’ a borrower is. These mechanisms generate more effective exemplars than in the canonical PSO, delaying the loss of population diversity and preventing premature convergence, resulting in a highly efficient search algorithm.

The performance of the new BEPSO and AHPSO algorithms was verified over 30, 50 and 100 dimensions of the widely used CEC’13, CEC’14 and CEC’17 benchmark test suites, along with a set of 14 constrained real-world problems, compared against a demanding set of 13 well-known state-of-the-art PSO variants, the 2014 CEC competition winner, L-SHADE (a powerful differential evolution algorithm) and the recent bio-inspired I-CPA metaheuristic. The overall results of this very thorough comparative investigation show that both BEPSO and AHPSO are statistically significantly superior to a large majority of the comparator algorithms and highly competitive against all others, particularly on high-dimensional complex problems (none of the other algorithms were statistically superior to the new algorithms on such problems). In addition, they are shown to be very strong candidates for constrained real-world applications, with BEPSO having the highest mean rank of all the test algorithms on the suite of constrained problems. Both BEPSO and AHPSO achieved robust, high-quality performance across the entire range of test problems and suites *with a single set of parameter values*; they did not need tuning for each new type of problem. Both algorithms were shown to maintain diversity in the population without sacrificing efficient convergence to optimal solutions.

Section 2 discusses related work; then Section 3 describes the proposed algorithms in detail. Section 4 details the experimental method. This is followed by Section 5, in which we present the results of the extensive comparative experiments, and the paper closes with discussion and conclusions in Section 6 and Section 7.

## 2. Related Work

### 2.1. Bio-Inspired Search Algorithms

There has been a surge in the development of bio-inspired search algorithms in the last few decades due to the applicability of these methods across a wide variety of domains and problems. A part of this surging interest is due to the limitation of traditional gradient-based algorithms. In contrast to gradient-based algorithms, bio-inspired algorithms are not gradient-dependent and are significantly less sensitive to the initial solution.

Two of the main research topics in bio-inspired algorithms have been avoidance of premature convergence and sensitivity to/dependence on control parameters. Work in these areas has led to the proposal of many variants of the basic canonical bio-inspired algorithms to address these issues [35,36,37,38,39,40,41].

Various recent and matured bio-inspired algorithms include the genetic algorithm [42], the wasp algorithm [43], the shark algorithm [44], ant colony optimisation [45], particle swarm optimisation [9], bacterial foraging optimisation [46], cuckoo search [47], artificial bee colony search [48], the firefly algorithm [49], the bat algorithm [50], flower pollination algorithms [51], artificial plant optimisation [52], the squirrel search algorithm [53] and the mayfly optimisation algorithm [54]. Whilst many of the newly published algorithms are typically benchmarked solely on artificial test problems, various recently published and matured algorithms have successfully been applied to real-world problems [55,56,57,58,59,60,61,62,63,64,65,66].

Due to the vast and ever-accumulating number of newly published bio-inspired algorithms in the literature, Kar [67] divided bio-inspired algorithms into four quadrants, namely the zone of theory development, zone of applications, zone of rediscovery and zone of commercialisation. *Quadrant 1* includes algorithms that are most recently published and not sufficiently studied in all dimensions. Hence, the absence of literature on such work provides potential for researchers to further improve and investigate these algorithms. Algorithms within *quadrant 2* are more mature in relation to theoretical development and have significant potential for researchers to apply them to novel areas. *Quadrant 3* encapsulates algorithms that were introduced but were overlooked or failed to attract sufficient attention from researchers. Similarly, revisiting the theoretical foundations and practical aspects of these algorithms to improve, hybridise, ensemble or apply them to novel domains may be of interest to researchers. Finally, *quadrant 4* captures algorithms that have already been extensively applied to different domains. Although it may be more challenging for researchers to find an unexplored application of these algorithms, at the same time, these algorithms have greater potential to be adopted and applied to real-world applications, as they are proven. The work presented in this paper is probably best characterized as falling within *quadrant 1*.

Many of the most competitive bio-inspired algorithms also fall under the umbrella of swarm intelligence. PSO and its variants are probably the most widely used class of swarm intelligence algorithms.

### 2.2. Particle Swarm Optimisation

In the canonical PSO [9,68], particles represent a solution in a D-dimensional search space, and each particle possesses three attributes, namely its position, memory of its best position so far and a velocity, as denoted by vectors $x_{i}$, ${\mathrm{pbest}}_{i}$ and $v_{i}$, respectively. Initially, each particle’s velocity and position are randomly assigned, and subsequently, at each time step, the fitness function is employed to guide particles towards a combination of two ‘exemplars’, namely ${\mathrm{pbest}}_{i}$ and gbest, the latter corresponding to the best position known to the swarm at time *t*. At each time step, the velocity and position of each particle are updated using the following two equations:

(1) $$v_{i}^{\left(t+1\right)}=\omega v_{i}^{\left(t\right)}+c_{1}r_{1}\left({\mathrm{pbest}}_{i}-x_{i}^{\left(t\right)}\right)+c_{2}r_{2}\left(\mathrm{gbest}-x_{i}^{\left(t\right)}\right)$$

(2) $$x_{i}^{\left(t+1\right)}=x_{i}^{\left(t\right)}+v_{i}^{\left(t\right)}$$

where ω is an inertia weight parameter that reflects the impact of the previous velocity on the new velocity, an important factor in achieving a balance between global exploration and local exploitation [68]. In addition, $c_{1}$ and $c_{2}$ are the ‘cognitive’ and ‘social’ acceleration coefficients, where the cognitive coefficient controls the local search (guided by exemplar ${\mathrm{pbest}}_{i}$), whilst the social component controls global exploration (guided by exemplar gbest) and represents a kind of cooperation between the particles. The control of these two coefficients is important in performing am efficient search, as excessive values of $c_{1}$ would lead to excessive wandering of the particles and, similarly, an excessive value of $c_{2}$ would lead to premature convergence of the swarm. $r_{1}$ and $r_{2}$ are random D-dimensional vectors, with each component generated in the range of [0,1], powering the stochastic element of the search.

The novel search algorithms introduced in the next section build upon this basic framework. They employ heterogeneous populations (not all members following the same rules as in the canonical version) and use more complex ways of calculating the dynamic exemplars, among other developments.

As an indication of their effectiveness, PSO algorithms have been successfully applied to various practical problems in the last few decades [69,70,71,72,73,74,75,76,77].

As mentioned in the previous section, in common with various other bio-inspired algorithms, one of the main limitations of the canonical PSO algorithm is premature convergence that originates from loss of population diversity. PSO variants that address premature convergence and related issues tend to propose mechanisms that periodically revamp or maintain population diversity throughout the search process [14,15,78,79]. However, the development of mechanisms to maintain high population diversity while concurrently enabling rapid convergence to optimal or near-optimal solutions is an area that has only been partially explored and remains an active field of research. It is exactly this area to which the new algorithms proposed in this paper belong; they use novel mechanisms that are shown to maintain diversity and drive rapid convergence to optimal solutions.

## 3. Proposed Algorithms

In this section, the two novel PSO search algorithms, BEPSO and AHPSO, are explained in detail. For both, the aim was to design heterogeneous particle behaviours that maintain diversity and provide an efficient search.

### 3.1. BEPSO: Biased Eavesdropping PSO

In BEPSO, the bio-inspired particle behaviour model comprises the following three components: recognition, communication and bias. The recognition component refers to the particle’s ability to distinguish between conspecific and heterospecific particles. The communication component refers to the implicit signal-based communication particles perform when they discover a new and better position. The bias component enables particles to build a form of perception towards each other that evolves through social experiences. This perception is used to adopt different behaviours during the search process.

**Figure 1:**
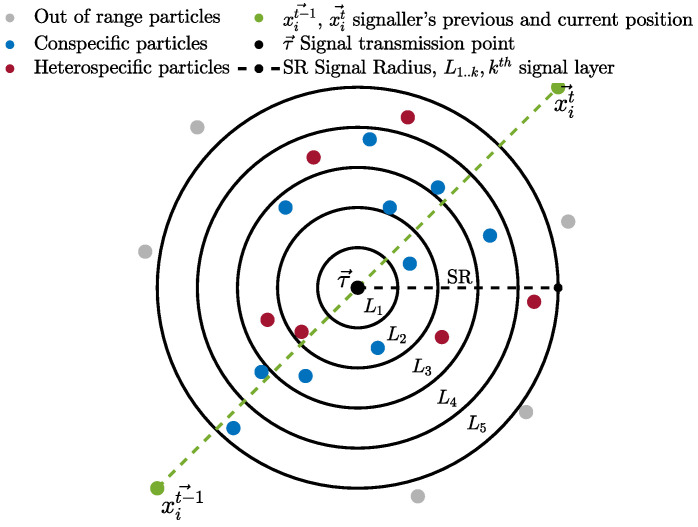
Visual representation of the communication signal sent by the signaller particle in the BEPSO algorithm.

Initially, particles are divided into two groups. Particles of the same group recognise each other as conspecifics and those from the other group as heterospecifics. All particles are assigned an initial random bias towards the rest of the particles in the swarm in such a fashion that any two conspecific particles may be either negatively biased or unbiased towards each other, while any two heterospecifics may be positively biased towards each other. In the first cycle of the algorithm, particle search behaviour initiates by updating velocity and position using the canonical PSO algorithm’s update equations (Equations (1) and (2)). Thereafter, the more complex update rules given below take over. After the positional update, if a particle discovers a better position, in an attempt to guide conspecifics to a potentially better location, the particle communicates with the surrounding conspecific particles by transmitting a signal indicating the new location. The signaller particle intends the communication signal recipients to be solely conspecifics, but surrounding heterospecific particles eavesdrop and exploit the information in the signal (as detailed later). The signal recipients (from both conspecific and heterospecific groups) either accept or reject the information provided by the signal, considering several factors, including their bias towards the signaller particle. Before transmitting the signal, the particle determines the transmission point (τ) for the intended communication signal. τ is a position that lies between the previous and the current position of the signaller particle and is calculated using Equation (3).

(3) $$\tau_{i}^{t}=x_{signaller}^{\;t-1}+rand*\left(x_{signaller}^{\;t}-x_{signaller}^{\;t-1}\right)$$

where $x_{signaller}^{t}$ and $x_{signaller}^{\;t-1}$ are the current (newly discovered) and previous position of the signaller particle, respectively, and rand is a uniformly distributed random number in the range of (0,1). The communication signal has a radius defined by the SR parameter with minimum and maximum bounds. $SR_{max}=0.01*\left(UB-LB\right)*d$, and $SR_{min}=0.001*\left(UB-LB\right)*d$, where UB and LB are the upper and lower bounds of the given problem, respectively, and *d* is the dimension of the problem. The radius of the communication signal is determined individually for each signal based on the particle’s fitness compared to the average fitness in the swarm. This simulates the signal’s loudness; hence, the range of the signal extends or shrinks based on the quality of the discovered position. This behaviour mimics the confidence of the particle in the quality of the discovered location to attract more conspecifics. Hence, a confident signaller particle transmits a signal with a wider influence range, while particles with less confidence in the quality of the discovered position use lower SR with the intention of transmitting a signal to fewer conspecifics, thereby minimising the potential loss of fitness in the conspecific population. The SR parameter is calculated using Equation (4).

(4) $$SR\left(\psi \left(x\right),\bar{\psi },SR_{min},SR_{max}\right)=\left\{\begin{array}{cc} rand(SR_{min},\frac{SR_{min}+SR_{max}}{2}) & \psi \left(x\right)<\bar{\psi }\\ rand(\frac{SR_{min}+SR_{max}}{2},SR_{max}) & else, \end{array}\right.$$

where ψ(x) is the fitness of a signaller particle (to be maximised, in the case of function minimisation, this is inversely proportional to the function evaluation value (f(x))), $\bar{\psi }$ is the average fitness in the swarm at time *t* and rand(n1,n2) is a random number in the range of (n1,n2). This ensures that fitter particles shout louder (have higher SR). The communication signal is modelled using k signal layers to mimic environmental noise and the distortion of the signal as it travels out towards the boundary of the signal range. Hence, recipient particles located in different locations relative to the signal “hear” differently distorted variants of the original signal (newly located position). Figure 1 shows a visual depiction of the communication signal with intended conspecific recipients and eavesdroppers. We mutate the signal vector *k* times (for *k* signal layers) with a non-uniform Gaussian mutation operator, starting with a small mutation (*p* = 0.1), and as *k* increases, increasing the mutation probability to trigger larger mutations, with an upper bound of 1. This ensures that the further away a particle is, the more distorted the signal it receives. Any particle whose Euclidean distance from the transmission point (τ) is less than SR is a recipient of the signal (see the algorithm pseudocode). The upper and lower bounds for *p* were chosen after preliminary experiments, as values outside that range were found to be ineffective. Other researchers have found that *p* = 0.1 is a good lower bound for the non-uniform mutation operator when it is used to increase diversity [17].

Particles (both conspecific and eavesdroppers) can accept or ignore the information provided by the communication signal, depending on their bias and the signaller particle’s confidence in the newly discovered position (detailed later). The recipient particles closest to the transmission point receive the least distorted signal, and those furthest away receive the most distorted signal. This set of stochastic mechanisms–distorting the signal as it travels across the search space and placing the transmission point between the current and last location–prevents multiple particles from clustering in exactly the same location while encouraging movement towards confidently signalled better regions, helping to avoid stagnation within recipient particles.

Among animals, survival and cooperation strongly depend on bias towards others, either through genetic influence, e.g., cooperating with a conspecific for the first time, regardless of any lack of previous experience, or through social learning, where positive association plays a role. In our algorithmic model, particles are either positively biased, negatively biased or neutral (unbiased) towards each other and use their bias to decide whether to accept or reject the information the signal provides as a guide. Negative bias can be thought of as the particle’s defence mechanism built over time to avert potential negative social guidance and, thus, minimise the loss of fitness due to misleading communication signals. Positive bias, on the other hand, enables particles to form an implicit cooperative relationship and accept guidance through signal calls from established “social partners” formed over time in an attempt to improve fitness. The two mechanisms complement each other and allow particles to form decentralised communication that evolves based on particles’ individual social experiences. Particles’ biases form over time as a result of the accumulation of consecutive positive or negative experiences resulting from the use of signal information. An experience is positive when the signal improves the fitness of the recipient particles and negative when it reduces it. The accumulator decision model from the free-response paradigm [80] is employed to enable particles to accumulate evidence through signaller–recipient relationships. When either of the two accumulated experience variables (α (positive) and β (negative); Equations (5) and (6), respectively) reaches a threshold, a decision response is triggered to accept or reject a signal. The following update equations are used to build the bias “evidence” between a recipient and a signaller particle (assuming minimisation of the fitness function (f(x))):

(5) $$\alpha_{ji}^{t}=\left\{\begin{array}{cc} \alpha_{ji}^{t-1}+\lambda^{t} & f{\left(x_{j}\right)}^{t}\leq f{\left(x_{j}\right)}^{t-1}\\ \alpha_{ji}^{t-1} & f{\left(x_{j}\right)}^{t}>f{\left(x_{j}\right)}^{t-1} \end{array}\right.$$

(6) $$\beta_{ji}^{t}=\left\{\begin{array}{cc} \beta_{ji}^{t-1} & f{\left(x_{j}\right)}^{t}\leq f{\left(x_{j}\right)}^{t-1}\\ \beta_{ji}^{t-1}-\lambda^{t} & f{\left(x_{j}\right)}^{t}>f{\left(x_{j}\right)}^{t-1} \end{array}\right.$$

where $\alpha_{ji}^{t}$ is the positive bias variable and $\beta_{ji}^{t}$ is the negative bias response variable at time *t* that the *j*th particle (recipient) has collected for the *i*th particle (signaller), and $\lambda^{t}$ is the accumulating factor that contributes to the bias response variables as follows:

(7) $$\lambda^{t}={\left(f{\left(x\right)}^{t}-f{\left(x\right)}^{t-1}\right)}^{2}\times 0.01$$

If the experience is positive, α increases; if it is negative, β decreases. The multiplicative factor of 0.01 in Equation (7) was chosen after preliminary experiments conducted with a range of possible values.

**Figure 2:**
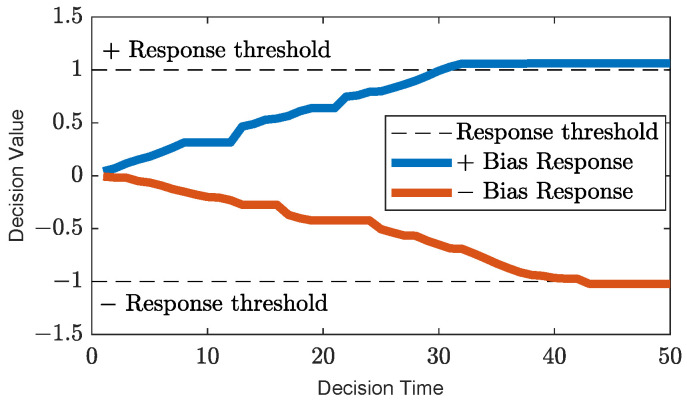
The accumulator decision model showing the *i*th particle’s bias towards the *j*th particle at time *t*.

Figure 2 shows a visual depiction of Equations (5) and (6) unfolding over time. Each time evidence is collected, Equation (8) is used to determine if either of the response variables (α or β) has reached the specified bias threshold value (the α threshold is positive, and the β threshold is negative).

(8) $$Bias_{i}^{j}=\left\{\begin{array}{cc} 1, & \alpha_{ji}^{t}\geq \eta \\ -1, & \beta_{ji}^{t}\leq -\eta \\ 0, & else \end{array}\right.$$

where $Bias_{i}^{j}$ is the *j*th particle’s bias towards the *i*th particle, and the threshold (η) is an integer in the range [10, 100]. The value of η controls the pace at which particles become biased; hence, the value of η can have a direct impact on the behaviour of particles Particles tend to be rapidly biased when η is set in the lower range. On the contrary, particles can remain unbiased towards most other particles in the swarm for extended periods when η is in the higher range.

At the beginning of the search process, all particles are given random biases (positive (1), negative (−1) or neutral (0)) to allow for heterogeneity from the start of the search process. Since there would be no transmission of signals at t=1, all particles initially update their velocity and position using the standard PSO update equations (Equations (1) and (2)). After the positional update, if the particle discovers a better position, it must transmit a communication signal to attract conspecifics to a potentially better location using the procedures described above.

To try and avoid costly ‘mistakes’ due to misleading information, receiving particles—both conspecifics and surrounding eavesdropping heterospecifics—decide to exploit or ignore the signal information according to a simple risk-versus-reward assessment. The following rules define the criteria both conspecifics and eavesdroppers use to exploit or ignore the signal information:

1. Conspecific recipient particles decide to exploit signal information only if the recipient particle is positively biased or unbiased towards the signaller and the signaller particle’s confidence in the newly discovered position is high.
2. Eavesdropper particles decide to exploit signal information if the eavesdropper is positively or negatively biased towards the signaller but the signaller’s confidence in the newly discovered position is high.

The signaller particle’s confidence is high if $SR>=(\left(SR_{min}+SR_{max}\right))/2$ and low if $SR<(\left(SR_{min}+SR_{max}\right))/2)$.

In nature, animals adopt various strategies to deter eavesdroppers or make their signals less desirable or noticeable to heterospecifics. In this study, the signaller particle aims to evade eavesdroppers by adopting a probabilistic strategy whereby it occasionally deliberately uses a smaller SR value to attract fewer particles (see algorithm psuedocode for details). This behaviour enables signallers to appear less confident in the quality of the discovered position to make the signal less conspicuous for eavesdroppers. This evasion strategy mostly affects eavesdroppers, even whilst negatively biased towards the signaller particle, because they place weight on the signaller’s confidence. However, it comes at a cost to conspecifics of the signaller, as it narrows the range, meaning fewer receive the signal.

Both conspecific and eavesdropper recipients that adopt the signal-based guidance use the following equation to update their velocity:

(9) $$v_{i}^{t+1}=\omega v_{i}^{t}+c_{1}^{t}r_{1}\left({\mathrm{pbest}}_{i}-x_{i}^{t}\right)+c_{2}^{t}r_{2}\left(L_{k}-x_{i}^{t}\right)$$

where $L_{k}$ is the signal vector for the *k*th layer of the signal (the appropriate layer relative to the distance between the signaller and recipient), and the other symbols are as used in Equation (1).

**Figure 3:**
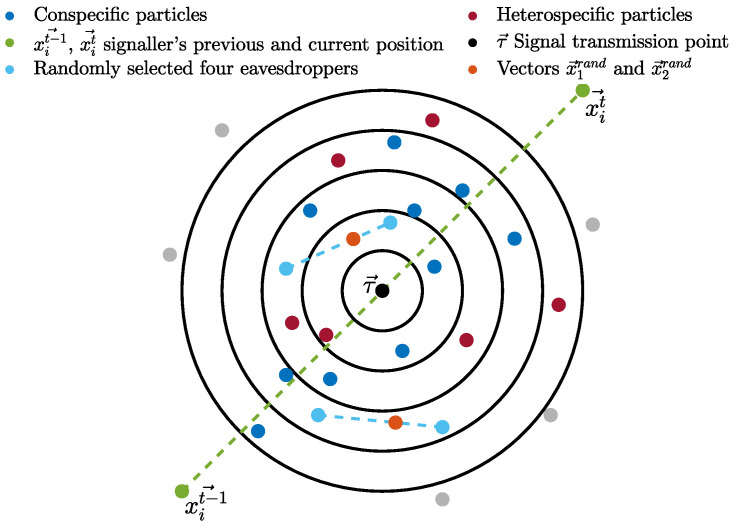
Visual depiction of the particles selected for the collaboration exemplar.

Non-signal-based behaviour uses two repellent exemplars and a collaboration exemplar. The two repellent exemplars ($x_{fur}^{t}$ and $x_{OoR}^{t}$) are the particle located farthest from the signaller and a (randomly selected) non-recipient particle that is outside of the signal range. The two repellent exemplars are selected from the recipient’s conspecific group. The collaboration exemplar ($x_{coll}^{t}$) requires the random selection of four recipients from the other group (eavesdroppers). It is calculated as shown in Equation (10).

(10) $$\begin{array}{c} x_{coll}^{t}=\bar{R}=\sum_{i=1}^{2}\frac{x_{i}^{rnd}}{2},\\ x_{1}^{rnd}=x_{1}^{ed}+rand*\left(x_{2}^{ed}-x_{1}^{ed}\right),\\ x_{2}^{rnd}=x_{3}^{ed}+rand*\left(x_{4}^{ed}-x_{3}^{ed}\right) \end{array}$$

where $x_{1}^{ed}$–$x_{4}^{ed}$ are the four randomly selected eavesdropper particles within the signal range. $x_{1}^{rnd}$ is a vector that lies between the positions of the first and second selected recipient particles, and, similarly, $x_{2}^{rnd}$ lies between the third and fourth selected recipients, as illustrated in Figure 3. $x_{coll}^{t}$ is the average of the two vectors.

Particles that adopt the non-signal-based guidance update their velocities using the following equation:

(11) $$v_{i}^{t+1}=\omega v_{i}^{t}+c_{1}^{t}r_{1}\left({\mathrm{pbest}}_{i}-x_{i}^{t}\right)+c_{2}^{t}r_{2}\left(S-x_{i}^{t}\right)$$

where S is an exemplar randomly selected as either $x_{fur}^{t}$, $x_{OoR}^{t}$ or $x_{coll}^{t}$.

Imitation is one of the most common social learning behaviours that animals adopt. In our algorithmic model, the unbiased recipients imitate the dominating behaviour of their conspecifics. Hence, if *p* particles of the conspecifics or eavesdroppers adopt the signal-based guidance while *q* of them adopt the non-signal-based guidance and p>q, then the unbiased conspecifics/eavesdroppers imitate the behaviour dominantly adopted by their conspecifics. When p=q or unbiased particles dominate one or both groups, signal-based or non-signal-based behaviour is randomly adopted by the unbiased recipient particles.

The heterogeneity in the swarm is formed by the mix of signal-based and non-signal-based behaviour adopted by recipient particles. The particles’ biases formed over time maintain the balance of particles adopting these behaviours. The entitlement of particles as recipients depends on several factors, including the previous position, the position discovered position by the signaller, the SR value and the calculated transmission point. Hence, a small fluctuation in one of these factors significantly alters the list of potential recipients of both conspecifics and eavesdroppers at time *t*. Consequently, which set of particles become recipients is unpredictable for each transmitted signal. This unpredictable yet self-organising behaviour is a further support for population diversity, minimising the risk of particles being stuck at local optima.

In order to fully exploit existing potential solutions, the BEPSO model also incorporates the periodic use of multi-swarms, as introduced in [29]. Every so often, the swarm is split into multiple swarms, which supports another phase of search, after which they join back together, the standard BEPSO mechanisms resume and the cycle repeats.

The initiation of the multi-swarm mechanism requires the division of the swarm into *N* subswarms. Instead of randomly splitting the swarm into *N* equal subswarms, in our method, N=3 subswarms are formed based on particles’ dominating biases to enable each subswarm to possess an asymmetrical and self-regulating population. Hence, a particle is a member of $subswarm_{1}$ if it is predominantly positively biased towards most particles in the swarm. Similarly, if a particle is mostly negatively biased or unbiased, then the particle belongs to the corresponding groups ($subswarm_{2}$ and $subswarm_{3}$). Each member of a subswarm uses the following equation to update its velocity:

(12) $$v_{i}^{t+1}=\omega v_{i}^{t}+c_{1}^{t}r_{1}\left({\mathrm{pbest}}_{i}-x_{i}^{t}\right)+c_{2}^{t}r_{2}\left({\mathrm{lbest}}_{k}-x_{i}^{t}\right)$$

where ${\mathrm{lbest}}_{k}$ is the position of the fittest particle in the kth subswarm.

### 3.2. BEPSO Summary

The BEPSO algorithm uses a heterogeneous population that is split into two groups. Members of a group recognise each other as conspecifics and members of the other group as heterospecifics. When a particle finds a better position (solution), it generates a signal to guide conspecifics towards the new solution. The signal range is proportional to the quality of the solution, and the signal is distorted as it travels through space. The transmission point of the signal is randomly chosen between the last and the current position of the transmitting particle. Heterospecific particles in range can eavesdrop on the signal. Particles have biases towards each other, which determine whether or not a particle decides to adopt or ignore the signal information based on an accumulator decision model. These interacting stochastic mechanisms determine the social exemplars for particles in their movement update equations.

Particles’ biases build over time, ensuring behavioural heterogeneity by forcing particles to dynamically adopt signal-based and non-signal-based guidance. This primary search strategy is supported by periodically activated subswarm searches to efficiently exploit the existing solutions. The asymmetrical populations in the subswarms exploit different local solutions with varying densities of particles in different search phases, resulting in efficient search behaviour with a good balance of exploration and exploitation. The various interacting mechanisms maintain diversity and prevent premature convergence while allowing for rapid, efficient movement towards optimal and near-optimal solutions. The overall BEPSO algorithm is shown in Algorithm 1.

#### BEPSO Parameters

The BEPSO algorithm involves a number of parameters, but our intention was to develop a technique that does not need tuning for each new problem it is applied to. Hence, after extensive preliminary parameter investigations involving a wide range of problems and problem types and sizes, the following set of parameters was found to be robust and effective and was adopted for all subsequent experiments reported in this paper. Note that, in common with many current PSO algorithms, the momentum term (ω) is adaptive and time-varying, as detailed in Algorithm 1. Full details of the extensive parameter investigations can be found in the Supplementary Material.

The population size = 40, the length of the two phases of the search (signalling and multi-swarm) = 10 iterations, $SR_{max}$ = 0.01, $SR_{min}$ = 0.001, and bias threshold = 20 (for other parameters, see Algorithm 1).
**Algorithm 1:** BEPSO
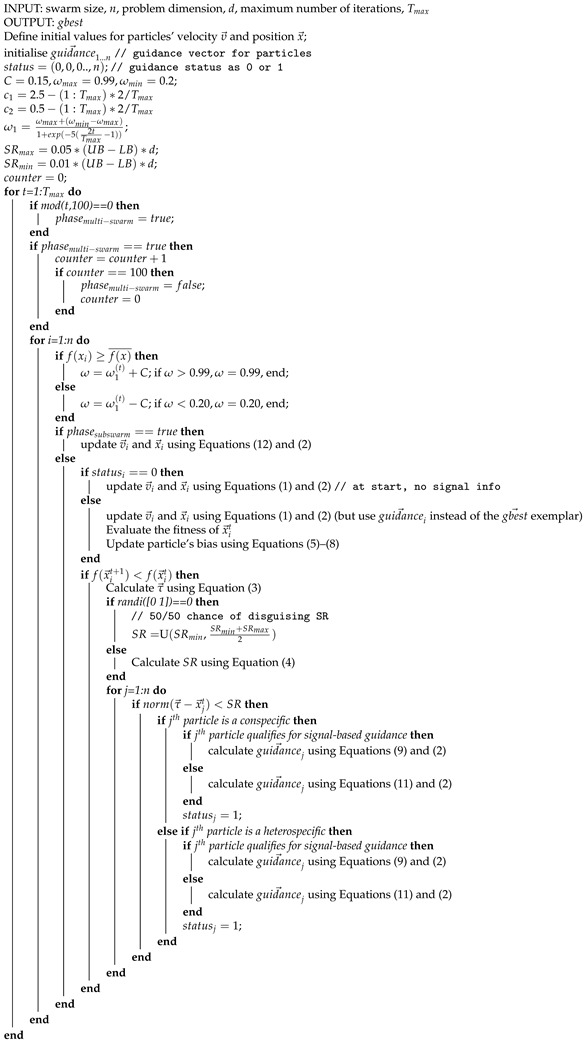


### 3.3. AHPSO: Altruistic Heterogeneous PSO

In this section, we describe our second novel PSO algorithm inspired by a certain kind of altruistic animal behaviour, a direction that has not been explored before.

The AHPSO algorithm incorporates conditional altruistic behaviour and a heterogeneous particle model that delays the loss of population diversity and prevents premature convergence, resulting in a highly effective search algorithm. In our approach, particles are conceptualised as energy-driven entities with two possible states, namely active and inactive. Particles have a current energy level and an activation threshold and are inclined to be active by maintaining their current energy level above the threshold. The distinction in a particle’s state is used to create a heterogeneous population, and particles’ tendency to become active is aided by lending–borrowing relationships among them. Hence, conditional altruistic behaviour is exhibited by particles by lending energy to inactive particles to allow them to change their state. In doing so, helper/lender particles risk downgrading their own state from active to inactive. To minimise the risk of reducing their own fitness, a group of lenders assesses the situation of the beneficiary/borrower particle based on the level of altruistic behaviour it has exhibited before making a decision as to whether or not to lend. In our behavioural model, heterogeneity is attained through altruism, and better population diversity is achieved through heterogeneity. Hence, these two concepts depend on, feed and maintain each other.

In AHPSO, two behaviour models are used. The first is the altruistic particle model, which constitutes the particles’ primary behavioural model. The second, the paired particle model, extends the former to boost population diversity further. They are applied one after the other in each overall cycle of the AHPSO algorithm.

### 3.4. Altruistic Particle Model (APM)

The activation status of particles is dependent on their current energy level ($E_{current}$) and activation threshold ($E_{activation}$). Initially, both values are randomly assigned. The concept of activation is employed to determine the type of movement strategy for particles at the individual level and, as a result, controls behavioural heterogeneity in the swarm.

Particles have an inherent tendency to be active; hence, particles in the inactive state are expected to borrow energy from other particles when their $E_{current}<E_{activation}$. The main factor influencing and maintaining swarm heterogeneity is the particles’ altruistic behaviour. A particle that behaves altruistically by making significant energy contributions to other swarm members is highly unlikely to be rejected when in need of energy itself, and on the contrary, particles that exhibit lower altruistic behaviour are inclined to be rejected.

Persistent borrowing behaviour in a particle over prolonged periods results in a highly unstable lending–borrowing ratio and reduces the altruism value ($A_{i}$) of the particle (as the particle consumes a lot more resources than it contributes to the swarm). $A_{i}$ is calculated according to Equation (13).

(13) $$A_{i}=\frac{L_{i}^{t}}{B_{i}^{t}}$$

where $L_{i}^{t}$ and $B_{i}^{t}$ are the number of times the ith particles lent and borrowed energy, respectively, up to time *t*. When a particle is unable to activate, it attempts to borrow energy from randomly selected potential lenders, and in order to lend energy, potential lender particles expect the energy-requesting particle to meet an altruism criterion defined by ϕ (Equation (14)).

(14) $$\phi =P_{1}\times P_{2}$$

This criterion is based on the independent probability of two events ($P_{1}$ and $P_{2}$). For $P_{1}$, the altruism value of the borrower particle ($A_{i}$) is used, and $P_{2}$ is calculated as

(15) $$P_{2}=\frac{\delta }{N}$$

where δ is the number of particles in the swarm with active status at time *t*, and *N* is the population size.

$P_{2}$ gives a rough measure of the probability that a lender particle will return the energy lent by the swarm. In addition to enforcing altruistic behaviour, the criterion (ϕ) provides a form of altruistic assessment of lender particles’ probability of returning lent energy.

Potential lender particles use the γ value described by Equation (16) to inform the final decision to either lend energy or reject the request of the borrower particle.

(16) $$\gamma \left(\phi ,\beta \right)=\left\{\begin{array}{cc} false, & \mathrm{if}\;\phi <\beta \\ true, & \mathrm{if}\;\phi \geq \beta \end{array}\right.$$

where β is the average altruism value in the swarm.

If the decision (γ) is in favour of the energy-requesting particle (i.e., true), an equal amount of energy is borrowed from each lender to compensate for the required energy of the borrower particle. This is calculated as

(17) $$E_{required}=\frac{E_{activation}-E_{current}}{\alpha }$$

where $E_{required}$ is the amount of energy required from each lender and α is the number of selected lenders. The movement strategy adopted by particles in the altruistic behaviour model is based on the altruistic traits of particles. Particles that are active use the canonical PSO update equation shown in Equations (1) and (2), whereas inactive particles who do not meet the criterion (ϕ) and, therefore, cannot borrow use Equation (18) to update their velocity (and position via Equation (2)).

(18) $$v_{i}^{t+1}=\omega v_{i}^{t}+c_{1}r_{1}\left({\mathrm{pbest}}_{i}-x_{i}^{t}\right)+c_{2}r_{2}\left({\mathrm{pbest}}_{minA}^{t}-x_{i}^{t}\right)$$

where ${\mathrm{pbest}}_{minA}^{\left(t\right)}$ is the personal best position of the least altruistic particle at time t. In the AHPSO framework, particles that do not meet the criterion (ϕ) are less altruistic at time *t* and, hence, behave together with similarly less altruist particles. Considering the evolving dynamics of the altruistic model, the least and most altruistic particles fluctuate. Hence, guidance towards the least altruistic particle partially enables cooperation through altruism and supports heterogeneity. Energy sharing takes place between the lender particles and the borrower who meets the criterion (ϕ). As lenders are randomly selected without any criteria, there is a distinct possibility of some lenders not having excess energy to lend. Therefore, after borrowing energy, the borrower particle may still lack sufficient energy to activate. In this case, an exemplar for the particle is generated by the mean position of half of the lender particles, and their velocities are updated according to Equation (19).

(19) $$v_{i}^{t+1}=\omega v_{i}^{t}+c_{1}r_{1}\left({\mathrm{pbest}}_{i}-x_{i}^{t}\right)+c_{2}r_{2}\left(x_{mean}-x_{i}^{t}\right)$$

where $x_{mean}$ is the mean position of the randomly selected $\left\lceil \frac{\alpha }{2}\right\rceil$ lender particles.

As commonly seen in certain PSO variants, in our behavioural model, particles do not explicitly exchange positional information; hence, by using the mean position of a proportion of lender particles, we aim to establish implicit communication between lender and borrower particles.

If, however, the borrower particle succeeds in borrowing sufficient energy to activate, the particle’s velocity is calculated using Equation (20).

(20) $$v_{i}^{t+1}=\omega v_{i}^{t}+c_{1}r_{1}\left({\mathrm{pbest}}_{i}-x_{i}^{t}\right)+c_{2}r_{2}\left({\mathrm{pbest}}_{maxA}^{t}-x_{i}^{t}\right)$$

where ${\mathrm{pbest}}_{maxA}^{\left(t\right)}$ is the personal best position of the most altruistic particle at time *t*. The *i*th particle is guided towards the most altruistic particle to establish partial cooperation and maintain heterogeneity. An additional stochastic element is introduced by randomly reinitiating $E_{current}$ and $E_{activation}$ for the entire swarm at specific intervals. The idea behind this is to fluctuate the altruism value of particles and allow less altruistic particles at time *t* to cooperate, contribute and evolve as altruistic particles. In contrast, an altruistic particle could “devolve” and exhibit selfish behaviour. As a result, this model allows altruistic and selfish particles to adopt distinct movement strategies that change and adapt depending on the level of a particle’s “evolution”, leading to an adaptive and heterogeneous particle population.

### 3.5. Paired Particle Model (PPM)

The paired particle model is an extension of the altruistic behaviour model described in the previous section. The purpose of the PPM is to further boost the heterogeneity properties of the algorithm, leading to increased population diversity. The PPM is run after the APM in each overall iteration of AHPSO. A relatively small proportion of the population is used for the paired particle model (see Section 3.6 for values). This model employs two movement strategies for the selected particles, namely a coupling-based strategy and an opposition-based strategy; each pair randomly selects which to use (see Algorithm 2). The paired particle model enables particles to randomly form and maintain pair-style bonds similar to the mechanism employed in [81]. An altruistic particle may abandon its pair if the pair is less altruistic than the swarm’s average.

#### 3.5.1. Coupling-Based Strategy

The coupling-based strategy distinguishes pairs as tightly or loosely coupled or neutral, which determines the type of movement strategy. The following rules govern the type of coupling relationship paired particles adopt:

1. A pair is tightly coupled if both particles are active at time *t*.
2. A pair is loosely coupled if both particles are inactive at time *t*.
3. A pair is neutral if one particle is active and the other is inactive at time *t*.

Tightly coupled paired particles tend to have more influence on each other than loosely coupled pairs. Tightly and loosely coupled particles update their velocities using Equation (21) and Equation (22), respectively.

(21) $$v_{i}^{t+1}=\omega v_{i}^{t}+c_{1}r_{1}\left(\left(x_{pair}^{i}\times E_{current}^{i}\right)-x_{i}^{t}\right)+c_{2}r_{2}\left(\left({\mathrm{pbest}}_{pair}^{i}\times E_{current}^{i}\right)-x_{i}^{t}\right)$$

where $x_{pair}^{i}$ and ${\mathrm{pbest}}_{pair}^{i}$ are the *i*th particle’s pair position and personal best position, respectively.

(22) $$v_{i}^{t+1}=\omega v_{i}^{t}+c_{1}r_{1}\left(\left({\mathrm{pbest}}_{pair}^{i}\times E_{current}^{i}\right)-x_{i}^{t}\right)+c_{2}r_{2}\left(\left(x_{pair}^{i}\times E_{current}^{i}\right)-x_{i}^{t}\right)$$

$E_{current}^{i}$ is used as a damping factor to prevent the possibility of particles rapidly oscillating, instead performing small movements in this secondary phase of the search. In essence, the coupling-based strategy empowers particles within the paired behaviour model to influence each other, regardless of any distance constraints between pairs. Therefore, clustered particles take small steps towards their pair, depending on the type of coupling relationship formed, causing perturbations in the current position without an explicit impact on pbest. However, these fluctuations in particle position subsequently influence the next position of the particle, helping to escape local optima.

#### 3.5.2. Opposition-Based Strategy

The opposition-based movement strategy guides paired particles towards exemplars with opposite features. By guiding both particles of a pair in potentially distinct directions, the strategy aims to maintain diversity within such pairs and, hence, within a proportion of the population. In a way, this movement strategy partly compensates for the limitations of previously described coupling-based strategy, where pairs influence each other. The opposition-based strategy aims to slow down learning between pairs without destroying it and delays the loss of diversity between pairs by guiding both in the direction of distinct exemplars. The altruism value of the paired particles is used as the determining factor to distinguish the type of movement a particle performs. Exemplar selection for members of paired particles works as follows. If the *i*th particle is more altruistic than its coupled pair, its velocity is updated using Equation (23).

(23) $$v_{i}^{t+1}=\omega v_{i}^{t}+c_{1}r_{1}\left({\mathrm{pbest}}_{i}-x_{i}^{t}\right)+c_{2}r_{2}\left(x_{maxA}^{pair}-x_{i}^{t}\right)$$

where $x_{maxA}^{pair}$ is randomly selected as either pbest or the position of the most altruistic individual of the most altruistic pair at time *t*.

But if the *i*th particle is less altruistic than its pair, its velocity is updated using Equation (24).

(24) $$v_{i}^{t+1}=\omega v_{i}^{t}+c_{1}r_{1}\left({\mathrm{pbest}}_{i}-x_{i}^{t}\right)+c_{2}r_{2}\left(x_{minA}^{pair}-x_{i}^{t}\right)$$

where $x_{minA}^{pair}$ is randomly selected as either pbest or the position of the least altruistic individual of the least altruistic pair at time *t*.

Since both movement strategies in the paired particle model always result in particles moving, they act as a stabilising mechanism that enables particles to partially escape from local optima and continue the search process. In both coupling-based and opposition-based learning, the fitness of the exemplar particles is deliberately not considered; this helps to minimise particle clustering around local optima, aiming to maintain diversity and, hence, guard against premature convergence.

The overall AHPSO algorithm is shown in Algorithm 2; note that an adaptive, time-varying momentum term (ω) is employed.

### 3.6. AHPSO Summary

The AHPSO algorithm uses a heterogeneous population in which a dynamic energy-based ecosystem develops. Particles are either active or inactive, depending on their energy level. All particles have an inherent drive to become active and, when inactive, attempt to borrow energy from other (randomly selected) particles in order to reach the activation threshold. The altruism of a particle develops over time, depending on its lending and borrowing behaviour. Particles use a model based on the altruism level of a potential borrower in order to decide whether or not to lend—a form of conditional altruism. Particles’ movement strategies depend on their levels of activation and altruism, which are controlled by social exemplars generated by interacting stochastic rules. The algorithm uses two search phases, in which different movement rules apply.

The two phases of AHPSO, which are executed consecutively in each cycle, namely the altruistic and paired particle models, complement each other. The search process is initiated with the APM, during which particles attempt to change their state from inactive to active. Behaviourally, active particles tend to be more focused on exploitation. On the contrary, inactive particles, attempting to borrow energy, are more focused on exploration and are mainly influenced by the most and least altruistic particles at time *t*. The level of altruistic behaviour exhibited by particles varies a great deal, and particles evolve frequently from more to less or less to more altruistic. Hence, the different types of particles (active, successful borrowers and unsuccessful borrowers) are guided by highly diverse and rapidly evolving exemplars, enabling efficient search behaviour while maintaining population diversity.

Next, the second behaviour model (PPM) takes over. Unlike the main APM, this is used for only a selected proportion of the population. The main purpose of the PPM is to further increase heterogeneity and prevent stagnation of particles for the selected proportion of the population. Because energy and altruism levels play a part in the movement strategies, changes in the values of $E_{current}$ and $A_{i}$ shape particles’ behavioural patterns and result in stochastic switching between different types of movements, leading to diverse behaviour and an evolution of the strategy. The result, as with BEPSO, is a highly effective balance between exploration and exploitation.
**Algorithm 2:** AHPSO
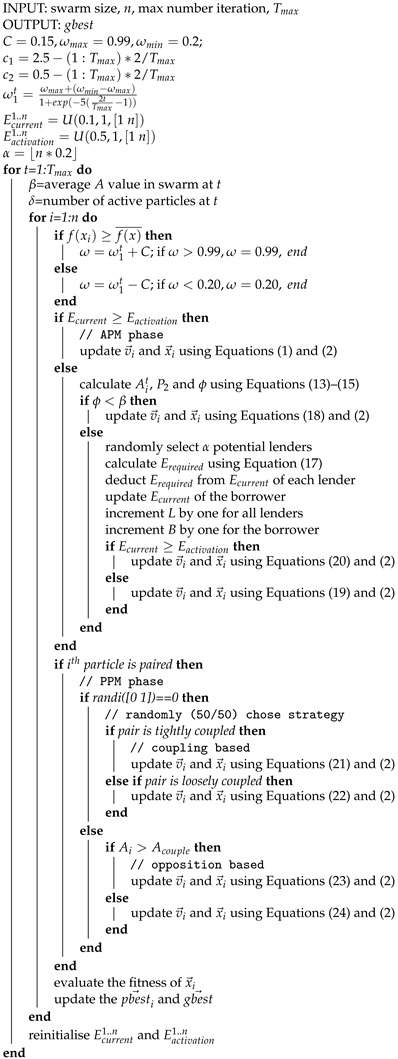


#### AHPSO Parameters

The AHPSO algorithm involves a number of parameters, but, like with BEPSO, our intention was to develop a technique that works very well across a wide range of problems and problem sizes with a single general set of parameters. Hence, after extensive preliminary parameter investigations, the following robust set of parameters was found to be highly effective and was adopted for all subsequent experiments reported in this paper. Full details of the extensive parameter investigations can be found in the Supplementary Material.

The population size = 60, α is randomly set in the range of [10,18] each time it is used, the period after which the lender and borrower profiles of the swarm are reset is set to $LB_{rate}$ = 10 in order to avoid stagnation, the period after which energy and energy activation values are reinitialised is ER = 5 and the paired population size = 6 (see Algorithm 2 for other details). The preliminary investigations also established that employing the secondary PPM phase of the search had a significantly positive impact.

## 4. Experimental Method

The performance of the new BEPSO and AHPSO algorithms was verified across multiple dimensions (30, 50 and 100) of the widely used CEC’13 [82], CEC’14 [83] and CEC’17 [84] benchmark test suites, along with various constrained real-world problems. A thorough comparison was conducted against 13 well-known state-of-the-art PSO variants; a recent bio-inspired metaheuristic (I-CPA); and the 2014 CEC competition winner, L-SHADE (a powerful differential evolution algorithm). Each of the comparator algorithms used the best published general parameter set.

The CEC test suites comprise unconstrained single-objective benchmark problems of various classes, including unimodal, multimodal, hybrid and composition functions. The CEC’13 suite comprises a total of 28 functions, namely 5 unimodal, 15 multimodal and 8 composition functions. The CEC’14 suite comprises 30 functions, namely 3 unimodal, 13 multimodal, 6 hybrid and 8 composition functions. The CEC’17 suite comprises 29 functions, namely 1 unimodal, 7 multimodal, 10 hybrid and 11 composition functions. Overall, a total of 87 unconstrained benchmark functions were used to evaluate the performance of the algorithms, each at three different problem dimensions. These test suites are widely regarded as suitably challenging, enabling thorough evaluation of search algorithms. The evaluation process of each test suite was carried out according to the evaluation criteria set out by the official CEC competitions [84].

The algorithms were also tested on the 14 non-convex constrained real-world problems [85] listed in Table 1.

In order to produce statistically robust results, each algorithm was run 30 times on each test problem. For the CEC test suites, the maximum number of function evaluations per problem was $10^{4}\times d$, where *d* is the problem dimension.

For the constrained problems, the maximum number of function evaluations for each problem ($Max_{FEs}$) was determined using the following criteria:

(25) $$Max_{FEs}=\left\{\begin{array}{cc} 1\times 10^{5}, & D\leq 10\\ 2\times 10^{5}, & 10<D\leq 30\\ 4\times 10^{5}, & 30<D\leq 50 \end{array}\right.$$

where *D* is the dimension of the problem. A penalty method, as defined in [86,87], was used to convert the constrained evaluation functions to unconstrained evaluation functions (adding penalties proportional to constraint violations). The method is defined by Equations (26) and (27), assuming function minimisation.

(26) F(x)=f(x)+H(x)

(27) $$H\left(x\right)=\omega_{1}\delta +\omega_{2}\sigma$$

where $\omega_{1}$ and $\omega_{2}$ are static weights ($\omega_{1}$, $\omega_{1}$ = 100), δ is the number of violated constraints and σ is the sum of all violated constraints.

The full set of 15 comparator algorithms and their key parameters, as used in this study, are shown in Table 2. The same set of general algorithm parameters was used for both the unconstrained and constrained test suites.

### Computational Complexity

The metric proposed in [82] was used to calculate computational complexity (see Section 6) using the following steps (originally designed for the CEC13 suite) for each required dimension:

*Step 1*—Calculate the given code (according to the methodology proposed in [82]) and record the computation time as $T_{0}$.
*Step 2*—Calculate the computation time just for $F_{14}$ (CEC13 test suite) for $20\times 10^{4}$ function evaluations on dimension *D* and record the results as $T_{1}$.
*Step 3*—Calculate the complete algorithm computation time for $F_{14}$ with $20\times 10^{4}$ function evaluations on the same dimension as $T_{2}$.
*Step 4*—Repeat step 3 5 times and attain 5 individual $T_{2}$ values ($\bar{T_{2}}=mean\left(T_{2}\right)$).

Finally, the time complexity ($T_{c}$) is calculated as $T_{c}=\bar{T_{2}}-\left(T_{1}/T_{0}\right)$.

## 5. Results

This section presents the results of detailed comparative investigations of the efficacy of BEPSO and AHPSO using the methodology outlined in the previous section. All results are based on the mean of 30 runs. See the Data Availability Statement at the end of this paper for details of access to raw results data, including all convergence graphs.

### 5.1. BEPSO: Performance

**Figure 4:**
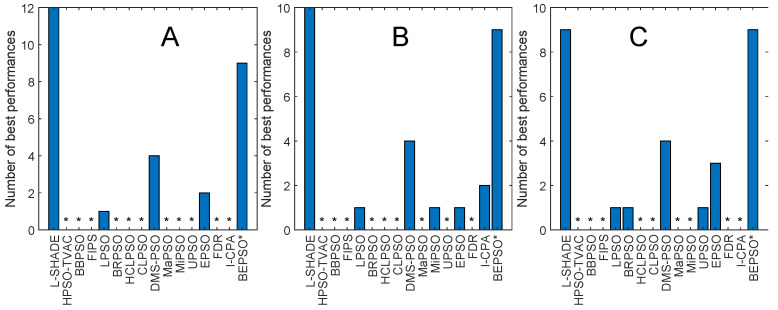
**BEPSO** comparison. The total number of best performances achieved by each algorithm with respect to mean error values (relative to best known/found function values) on the **CEC’13** problems. (**A**) 30 dimensions; (**B**) 50 dimensions; (**C**) 100 dimensions. * No best performances achieved.

**Figure 5:**
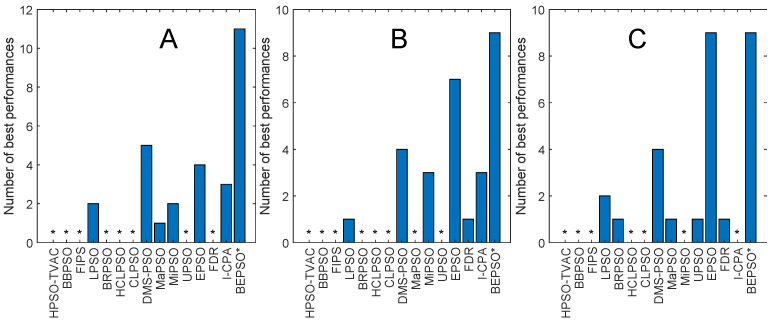
**BEPSO** comparison. The total number of best performances achieved by each PSO algorithm with respect to mean error values on the **CEC’13** problems. (**A**) 30 dimensions; (**B**) 50 dimensions; (**C**) 100 dimensions. * No best performances achieved.

Figure 4 and Figure 5 illustrate the performance of BEPSO relative to the comparator algorithms on first test suite (CEC’13) at dimensions of 30, 50 and 100. The height of the bars show how many test functions from the suite the algorithm found the best solution to (averaged over 30 runs), that is, the best solution found among all algorithms. Sometimes, multiple algorithms find the same best solution for a test function, and in other cases, they fin just one. Figure 4 compares all algorithms (including L-SHADE), and Figure 5 compares all PSO variants.

Two things are clear from these bar charts of performance on the CEC’13 test suite. BEPSO is highly competitive relative to all other comparator PSO algorithms, dominating all of them at 30-D and 50-D in terms of the number of best solutions found and all but one (EPSO, which is equal) at 100-D; it is also highly competitive in comparison to the powerful differential evolution L-SHADE algorithm, with its performance relative to L-SHADE increasing as the dimension of the problem increases (equal at 100-D).

Table 3 summarises a detailed statistical analysis of the comparative experiments across all runs at each of the three dimensions in terms of the quality of the found solutions. Pairwise statistical difference tests between BEPSO and the comparison algorithms were carried out using the non-parametric Wilcoxon signed-rank test with a significance level of 5% and appropriate adjustments for multiple comparisons [99]. The (+) symbol is used to denote the algorithms over which BEPSO exhibited statistically significantly better performance, (=) indicates no statistically significant difference in the mean performance and (−) marks the comparison algorithms whose performance is statistically significantly better than that of BEPSO. The table shows us that BEPSO’s performance on the CEC’13 test suite is significantly better than that of 10 of the 15 comparator algorithms across all dimensions, with no significant difference for the other 5. This means that none of the comparator algorithms were statistically significantly better than BEPSO on any the dimensions.

BEPSO performed particularly well on the multimodal and composition test functions of this suite, which are generally regarded as the hardest problems.

**Figure 6:**
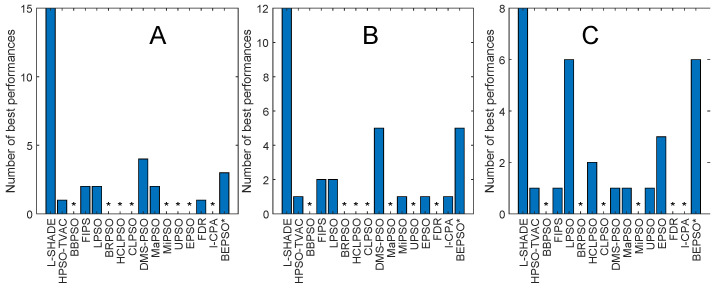
**BEPSO** comparison. The total number of best performances achieved by each algorithm with respect to mean error values on the **CEC’14** problems. (**A**) 30 dimensions; (**B**) 50 dimensions; (**C**) 100 dimensions. * No best performances achieved.

**Figure 7:**
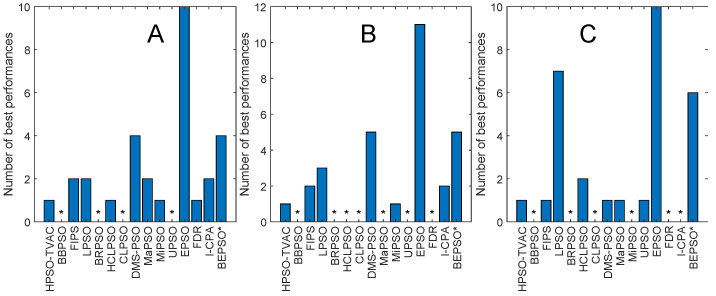
**BEPSO** comparison. The total number of best performances achieved by each PSO algorithm with respect to mean error values on the **CEC’14** problems. (**A**) 30 dimensions; (**B**) 50 dimensions; (**C**) 100 dimensions. * No best performances achieved.

Figure 6 and Figure 7 and Table 4 illustrate the corresponding results and analysis for the CEC’14 test suite. Again, we see that BEPSO compares very well with the comparator algorithms across all dimensions, if not as strongly as for CEC’13. L-SHADE is statistically significantly better, on average, than BEPSO on each dimension, and EPSO is statistically significantly better at 30 dimensions and 100 dimensions. BEPSO is statistically significantly better or equal to all other algorithms on all dimensions. Again, BEPSO performed particularly well on the multimodal and composition problems and strongly on the hybrid functions.

**Figure 8:**
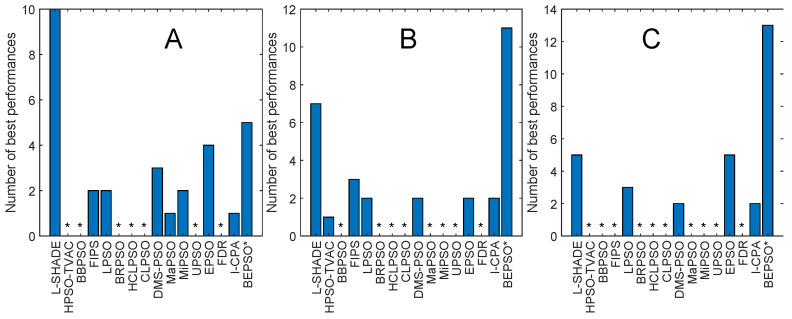
**BEPSO** comparison. The total number of best performances achieved by each algorithm with respect to mean error values on the **CEC’17** problems. (**A**) 30 dimensions; (**B**) 50 dimensions; (**C**) 100 dimensions. * No best performances achieved.

**Figure 9:**
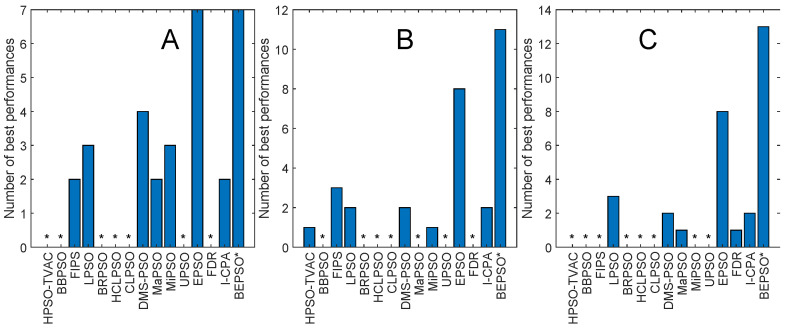
**BEPSO** comparison. The total number of best performances achieved by each PSO algorithm with respect to mean error values on the **CEC’17** problems. (**A**) 30 dimensions; (**B**) 50 dimensions; (**C**) 100 dimensions. * No best performances achieved.

Figure 8 and Figure 9 and Table 5 give the corresponding results and analysis for the CEC’17 test suite. Here, we, again, see very strong performance from BEPSO across all dimensions, becoming particularly dominant as the dimensions increases, finding more best solutions than any other algorithm at 50-D and 100-D. Table 5 shows that BEPSO is statistically significantly better than the majority of other comparator algorithms at all dimensions, beating 11 out of 15 on the higher dimensions. L-Shade is statistically significantly better at 30 dimensions, but none of the comparator algorithms is statistically significantly better than BEPSO at the higher dimensions (50-D and 100-D). Again, BEPSO performed particularly well on the multimodal and composition problems and strongly on the hybrid problems.

### 5.2. BEPSO: Convergence

**Figure 10:**
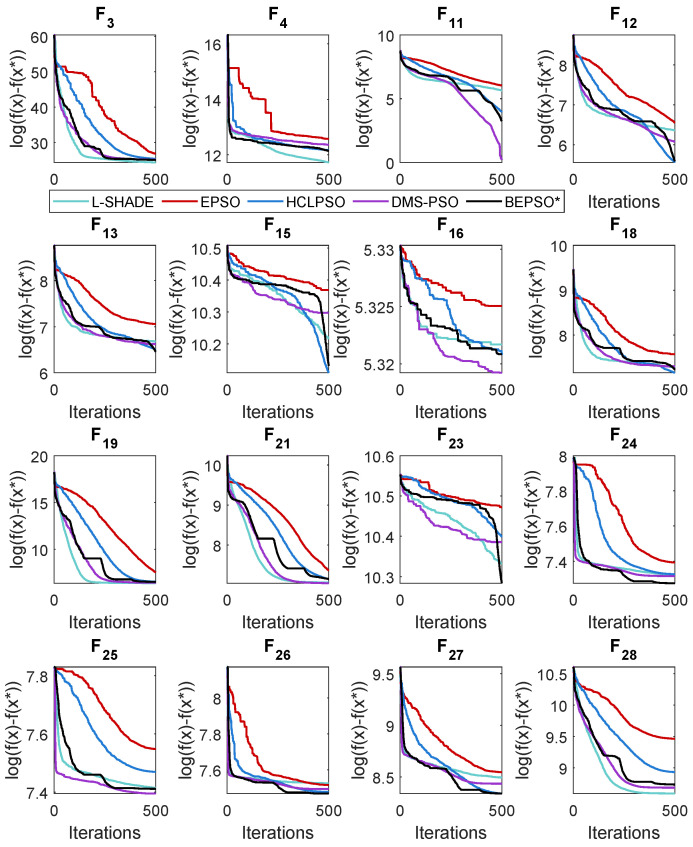
Average convergence rate comparison of BEPSO with L-SHADE, EPSO, HCLPSO and DMS-PSO on various 100-dimensional CEC’13 problems.

**Figure 11:**
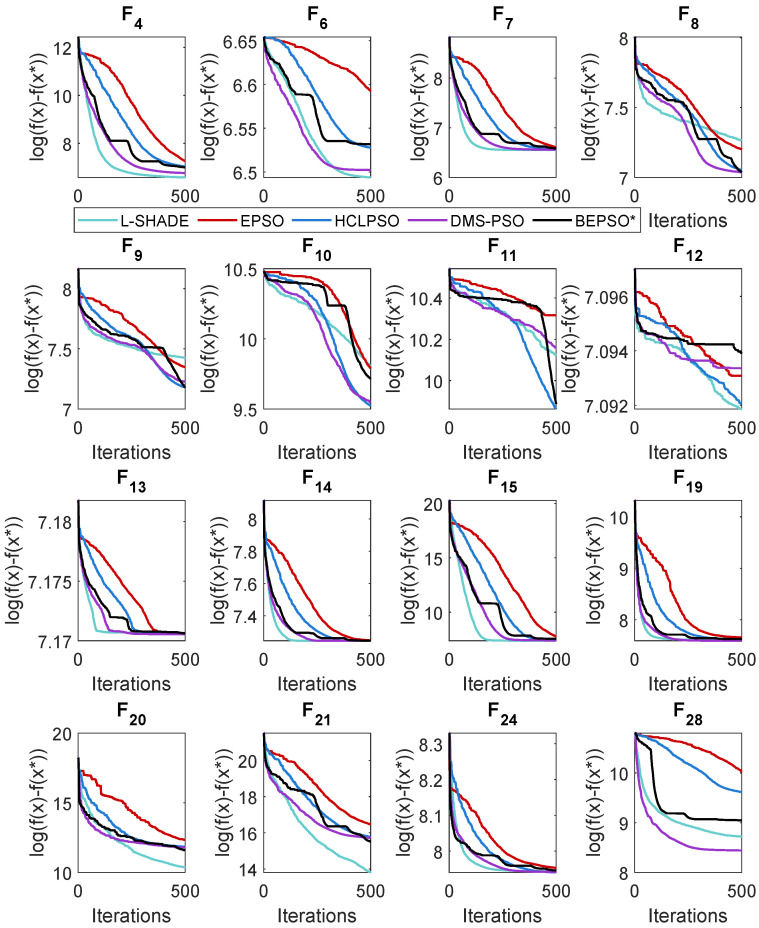
Average convergence rate comparison of BEPSO with L-SHADE, EPSO, HCLPSO and DMS-PSO on various 100-dimensional CEC’14 problems.

**Figure 12:**
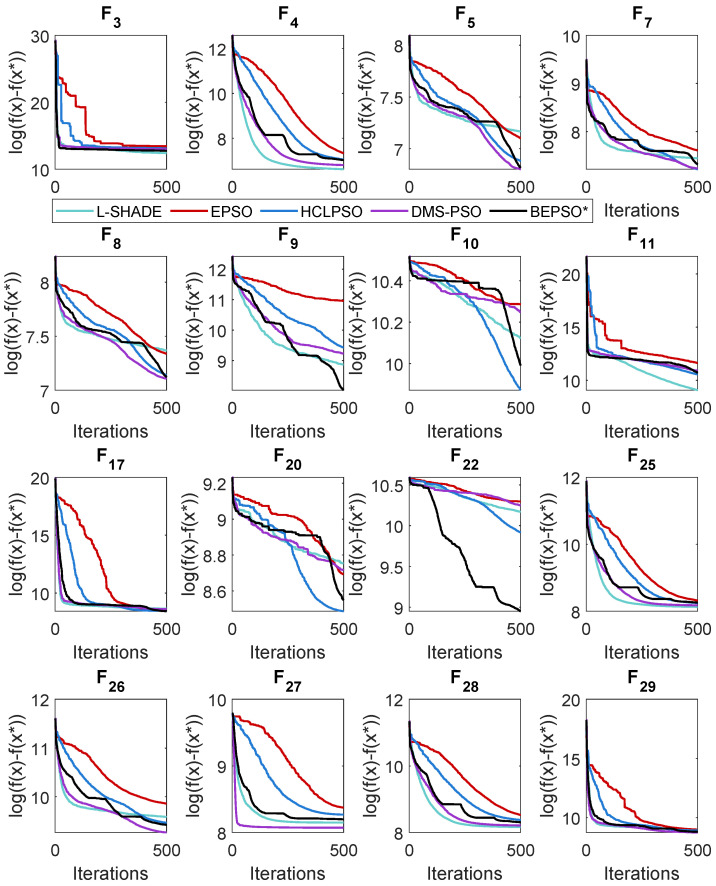
Average convergence rate comparison of BEPSO with L-SHADE, EPSO, HCLPSO and DMS-PSO on various 100-dimensional CEC’17 problems.

The analysis of the data presented in the previous subsection showed that the five consistently best-performing algorithms were (in alphabetical order) BEPSO, DMS-PSO, EPSO, HCLPSO and L-SHADE. In Figure 10, Figure 11 and Figure 12, we compare the average convergence rates towards the best solution of these algorithms across a range of representative 100-D problems for each test suite. It is clear from these figures that BEPSO’s convergence rates compare very favourably with the other top-performing algorithms. BEPSO’s rates are consistently better than EPSO and HCLPSO and very similar to those of L-SHADE and DMS-PSO.

### 5.3. AHPSO: Performance

**Figure 13:**
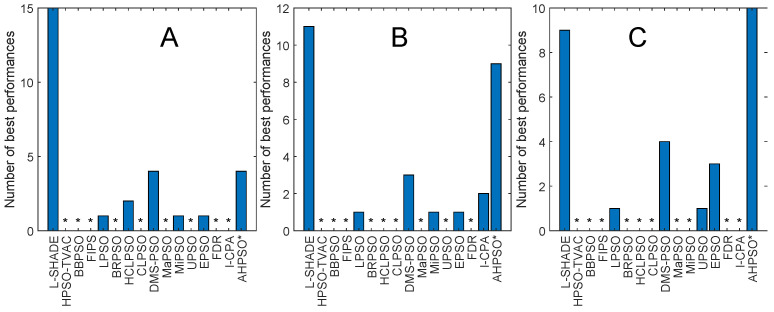
**AHPSO** comparison. The total number of best performances achieved by each algorithm with respect to mean error values (relative to best known/found function values) on the **CEC’13** problems. (**A**) 30 dimensions; (**B**) 50 dimensions; (**C**) = 100 dimensions. * No best performances achieved.

**Figure 14:**
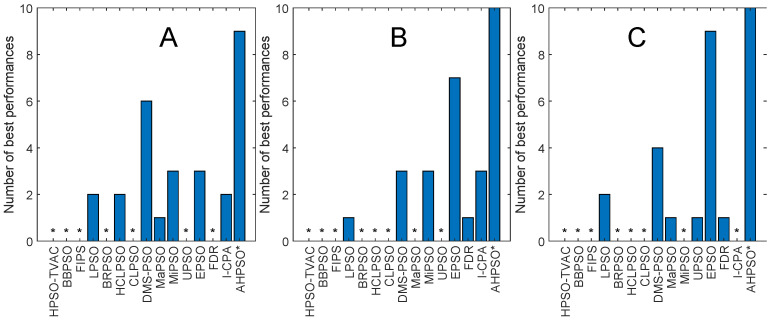
**AHPSO** comparison. The total number of best performances achieved by each PSO algorithm with respect to mean error values on the **CEC’13** problems. (**A**) 30 dimensions; (**B**) 50 dimensions; (**C**) 100 dimensions. * No best performances achieved.

**Figure 15:**
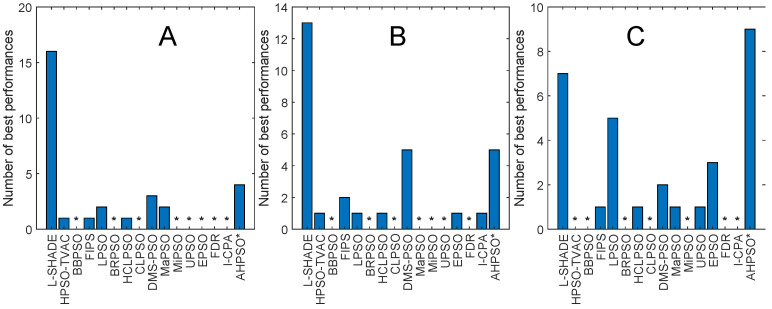
**AHPSO** comparison. The total number of best performances achieved by each algorithm with respect to mean error values on the **CEC’14** problems. (**A**) 30 dimensions; (**B**) 50 dimensions; (**C**) 100 dimensions. * No best performances achieved.

**Figure 16:**
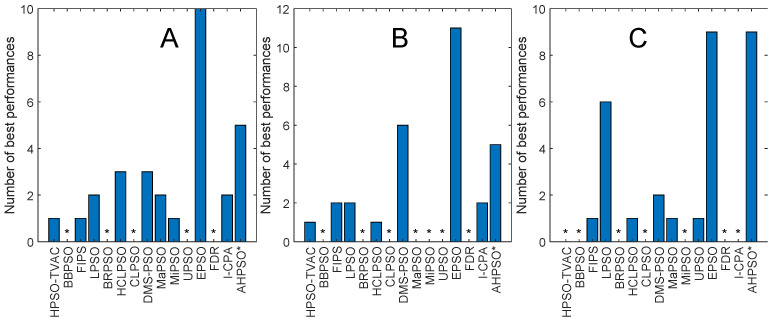
**AHPSO** comparison. The total number of best performances achieved by each PSO algorithm with respect to mean error values on the **CEC’14** problems. (**A**) 30 dimensions; (**B**) 50 dimensions; (**C**) 100 dimensions. * No best performances achieved.

**Figure 17:**
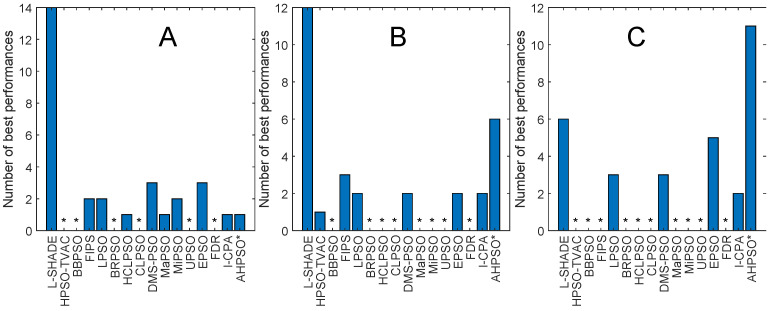
**AHPSO** comparison. The total number of best performances achieved by each algorithm with respect to mean error values on the **CEC’17** problems. (**A**) 30 dimensions; (**B**) 50 dimensions; (**C**) 100 dimensions. * No best performances achieved.

**Figure 18:**
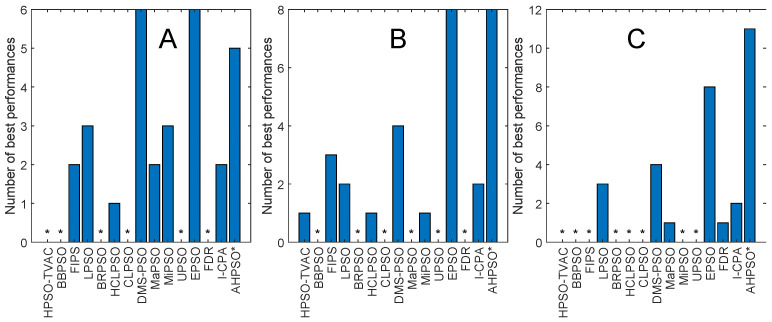
**AHPSO** comparison. The total number of best performances achieved by each PSO algorithm with respect to mean error values on the **CEC’17** problems. (**A**) 30 dimensions; (**B**) 50 dimensions; (**C**) 100 dimensions. * No best performances achieved.

Figure 13, Figure 14, Figure 15, Figure 16, Figure 17 and Figure 18 and Table 6, Table 7 and Table 8 show AHPSO’s performance on the same set of test suites against the same collection of comparator algorithms. It can be readily seen that for the CEC’13 test suite, just like BEPSO, AHPSO is highly competitive relative to all other comparator PSO algorithms, dominating all of them in terms of number of best solutions found at all dimensions, with performance also increasing as the dimensions of the problem increase (Figure 14). Its performance is also highly competitive relative to the powerful L-SHADE differential evolution algorithm at all dimensions, achieving more best solutions at 100-D (Figure 13). Table 6 shows us that AHPSO’s performance on the CEC’13 test suite is statistically significantly better than 10 of the 15 comparator algorithms across all dimensions, with no significant difference for the other 5. AHPSO performed particularly well on the composition test functions.

Figure 15 and Figure 16 and Table 7 give the results for AHPSO on the CEC’14 test suite. Relative performance is strong but not as good as for CEC’13. But we see that the increase in performance with dimensions is more marked, with AHPSO achieving more best solutions than L-SHADE at 100-D. The statistical analysis reveals that L-SHADE and EPSO were statistically significantly better at 30-D and 50-D, but AHPSO was statistically significantly better or equal to all 15 comparator algorithms at 100-D. AHPSO was superior to all other algorithms on the composition test functions.

Figure 17 and Figure 18 and Table 8 give the results for the CEC’17 test suite. Again, AHPSO’s relative performance is very strong, being statistically significantly better than 11 of the 15 comparator algorithms at the higher dimensions (50-D and 100-D), while no other algorithms were statistically better than AHPSO at these dimensions. At 30-D, only L-SHADE is statistically significantly better. AHPSO performed particularly well on multimodal and composition test functions. At 100-D, AHPSO exhibited the best performance on more functions (11 out of 29) than all other comparison algorithms (including L-SHADE).

### 5.4. AHPSO: Convergence

**Figure 19:**
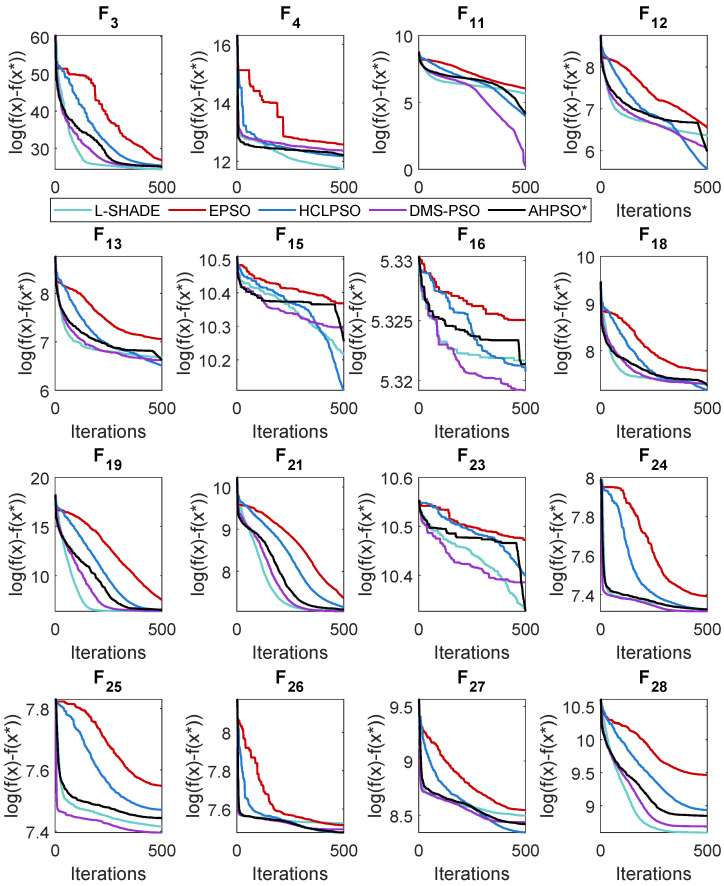
Average convergence rate comparison of AHPSO with L-SHADE, EPSO, HCLPSO and DMS-PSO on various 100-dimensional CEC’13 problems.

**Figure 20:**
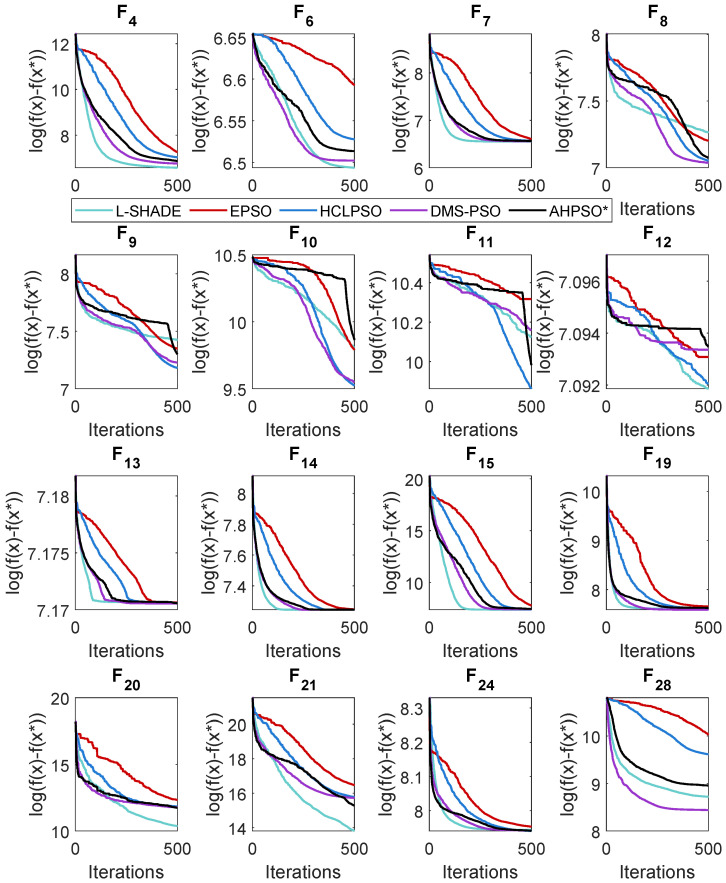
Average convergence rate comparison of AHPSO with L-SHADE, EPSO, HCLPSO and DMS-PSO on various 100-dimensional CEC’14 problems.

**Figure 21:**
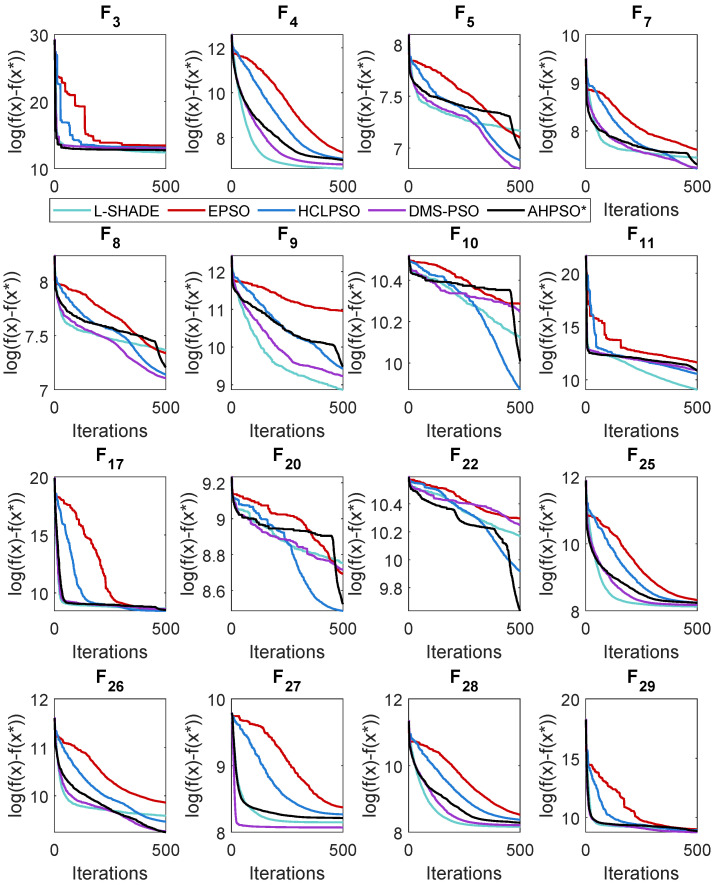
Average convergence rate comparison of AHPSO with L-SHADE, EPSO, HCLPSO and DMS-PSO on various 100-dimensional CEC’17 problems.

Like BEPSO, AHPSO was one of the consistently best-performing algorithms across all the test suites. Figure 19, Figure 20 and Figure 21 show its convergence rates relative to the other top-performing algorithms. AHPSO’s converge rates are very similar to the best rates achieved; they are consistently better than those achieved by EPSO and HCLPSO.

The comparative experiments for the two algorithms across the CEC test suites were run independently. Perhaps unsurprisingly, given the nature of the results presented above, a third set of independent runs across the CEC’13, CEC’14 and CEC’17 test suites revealed that there was no statistically significant difference in performance of BEPSO and AHPSO at 30 dimensions, 50 dimensions and 100 dimensions [100].

### 5.5. Constrained Optimisation Problems

Besides verifying the performance of the two novel algorithms on the CEC’13, CEC’14 and CEC’17 benchmark test suites, we examined the performance of BEPSO and AHPSO on 14 constrained real-world problems [101] comprising process synthesis and design, mechanical engineering and power system problems compared against L-SHADE and the 10 best PSO variants employed in the previous experiments. The complete list of constrained real-world problems is displayed in Table 1 in Section 4. Each problem was tested 30 times.

**Figure 22:**
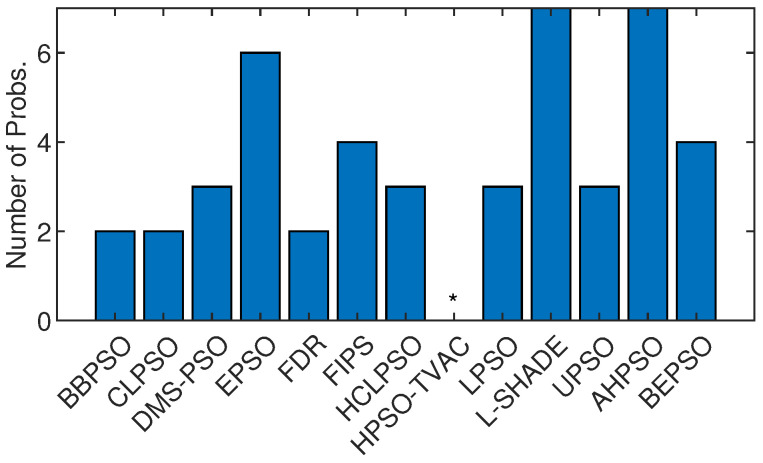
Total number of constrained real-world problems solved (lowest obtained mean error value) by the proposed and comparator algorithms. * No best solutions found.

Figure 22 shows the number of best solutions found by the algorithms across all constrained problems. By this measure, AHPSO and L-Shade perform best. AHPSO performed particularly well on the difficult power electronic problems (solving optimal pulse-width modulation problems with relatively large numbers of variables and constraints), finding the best known solution for three of the four problems in this class, with L-SHADE being the only other algorithm able to find a (single) best solution in this class. Table 9 and Table 10 show the pairwise statistical significance analysis (with appropriate reductions for multiple comparisons). It can be seen that none of the comparator algorithms is statistically significantly better than either BEPSO or AHPSO, and AHPSO is better than L-SHADE. In a detailed ranking analysis across all runs on all problems of all algorithms, the best mean rank (2.36) was achieved by BEPSO, the second best (2.5) by L-SHADE and the third best (2.57) by AHPSO.

Hence, on constrained real-world problems the two new BEPSO and AHPSO algorithms performed extremely strongly in comparison with all other comparator algorithms.

## 6. Discussion

Both BEPSO and AHPSO algorithms consistently performed particularly well on the higher-dimensional, more complex problems, including unconstrained multimodal and composition problems and problems involving many constraints, matching or bettering the performance of all other comparator algorithms. However, while their performance on simpler, lower-dimensional problems was perfectly adequate, it did not match that of the best of the comparator algorithms. This limitation seems to be associated with the deliberately high agent-level heterogeneity that is inherent to the designs of BEPSO and AHPSO. This property significantly aids the algorithms in high-dimensional and complex search spaces while being less effective in low-dimensional spaces.

**Figure 23:**
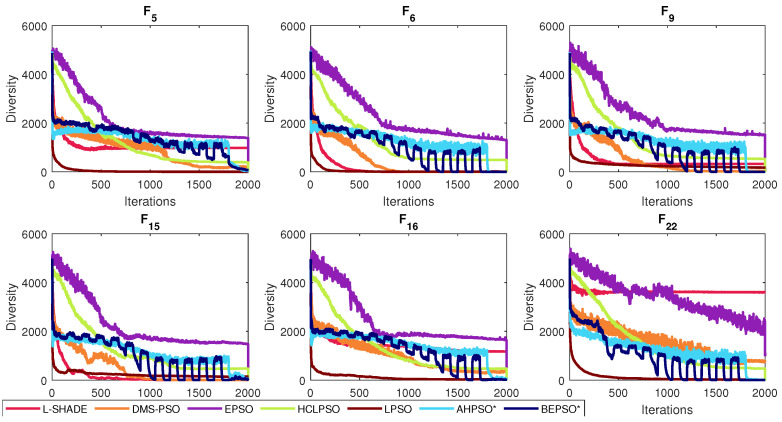
Diversity comparison for best-performing algorithms for various representative 100-dimensional CEC’17 problems.

The various stochastic mechanisms embedded in both algorithms were partly designed to maintain diversity while enabling efficient search. Figure 23 shows that both BEPSO and AHPSO achieve maintenance of diversity. The graphs use the following diversity quantification method proposed in [1]:

(28) $$Diversity\left(S\left(t\right)\right)=\frac{1}{n}\sum_{i=1}^{n}\sqrt{\sum_{d=1}^{D}{\left(x_{i}^{d}-\bar{x^{d}}\right)}^{2}}$$

where *n* is the population size, *D* is the problem dimension and $\bar{x^{d}}$ is the average value of $x^{d}$ (the dth component of the solution vector).

The figure reveals that while EPSO consistently exhibited a more diverse population compared to the other high-performing algorithms on the 100-dimensional CEC’17 problems, BEPSO and AHPSO maintained better population diversity compared to all the rest, namely L-SHADE, LPSO, DMS-PSO and HCLPSO. The trend was repeated across the other test suites for the majority of functions.

While the multiple, mainly stochastic components of the new BEPSO and AHPSO algorithms tend to complement each other and, in combination, maintain diversity; balance exploration and exploitation; and enable efficient, high-quality search, they do increase the overall complexity of the algorithm. But it is worth noting that many recent variants of metaheuristics, such as that proposed in [102,103]. and several of the comparator algorithms used in the current study have a much more complex structure than their canonical versions. These variants are discernibly more efficient and are able to handle more diverse problems. Hence, the algorithms proposed in this paper are not unusual in comprising a more complex structure and set of interaction mechanisms in comparison to canonical algorithms. However, their individual elements are all simple. Indeed, the slightly more complex framework of the proposed algorithms is inspired by the observation that biological degeneracy plays a vital role in boosting evolvability in nature and, therefore, can improve the efficiency of search processes [104]. Biological degeneracy, whereby multiple interacting mechanisms enable multiple different ways of producing an outcome, is an ubiquitous property of biological systems at all levels of organisation [105,106] reveals that systems with simple redundancy have considerably lower evolvability than degenerate (e.g., highly versatile) systems with selectable changes of behaviour, which enable compensatory actions to occur within the system (exactly what happens at the core of the new algorithms presented in this paper).

**Figure 24:**
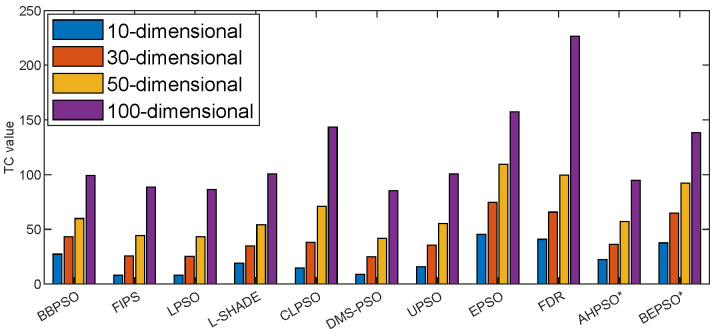
Time complexity of BEPSO, AHPSO and comparator algorithms for four dimension sizes.

However, this increased algorithmic complexity does not necessarily equate to significantly increased computational cost relative to other high-performing algorithms. Figure 24 shows the time complexity ($T_{c}$) of BEPSO, AHPSO and the comparator algorithms at various problem dimensions calculated using the metric described in Section 4.

As expected, the basic canonical PSO obtained the lowest $T_{c}$ values across all dimensions (although it is the worst-performing algorithm in terms of solution quality). However, the complexity of the high-performing AHPSO, L-SHADE and HCLPSO algorithms across all four dimensions is very similar and not much higher than that of basic PSO. BEPSO’s complexity is a little higher but still very competitive. In summary, BEPSO and AHPSO exhibit similar and, in some cases, better complexities compared to their peers; however, it is worth highlighting that they exhibit a significantly lower increase in complexity with problem dimensionality (especially AHPSO).

## 7. Conclusions

The two novel bio-inspired search algorithms presented in this paper, namely BEPSO and AHPSO, performed very well across a wide range of unconstrained and constrained optimisation problems, using a single set of parameters for all. On the CEC13 test suite, across all dimensions, both BEPSO and AHPSO performed statistically significantly better than 10 of the 15 comparator algorithms, while none of the remaining 5 algorithms performed significantly better than either BEPSO or AHPSO. On the CEC17 test suite, on the 50-D and 100-D problems, both BEPSO and AHPSO performed statistically significantly better than 11 of the 15 comparator algorithms, while none of the remaining 4 algorithms performed significantly better than either BEPSO or AHPSO. On the constrained problem set, in terms of mean rank across 30 runs on all problems, BEPSO was first, and AHPSO was third. This provides further evidence that bio-inspiration—in this case, various kinds of animal group behaviours—continues to be a very fruitful source for algorithm design.

Although both algorithms employ heterogeneous population models, are inspired by animal group behaviours and are designed to maintain diversity and avoid premature convergence and stagnation, the underlying metaphors and inspirations are very different, as are the behavioural rules used to guide particle movement. This illustrates one of the great strengths of the basic PSO framework, namely that there are numerous ways to modify and improve it, often, as in the new algorithms described here, by dynamically changing population structures and movement strategies.

Both algorithms consistently performed particularly well on the higher-dimensional, more complex problems, including unconstrained multimodal and composition problems and problems involving many constraints, matching or bettering the performance of all other comparator algorithms.

The novel mechanisms introduced in these heterogeneous PSO variants were able to maintain population diversity while enabling rapid convergence to optimal or near-optimal solutions. They provided a highly efficient balance between exploration and exploitation.

Considering the efficacy of the new algorithms on high-dimensional problems, further investigation on very large-scale problems would be another interesting direction for future work. AHPSO’s superior performance on constrained power electronic problems and the fact that other researchers have recently successfully used it to find the optimal design parameters for hybrid active power filters [107] suggest that an expanded investigation of its applications to other problems in the power electronics domain might be very fruitful.

Although canonical PSO is a very efficient algorithm for many small-scale optimisation problems, as with many metaheuristics, it suffers from the curse of dimensionality. Although the performance of the new PSO algorithms presented in this paper were much less impacted by problem dimensionality compared with other algorithms used in the experiments, various other limitations were identified for possible future improvements. It is clear that the heterogeneous nature of BEPSO and AHPSO provides various strong advantages for the general process of optimisation. However, it is this very property that makes them so good at complex (multimodal, composition, etc.), higher-dimensional problems, which means that their performance on (simpler) unimodal problems, while perfectly adequate, is consistently worse than that of some of their competitors, as their speed of finding solutions is slower. This could be accepted as a clear example of the No Free Lunch Theorem for optimization [108], which tells us that no method can be uniformly excellent on all possible problems, or it could be a prompt to try and improve the algorithms with suitable mechanisms to address this particular issue without destroying their power on other problem types. In general, unimodal problems do not require such a careful balance between exploration and exploitation; typically, intensive exploitation is expected to dominate the search process. This is a possible area for future research, either attempting to expand the general efficacy of the methods or, alternatively, to produce variants of the algorithms that are specialized to specific problem classes.

## Figures and Tables

**Table 1:** Details of the 14 constrained real-world problems. *D* is the number of decision variables; *g* and *h* are the numbers of inequality and equality constraints, respectively; and $f(x^{*})$ is the best known objective function value.

	Problem	*D*	*g*	*h*	$f(x^{*})$
Process Synthesis and Design Problems					
RC01	Process flow sheeting problem	3	3	0	1.0765430833
RC02	Process synthesis problem	7	9	0	2.9248305537
	Mechanical Engineering Problems				
RC03	Weight minimisation of a speed reducer	7	11	0	$2.9944244658\times 10^{3}$
RC04	Pressure vessel design	4	4	0	$5.8853327736\times 10^{3}$
RC05	Three-bar truss design problem	2	3	0	$2.6389584338\times 10^{2}$
RC06	Step-cone pulley problem	5	8	3	16.069868725
RC07	10-bar truss design	10	3	0	$5.2445076066\times 10^{2}$
RC08	Rolling element bearing	10	9	0	$1.4614135715\times 10^{4}$
RC09	Gas transmission compressor design	4	1	0	$2.9648954173\times 10^{6}$
RC10	Gear train design	4	1	1	0.0000000000
Power Electronic Problems					
RC11	SOPWM for 7-level inverters	25	24	1	$1.5164538375\times 10^{-2}$
RC12	SOPWM for 8-level inverters	30	29	1	$1.6787535766\times 10^{-2}$
RC13	SOPWM for 11-level inverters	30	29	1	$9.3118741800\times 10^{-3}$
RC14	SOPWM for 13-level inverters	30	29	1	$1.5096451396\times 10^{-2}$

**Table 2:** The comparator algorithms used in the detailed investigations of BEPSO and AHPSO, along with their key parameter values.

Key	Algorithm	Parameters
L-Shade [88]	SHADE with linear population reduction	$N^{init}=round\left(D\times r^{N^{init}}\right),\;\left|A\right|=round\left(N^{init}\times r^{arch}\right),\;$ $r^{arch}=2.6,\;p=0.11,\;H=6$
BBPSO [89]	Bare-bones PSO	$\phi =4.1,\;\lambda =0.729,\;c_{1},\;c_{2}=2.05,\;r_{1},\;r_{2}\;U\left(0,1\right)$
BreedingPSO [90]	A GA/PSO hybrid	w = 0.8–0.6, $c_{1},\;c_{2}=1.49445$ $V_{max}=0.15*Range$
HCLPSO [23]	Heterogeneous comprehensive learning PSO	w = 0.99–0.29, $c_{1}=2.5-0.5$, $c_{2}=0.5-2.5,\;K:3-1.5,\;V_{max}=0.5*Range$
CLPSO [22]	Comprehensive learning PSO	w = 0.9–0.2; $c_{1},\;c_{2}=1.49445,\;V_{max}=0.2*Range$
FIPS [91]	Fully informed PSO	$\chi =0.729,\;\sum c_{i}=4.1$
FDR-PSO [92]	Fitness distance ratio PSO	w = 0.9–0.4, $c_{1}=c_{2}=1,\;c=2,\;V_{max}=0.2*Range$
UPSO [93]	Unified PSO	$\chi =0.729,\;c_{1},\;c_{2}=2.05,\;NR=1$
EPSO [94]	Ensemble PSO	$w=0.9\to 0.2,\;w_{1}=0.9\to 0.4,\;c_{1}=3\to 1.5,\;$ $c_{2_{1}}=2.5\to 0.5,\;c_{2_{2}}=0.5\to 2.5,\;c_{3_{1}}=2.5\to 0.5,\;$ $c_{3_{2}}=0.5\to 2.5,\;c_{4_{1}}=2.5\to 0.5,\;c_{4_{2}}=0.5\to 2.5,\;$ $Pc=0.5,\;n_{size}=3$
DMS-PSO [29]	Dynamic multi-swarm PSO	$w=0.729,\;\;c_{1}=c_{2}=1.49445,\;m=3;\;R=15;\;$ $V_{max}=0.5*Range$
HPSO-TVAC [95]	Hierarchical PSO with time-varying coefficients	$c_{1}=2.5-0.5,\;c_{2}=0.5-2.5,\;V_{max}=0.5*Range$
LPSO [96]	Linearly decreasing inertia weight PSO	$w=0.9-0.4;\;c_{1},\;c_{2}=1.49445$
maPSO [97]	Macroscopic PSO	w=0.9−0.4, c=1.49445
miPSO [97]	Microscopic PSO	w=0.9−0.4, c=1.49445
I-CPA [98]	Improved carnivorous plant alg	nCPlant=2, nPrey=8, n=nCPlant+nPrey

**Table 3:** **BEPSO statistical tests**. Wilcoxon signed-rank test results with a significance level of p=0.05 for **CEC’13** problems at 3 different dimensions. +: statistically significantly better; =: no significant difference; −: statistically significantly worse.

BEPSO versus (CEC13)																
**Dimension**	**L-SHADE**	**HPSO-TVAC**	**BBPSO**	**FIPS**	**LPSO**	**BRPSO**	**HCLPSO**	**CLPSO**	**DMS-PSO**	**MaPSO**	**MiPSO**	**UPSO**	**EPSO**	**FDR**	**I-CPA**	+/=/−
30	=	+	+	+	+	+	=	+	=	+	+	+	=	=	+	10/5/0
*p*	0.19	$6.3\times 10^{-4}$	$3.7\times 10^{-6}$	$1.6\times 10^{-5}$	0.015	$9.2\times 10^{-6}$	0.6	$4.8\times 10^{-6}$	0.45	0.001	0.0027	$9.3\times 10^{-6}$	0.6	0.09	0.014	
50	=	+	+	+	+	+	=	+	=	+	+	+	=	=	+	10/5/0
*p*	0.19	$6.3\times 10^{-4}$	$3.7\times 10^{-6}$	$1.6\times 10^{-5}$	0.015	$9.2\times 10^{-6}$	0.6	$4.8\times 10^{-6}$	0.45	0.001	0.0027	$9.3\times 10^{-6}$	0.6	0.09	0.014	
100	=	+	+	+	+	+	=	+	=	+	+	+	=	=	+	10/5/0
*p*	0.19	$6.3\times 10^{-4}$	$3.7\times 10^{-6}$	$1.6\times 10^{-5}$	0.015	$9.2\times 10^{-6}$	0.6	$4.8\times 10^{-6}$	0.45	0.001	0.0027	$9.3\times 10^{-6}$	0.6	0.09	0.014	

**Table 4:** **BEPSO statistical tests**. Wilcoxon signed-rank test results with a significance level of p=0.05 for **CEC’14** problems at 3 different dimensions.

BEPSO versus (CEC14)																
**Dimension**	**L-SHADE**	**HPSO-TVAC**	**BBPSO**	**FIPS**	**LPSO**	**BRPSO**	**HCLPSO**	**CLPSO**	**DMS-PSO**	**MaPSO**	**MiPSO**	**UPSO**	**EPSO**	**FDR**	**I-CPA**	+/=/−
30	−	=	+	+	=	+	−	+	=	=	=	+	−	=	=	5/7/3
*p*	$7.7\times 10^{-4}$	0.23	$6.8\times 10^{-5}$	0.009	0.64	$2.0\times 10^{-5}$	0.009	$3.6\times 10^{-6}$	0.24	0.39	0.52	0.003	0.005	0.83	0.07	
50	−	=	+	+	=	+	=	+	=	=	=	+	−	=	=	5/8/2
*p*	$7.5\times 10^{-4}$	0.36	$2.1\times 10^{-5}$	$6.2\times 10^{-3}$	0.89	$1.1\times 10^{-5}$	0.23	$3.1\times 10^{-6}$	0.52	0.14	0.25	0.003	0.047	0.49	0.07	
100	−	=	+	+	=	+	=	+	=	=	=	+	−	=	=	5/8/2
*p*	0.019	0.89	$6.6\times 10^{-6}$	$3.4\times 10^{-3}$	0.56	$1.1\times 10^{-5}$	0.82	$2.0\times 10^{-5}$	0.3	0.15	0.1	$2.9\times 10^{-3}$	$9.6\times 10^{-3}$	0.7	0.15	

**Table 5:** **BEPSO statistical tests**. Wilcoxon signed-rank test results with a significance level of p=0.05 for **CEC’17** problems at 3 different dimensions.

BEPSO versus (CEC17)																
**Dimension**	**L-SHADE**	**HPSO-TVAC**	**BBPSO**	**FIPS**	**LPSO**	**BRPSO**	**HCLPSO**	**CLPSO**	**DMS-PSO**	**MaPSO**	**MiPSO**	**UPSO**	**EPSO**	**FDR**	**I-CPA**	+/=/−
30	−	+	+	+	=	+	=	+	=	+	=	+	=	+	+	9/5/1
*p*	0.019	$2.1\times 10^{-3}$	$2.5\times 10^{-6}$	$1.9\times 10^{-4}$	0.46	$2.5\times 10^{-6}$	0.58	$2.5\times 10^{-6}$	0.74	0.01	0.053	$5.7\times 10^{-6}$	0.74	0.01	$5.1\times 10^{-3}$	
50	=	+	+	+	=	+	+	+	=	+	+	+	=	+	+	11/4/0
*p*	0.54	$5.8\times 10^{-3}$	$2.4\times 10^{-6}$	$8.9\times 10^{-5}$	0.21	$2.4\times 10^{-6}$	0.03	$2.4\times 10^{-6}$	0.62	$3.8\times 10^{-3}$	$9.0\times 10^{-3}$	$2.5\times 10^{-6}$	0.74	$4.8\times 10^{-3}$	$1.7\times 10^{-3}$	
100	=	+	+	+	=	+	+	+	=	+	+	+	=	+	+	11/4/0
*p*	0.41	$3.8\times 10^{-3}$	$2.4\times 10^{-6}$	$4.5\times 10^{-5}$	0.12	$2.5\times 10^{-6}$	0.03	$2.5\times 10^{-6}$	0.29	$1.3\times 10^{-3}$	$1.2\times 10^{-3}$	$2.5\times 10^{-6}$	0.64	$4.0\times 10^{-3}$	$7.1\times 10^{-3}$	

**Table 6:** **AHPSO statistical tests**. Wilcoxon signed-rank test results with a significance level of p=0.05 for **CEC’13** problems at 3 different dimensions. +: statistically significantly better; =: no significant difference; −: statistically significantly worse.

AHPSO versus (CEC13)																
**Dimension**	**L-SHADE**	**HPSO-TVAC**	**BBPSO**	**FIPS**	**LPSO**	**BRPSO**	**HCLPSO**	**CLPSO**	**DMS-PSO**	**MaPSO**	**MiPSO**	**UPSO**	**EPSO**	**FDR**	**I-CPA**	+/=/−
30	=	+	+	+	+	+	=	+	=	+	+	+	=	=	+	10/5/0
*p*	0.09	$1.8\times 10^{-3}$	$6.0\times 10^{-6}$	$3.0\times 10^{-5}$	0.048	$1.1\times 10^{-5}$	0.95	$5.3\times 10^{-6}$	0.7	$2.7\times 10^{-3}$	$3.7\times 10^{-3}$	$1.3\times 10^{-5}$	0.49	0.21	0.018	
50	=	+	+	+	+	+	=	+	=	+	+	+	=	=	+	10/5/0
*p*	0.10	$7.4\times 10^{-3}$	$6.3\times 10^{-6}$	$2.5\times 10^{-5}$	0.043	$1.5\times 10^{-4}$	0.23	$5.2\times 10^{-6}$	0.93	0.004	0.0058	$2.3\times 10^{-5}$	0.27	0.34	0.03	
100	=	+	+	+	+	+	=	+	=	+	+	+	=	=	+	10/5/0
*p*	0.25	$3.1\times 10^{-2}$	$6.6\times 10^{-6}$	$1.3\times 10^{-5}$	0.011	$9.0\times 10^{-6}$	0.54	$5.6\times 10^{-6}$	0.95	0.003	0.003	$8.4\times 10^{-5}$	0.33	0.14	$4.3\times 10^{-3}$	

**Table 7:** **AHPSO statistical tests**. Wilcoxon Signed Rank Test Results with a Significance Level of p=0.05 for **CEC’14** problems at 3 different dimensions.

AHPSO versus (CEC14)																
**Dimension**	**L-SHADE**	**HPSO-TVAC**	**BBPSO**	**FIPS**	**LPSO**	**BRPSO**	**HCLPSO**	**CLPSO**	**DMS-PSO**	**MaPSO**	**MiPSO**	**UPSO**	**EPSO**	**FDR**	**I-CPA**	+/=/−
30	−	=	+	+	=	+	−	+	=	=	=	+	−	=	=	5/7/3
*p*	$1.9\times 10^{-3}$	0.26	$6.0\times 10^{-5}$	0.014	0.93	$1.8\times 10^{-5}$	0.034	$3.2\times 10^{-6}$	0.16	0.50	0.55	0.002	0.001	0.57	0.08	
50	−	=	+	+	=	+	=	+	=	=	=	+	−	=	=	5/8/2
*p*	$1.3\times 10^{-3}$	0.43	$2.7\times 10^{-5}$	$6.9\times 10^{-3}$	0.89	$8.7\times 10^{-6}$	0.15	$2.5\times 10^{-6}$	0.26	0.17	0.26	0.001	0.026	0.61	0.07	
100	=	=	+	+	=	+	=	+	=	+	+	+	=	=	+	8/7/0
*p*	0.23	0.28	$6.1\times 10^{-6}$	7.6E-4	0.82	$7.2\times 10^{-6}$	0.45	$3.4\times 10^{-6}$	0.68	0.03	0.016	$7.6\times 10^{-4}$	0.11	0.14	0.02	

**Table 8:** **AHPSO statistical tests**. Wilcoxon signed-rank test results with a significance level of p=0.05 for **CEC’17** problems at 3 different dimensions.

AHPSO versus (CEC17)																
**Dimension**	**L-SHADE**	**HPSO-TVAC**	**BBPSO**	**FIPS**	**LPSO**	**BRPSO**	**HCLPSO**	**CLPSO**	**DMS-PSO**	**MaPSO**	**MiPSO**	**UPSO**	**EPSO**	**FDR**	**I-CPA**	+/=/−
30	−	+	+	+	=	+	=	+	=	+	=	+	=	+	+	9/5/1
*p*	$7.7\times 10^{-4}$	$1.8\times 10^{-3}$	$2.4\times 10^{-6}$	$3.7\times 10^{-4}$	0.7	$2.9\times 10^{-6}$	0.96	$2.5\times 10^{-6}$	0.69	0.043	0.13	$8.5\times 10^{-6}$	0.39	0.028	$4.0\times 10^{-3}$	
50	=	+	+	+	=	+	+	+	=	+	+	+	=	+	+	11/4/0
*p*	0.17	$2.1\times 10^{-3}$	$2.4\times 10^{-6}$	$9.4\times 10^{-5}$	0.09	$2.5\times 10^{-6}$	0.013	$2.4\times 10^{-6}$	0.83	$2.2\times 10^{-3}$	$4.2\times 10^{-3}$	2.7×−6	0.74	$9.8\times 10^{-4}$	$3.2\times 10^{-4}$	
100	=	+	+	+	=	+	+	+	=	+	+	+	=	+	+	11/4/0
*p*	0.48	$2.0\times 10^{-3}$	$2.5\times 10^{-6}$	$5.6\times 10^{-5}$	0.14	$2.5\times 10^{-6}$	0.04	$2.5\times 10^{-6}$	0.52	$1.3\times 10^{-3}$	$1.8\times 10^{-3}$	$2.7\times 10^{-6}$	0.65	$2.4\times 10^{-3}$	$7.1\times 10^{-3}$	

**Table 9:** **BEPSO statistical tests**. Wilcoxon signed-rank test results with a significance level of p=0.05 for **constrained real-world** problems. The last row shows *p* values.

BEPSO versus (Constrained Problems)											
**L-SHADE**	**HPSO-TVAC**	**BBPSO**	**FIPS**	**LPSO**	**HCLPSO**	**CLPSO**	**DMS-PSO**	**UPSO**	**EPSO**	**FDR**	+/=/−
=	+	+	+	=	=	+	+	=	=	+	6/5/0
0.58	$1.2\times 10^{-4}$	$2.7\times 10^{-3}$	0.03	0.24	0.10	$1.2\times 10^{-3}$	$4.6\times 10^{-3}$	1.0	0.38	0.014	

**Table 10:** **AHPSO statistical tests**. Wilcoxon signed-rank test results with a significance level of p=0.05 for **constrained real-world** problems. The last row shows *p* values.

AHPSO versus (Constrained Problems)											
**L-SHADE**	**HPSO-TVAC**	**BBPSO**	**FIPS**	**LPSO**	**HCLPSO**	**CLPSO**	**DMS-PSO**	**UPSO**	**EPSO**	**FDR**	+/=/−
+	+	=	=	=	=	+	+	=	=	=	4/7/0
0.02	$1.2\times 10^{-4}$	$6.7\times 10^{-2}$	0.56	0.94	0.83	$2.6\times 10^{-2}$	$2.9\times 10^{-2}$	0.25	0.16	0.46	

## Data Availability

Code for AHPSO and BEPSO algorithms can be found at https://github.com/FTVarna [Accessed 27 August 2024]; full experimental data and can be found at https://doi.org/10.25377/sussex.26146594 [Accessed 27 August 2024]; full details of the extensive parameter investigations can be found in the Supplementary Material.

## Supplementary Materials

The following supporting information can be downloaded at: https://www.mdpi.com/article/10.3390/biomimetics9090538/s1, details of initial parameter tuning investigations.
