# Supplementary material for: Two New Bio-Inspired Particle Swarm Optimisation Algorithms for Single-Objective Continuous Variable Problems Based on Eavesdropping and Altruistic Animal Behaviours
Source: Biomimetics (Basel). 2024 Sep 5;9(9):538. doi: 10.3390/biomimetics9090538 (PMC11430302; doi:10.3390/biomimetics9090538)
Supplement: Supplementary file 1 [file biomimetics-09-00538-s001.zip › biomimetics-3106922-supplementary.pdf]

Supporting Material (Parameter tuning and analysis for BEPSO and AHPSO algorithms)
For: F. Varna and P. Husbands (2024), Two New Bio-inspired Particle Swarm Optimisation Algorithms for Single-Objective Continuous Variable Problems Based on Eavesdropping and Altruistic Animal Behaviours, submitted to Biomimetics

BEPSO Parameter Tuning and Analysis

In this section, the parameters of the BEPSO algorithm are assessed to find the *best* general set of values. The impact of the BEPSO parameters is evaluated on different dimensions of the CEC'14 and CEC'17 problem sets.

Population Size

The impact of the population size on the BEPSO algorithm was tested on the 10,30,50 and 100-dimensional CEC'14 and CEC'17 test functions. Tables 8-1:8-2 display the mean and final ranks for different population sizes and the best and second-best mean performance for 10,30 and 100-dimensional CEC'14 test functions was attained using the population size of 30 and 40, on the other hand for 50-dimensional functions, the $pop = 20$ exhibited the best and $pop = 30$ exhibited the second-best performance. The results on the CEC'17 test functions yield similar results and $pop = 20$ consistently attained the best mean performance for 30,50 and 100-dimensional functions. On the contrary, for 10-dimensional CEC'17 functions, we observe that pop=100, a higher population value, produced superior performance compared to rest of the population sizes. It is also worth noting that as shown in Figure 1-4, although pop=100 exhibited better mean performance for 10-dimensional CEC'17 functions, we observe that when $pop = 30$, the BEPSO algorithm attained lower mean values for significantly more functions and considering the marginal difference in the mean ranks (4.53 and 4.40), pop=30 may also serve as an effective population setting for 10-dimensional CEC'17 functions. Overall, we conclude that the population size of 20-40 generally perform well in most cases hence can be employed for most problems. In the comparative experiments presented in the paper, population size of 40 is employed for the BEPSO algorithm.

Table S1. Mean and final ranks of obtained using different population sizes on the 10, 30, 50 and 100 dimensional CEC'14 problems

Population	10D		30D		50D		100D	
20	4.00	3	4.67	5	2.93	1	3.73	3
30	3.73	2	3.20	1	3.43	2	3.27	2
40	3.47	1	3.63	2	3.90	3	2.83	1
50	5.03	5	3.90	3	3.43	2	4.57	5
60	4.40	4	4.50	4	4.50	4	4.47	4
70	5.33	7	4.77	6	5.33	6	5.27	6
80	5.23	6	5.57	7	5.27	5	5.70	7
100	6.13	8	6.17	8	7.07	7	6.33	8
200	6.47	9	7.40	9	7.93	8	7.63	9

Table S1- Mean and final ranks of obtained using different population sizes on the 10, 30, 50 and 100 dimensional CEC'17 problems

Population	10D		30D		50D		100D	
20	4.97	7	3.30	1	2.73	1	2.30	1
30	4.53	4	3.83	2	3.23	2	3.23	2

40	4.50	3	4.07	3	3.57	3	3.57	3
50	4.97	7	4.20	4	4.27	4	4.17	4
60	4.73	5	4.93	6	5.00	5	4.47	5
70	4.80	6	4.87	5	5.57	7	5.33	6
80	4.43	2	5.53	7	5.43	6	5.93	7
100	4.40	1	5.87	8	6.17	8	6.70	8
200	6.47	8	7.20	9	7.83	9	8.10	9

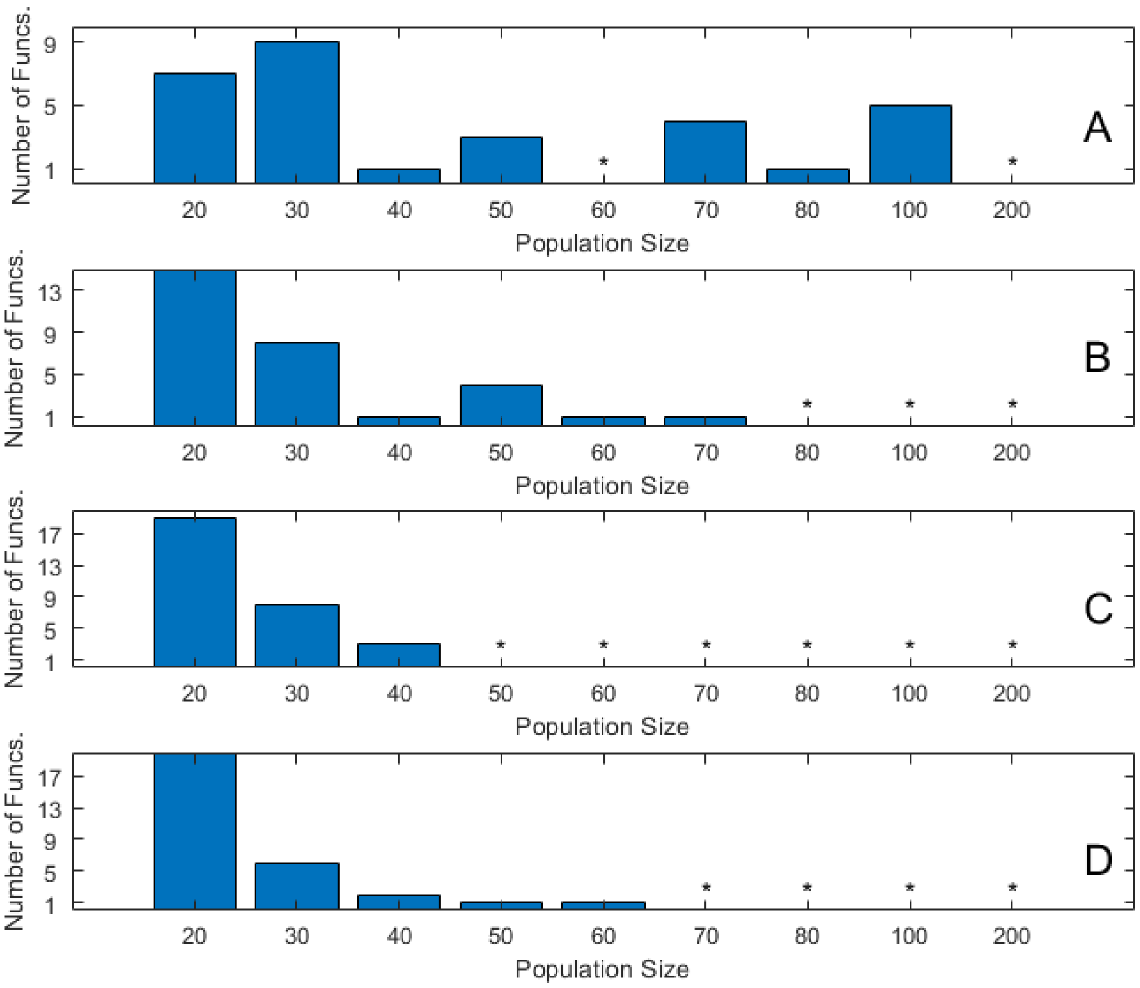

Figure S1 The total number of best performances achieved using different population sizes with respect to mean error values on the 10 (**A**), 30(**B**), 50(**D**), 100 (**D**) dimensional CEC'17 problems. * no best solutions found.

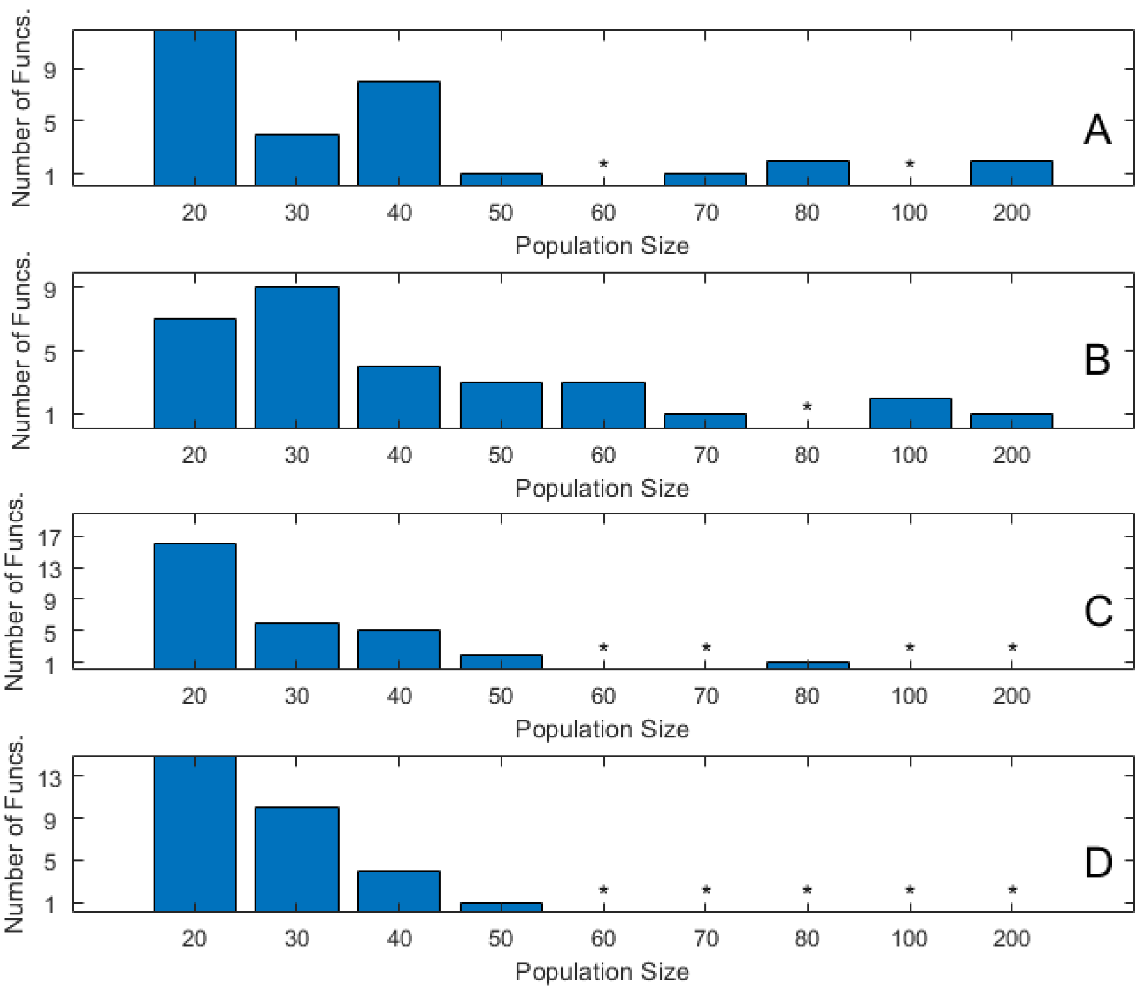

Figure S2 The total number of best performances achieved using different population sizes with respect to mean error values on the 10 (**A**), 30(**B**), 50(**C**), 100 (**D**) dimensional CEC'14 problems. * no best solutions found.

Search Phase Duration

In this section, we assess the impact of the duration of multi-swarm and non-multi-swarm search phases (the *phase* parameter). Initially, we had identified that executing both search phases equally for relatively short intervals (approximately 100 iterations) yield sufficiently good results. However, here a more extensive assessment is conducted on multiple test suites across various dimensions. We have tested 12 parameter values as intervals, the shortest interval being 10 and the longest 1000 iterations. The results obtained on the CEC'14 and CEC'17 test functions are ranked, mean and final ranks are displayed in Tables 8-3:8-4. The ranks indicate that the value of 10 attained the best performance for the entire CEC'14 test suite, and for 30 and 100-dimensional CEC'17 test functions. For 10 and 50-dimensional CEC'17 functions, phase=25 achieved the lowest ranks however $phase = 10$ also attained the second-best performance in these two cases. Hence, shorter intervals for the $phase$ parameter yield better overall mean performance on both test suites and four different dimensions. In addition, Figure 1-6 shows the lowest mean, standard deviation and minimum values each parameter value attained by for the number functions. We also observe that phase=10 not only achieved the lowest mean value for the majority of functions, but also attained the lowest standard deviation and found the lowest minimum value for the greater number of functions.

Moreover, the convergence rate for 100-dimensional CEC'17 test functions is shown in Figure 1-7. The convergence rates reveal that phase=10 consistently convergences faster to a better final solution in 19 of the 30 functions. Hence, $phase = 10$ was used for both non-multi-swarm and multi-swarm search phases for all the main comparative experiments conducted in the paper.

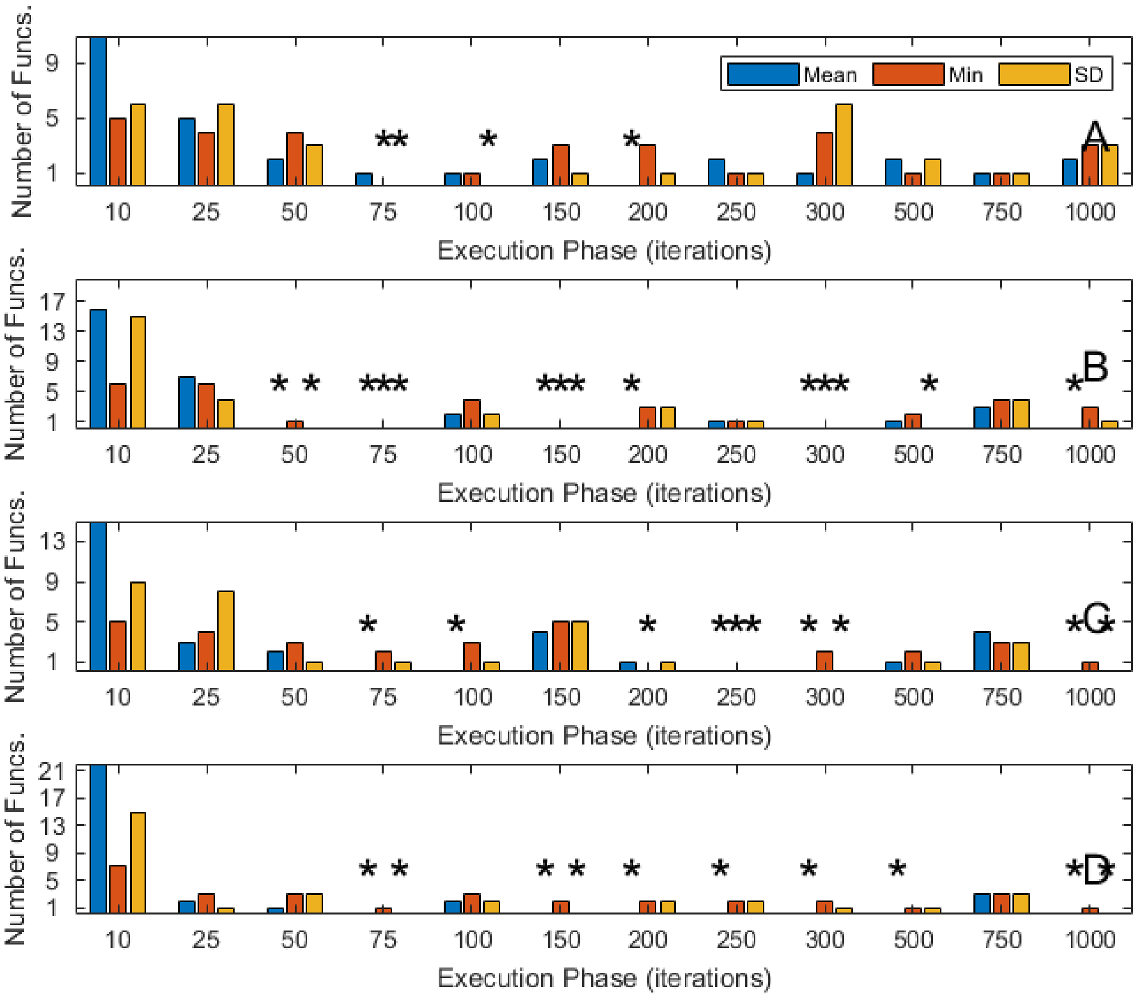

Figure S3 The impact of execution phases on obtaining the lowest mean error, min, and standard deviation values on 10 (A), 30 (B), 50 (C) and 100 (D) dimensional CEC'17 problems. * no best solution found.

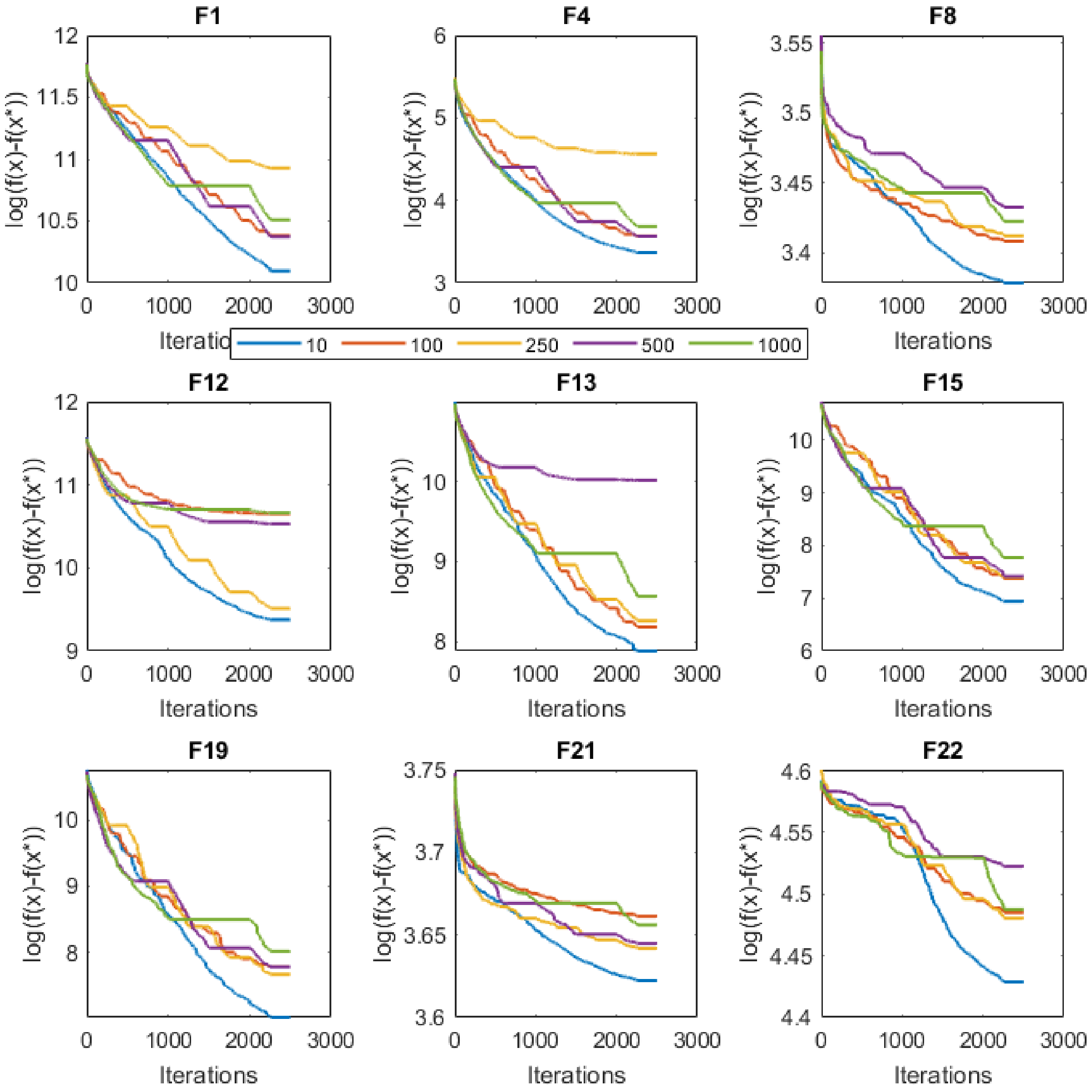

Figure S4 Impact of different execution phases on convergence rate for 100-dimensional CEC’17 functions

Table S2 Mean and final ranks obtained on the CEC'14 problems using different execution rates

Execution Phase	10D		30D		50D		100D	
10	4.67	1	3.87	1	4.60	1	3.03	1
25	5.73	3	4.93	2	5.03	2	5.00	3
50	5.47	2	5.37	3	5.47	3	5.87	5
75	6.73	8	7.13	10	6.87	10	6.47	6
100	7.60	12	6.67	8	6.83	9	6.73	7
150	6.80	9	6.63	7	6.87	10	6.97	8
200	6.00	5	7.10	9	6.73	8	7.63	11
250	6.70	7	5.60	4	5.90	4	5.67	4
300	6.23	6	7.23	11	6.37	6	7.33	9
500	7.20	11	6.43	5	6.30	5	7.37	10

750	6.87	10	6.60	6	6.40	7	4.60	2
1000	5.80	4	8.23	12	8.43	11	9.13	12

Table S3 Mean and final ranks obtained on the 10, 30, 50 and 100 dimensional CEC'17 problems using different execution rates

Execution Phase	10D		30D		50D		100D	
10	5.13	2	2.87	1	4.27	2	2.33	1
25	4.97	1	3.27	2	4.00	1	4.83	2
50	6.67	8	6.87	6	6.17	7	5.57	4
75	6.37	6	6.90	7	7.27	9	7.80	10
100	7.17	10	7.53	11	6.00	5	6.63	6
150	7.63	11	7.37	10	5.83	4	7.00	8
200	6.73	9	7.33	9	6.03	6	7.20	9
250	6.33	5	6.73	5	6.67	8	6.50	5
300	6.33	5	7.13	8	8.00	10	7.20	9
500	6.57	7	6.47	4	7.27	9	6.77	7
750	5.77	3	5.27	3	5.77	3	5.47	3
1000	6.13	4	8.07	12	8.53	11	8.50	11

Signal Range

The SR parameter range (SR_{min} ,SR_{max}ontrols the scope of influence the signaller particle has on the recipients. Hence, the value of SR could significantly alter the dynamics of the BEPSO algorithm. The mean and final ranks for the calibration of the SR parameter are shown in Table 1-5:8-6. In general, lower values of for the SR parameter tend to yield better results. For 10 and 50-dimensional CEC’14 functions, $SR_{max} = 25; SR_{min} = 25$ and for 30 and 100-dimensional CEC’14 functions $SR_{max} = 30; SR_{min} = 5$ exhibited the best performance. For the CEC’17 test functions, at 10 dimensions $SR_{max} = 600; SR_{min} = 60$, at 30 dimensions $SR_{max} = 100; SR_{min} = 100$, at 50 dimensions $SR_{max} = 100; SR_{min} = 10$ and at 100 dimensions $SR_{max} = 30; SR_{min} = 5$ attained the best performance. In addition, as shown in Figure 1-8, the results on the CEC’14 test functions reveal that $SR_{max} = 3; SR_{min} = 0.6$ consistently attained the lowest mean values for more functions compared to other SR values, and we particularly observe that the impact is more evident with increased dimensionality.

Table S4 Mean and final ranks obtained on the 10, 30, 50 and 100 dimensional CEC'14 problems using different signal range values

SR values	10D		30D		50D		100D	
$SR_{max} = 0.01; SR_{min} = 0.001$	4.23	6	3.63	2	3.30	1	3.30	1
$SR_{max} = 0.02; SR_{min} = 0.01$	4.23	6	3.83	4	3.97	4	4.33	6
$SR_{max} = 0.05; SR_{min} = 0.01$	3.53	2	4.00	5	3.83	3	3.67	2
$SR_{max} = 0.1; SR_{min} = 0.05$	3.23	1	4.00	5	4.37	7	3.77	3
$SR_{max} = 0.2; SR_{min} = 0.1$	4.03	4	3.77	3	4.03	5	4.00	5
$SR_{max} = 0.3; SR_{min} = 0.2$	4.07	5	4.63	6	4.10	6	3.90	4
$SR_{max} = 0.4; SR_{min} = 0.3$	3.97	3	3.43	1	3.70	2	4.33	6
$SR_{max} = 0.01; SR_{min} = 0.001$	4.23	6	3.63	2	3.30	1	3.30	1
$SR_{max} = 0.02; SR_{min} = 0.01$	4.23	6	3.83	4	3.97	4	4.33	6

$SR_{max} = 0.05; SR_{min} = 0.01$	3.53	2	4.00	5	3.83	3	3.67	2

Table S5 Mean and final ranks obtained on the 10, 30, 50 and 100 dimensional CEC'17 problems using different signal range values

SR values	10D		30D		50D		100D	
$SR_{max} = 0.01; SR_{min} = 0.001$	4.10	5	4.37	7	3.63	2	3.90	5
$SR_{max} = 0.02; SR_{min} = 0.01$	4.20	6	3.93	4	3.90	4	4.50	6
$SR_{max} = 0.05; SR_{min} = 0.01$	3.70	2	4.17	6	4.20	6	3.43	2
$SR_{max} = 0.1; SR_{min} = 0.05$	4.03	3	3.43	1	4.10	5	4.63	7
$SR_{max} = 0.2; SR_{min} = 0.1$	4.07	4	3.87	3	4.10	5	3.20	1
$SR_{max} = 0.3; SR_{min} = 0.2$	3.60	1	3.53	2	3.50	1	3.80	3
$SR_{max} = 0.4; SR_{min} = 0.3$	3.60	1	4.00	5	3.87	3	3.83	4
$SR_{max} = 0.01; SR_{min} = 0.001$	4.10	5	4.37	7	3.63	2	3.90	5
$SR_{max} = 0.02; SR_{min} = 0.01$	4.20	6	3.93	4	3.90	4	4.50	6
$SR_{max} = 0.05; SR_{min} = 0.01$	3.70	2	4.17	6	4.20	6	3.43	2

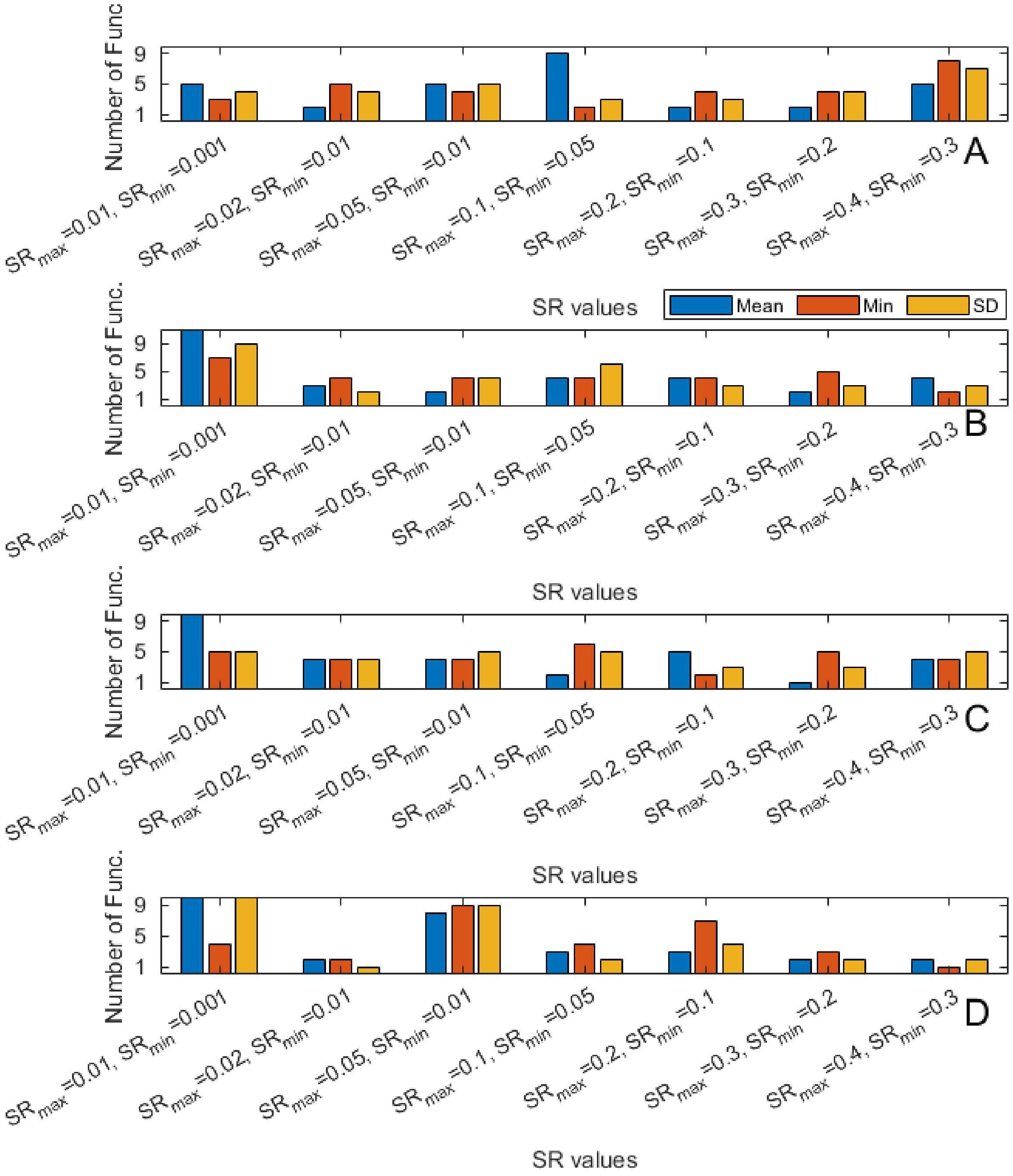

Figure S5 The impact of signal range on obtaining the lowest mean error, min, and standard deviation values on 10 (A), 30 (B), 50 (C) and 100 (D) dimensional CEC'14 problems

A single optimal SR value was not observed in our experiments however when we examine the results closely, we find that $SR_{max} = 0.01, SR_{min} = 0.001$ consistently exhibit the best performance for multimodal functions. For hybrid and composition functions, we observe three values $SR_{max} = 0.05, SR_{min} = 0.01$, $SR_{max} = 0.1, SR_{min} = 0.05$ and $SR_{max} = 0.2, SR_{min} = 0.1$ that dominates rest of the tested parameter values. Based on the outcome shown in Figure 1-8, $SR_{max} = 0.01, SR_{min} = 0.001$ will be used the main experiments.

Bias Threshold

In this subsection, the impact of the bias threshold parameter (BT) is assessed on the mean performance and convergence of the BEPSO algorithm. The bias threshold controls particles'

negative or positive *"perception"* towards other particles which in return influences the guidance of the particles. Hence, in essence, the higher values set for the bias threshold upper and lower limit tend to require more *evidence* for particles to be become positively or negatively biased, and on the contrary, the lower values force particles to rapidly attain a bias with minimal evidence.

Tables S7-S8 display the mean and final ranks obtained on 10, 30, 50 and 100-dimensional CEC'14 and CEC'17 benchmark problems for various bias threshold values. The ranks obtained suggest that in general, lower BT values (rapid bias) consistently yield better overall mean result across all dimensions of the CEC'14 and CEC'17 test suites. More specifically, for CEC'14 benchmark problems, $BT = 10 - 20$ and for CEC'17 problems, $BT = 20 - 25$ values exhibit the best mean performance. Hence, in the comparative experiments, $BT = 20$ is used for all test suites.

Table S6 Mean and final ranks obtained on the 10, 30, 50 and 100-dimensional CEC'14 problems using different (BT) bias threshold values

BT	10D		30D		50D		100D	
5	7.07	6	7.00	5	7.60	10	7.57	8
10	7.10	7	6.13	1	6.70	4	6.17	2
15	7.27	9	6.63	2	5.93	1	8.00	9
20	5.77	1	7.17	8	6.83	6	5.90	1
25	6.80	4	7.03	6	6.77	5	6.30	3
30	6.57	3	6.77	3	8.80	13	6.90	6
40	7.00	5	7.30	9	6.53	3	8.47	13
50	7.77	11	8.40	14	7.57	9	6.50	4
60	7.83	12	6.87	4	6.90	8	7.13	7
70	7.20	8	7.07	7	9.00	14	8.10	10
80	8.37	13	8.13	12	6.87	7	8.20	11
90	9.37	14	8.17	13	7.97	11	6.87	5
100	7.47	10	7.83	11	8.20	12	7.57	8
200	6.40	2	7.47	10	6.30	2	8.30	12

Table S7 Mean and final ranks obtained on the 10, 30, 50-and 100-dimensional CEC'17 problems using different (BT) bias threshold values

BT	10D		30D		50D		100D	
5	7.47	8	6.97	5	7.00	6	7.57	10
10	7.30	7	6.57	2	8.13	10	6.63	3
15	8.60	14	7.57	8	8.17	11	8.10	12
20	5.80	1	8.37	13	5.50	1	6.10	1
25	7.60	9	6.07	1	5.87	2	6.37	2
30	6.23	2	6.57	2	6.53	4	7.27	6
40	7.87	12	6.83	3	8.23	12	7.70	11
50	6.60	3	6.87	4	8.57	13	7.37	7
60	6.83	4	7.10	6	8.63	14	6.77	4
70	6.93	5	7.87	10	7.87	9	9.73	13
80	7.67	10	7.37	7	6.80	5	7.03	5
90	7.83	11	8.03	12	6.10	3	6.37	2

100	8.20	13	8.00	11	7.13	7	7.43	8
200	7.03	6	7.80	9	7.43	8	7.53	9

AHPSO Parameter Tuning and Analysis

AHPSO has a number of parameters, hence in this section, we assess the impact they have on the performance of the algorithm, testing on various dimensions and multiple test suites.

Alpha Parameter

The α parameter determines the size of the randomly selected number of potential lenders when a particle demands energy from other particles. Higher alpha value leads borrowing menial energy from each lender which precludes the expected imbalance in the current energy acquired by particles, which is required to create the behavioural heterogeneity (by triggering different behaviours) in the swarm. On the other hand, too low alpha value leads to large scale of energy borrowing from fewer particles. Once again, this procrastinates the required imbalance in the current energy levels which enforces the interaction between particles. Hence, in this section we aim to determine the optimal value for the alpha parameter that will enable the algorithm to maintain the aforementioned balance whilst maximising the overall performance. Initially, we conducted an experiment on the 10, 30, 50 and 100-dimensional CEC'13, CEC'14 and CEC'17 test functions and ranked the results based on the mean error values as shown in Table S9-S11.

According to the final ranks, we have identified five distinct values that consistently exhibited better performance compared to other values used for the α parameter. From Table 2-1:9-3, we observe that $\alpha = 14$ attained the best performance for 10-dimensional and 100-dimensional functions for CEC'13 and CEC'17 test suites, $\alpha = 12$ exhibited the top rank for 30-dimensional CEC'13 and 100-dimensional CEC'14 test suites and $\alpha = 16$ achieved the first rank on the 50-dimensional CEC'13 and 10-dimensional CEC'14 whilst $\alpha = 10$ exhibited superior mean performance for 50-dimensional CEC'14 and CEC'14 functions and finally, $\alpha = 18$ ranked first for 30-dimensional CEC'17 functions. The calibration of the α parameter reveals that the optimal values attained over three different dimensions and test suites spread across 10-18 hence, the optimal value is determined as $10 \leq \alpha \leq 18$. Hence, in the main experiments, the value of α is randomly determined between $10 - 18$ each time the parameter is used.

Table S8- Mean and final ranks of different α values on the 10, 30, 50 and 100 dimensional CEC'13 problems

d	10-d		30-d		50-d		100-d	
$\alpha = 4$	4.96	3	5.75	5	5.46	7	5.68	7
$\alpha = 6$	5.89	8	6.93	10	5.07	3	5.25	5
$\alpha = 8$	5.18	4	6.04	6	5.21	5	5.18	4
$\alpha = 10$	5.64	6	5.32	3	5.18	4	4.61	2
$\alpha = 12$	5.86	7	4.39	1	4.61	2	5.11	3
$\alpha = 14$	4.46	**1**	5.04	2	5.32	6	4.29	1
$\alpha = 16$	5.64	6	6.32	8	4.21	1	5.61	6
$\alpha = 18$	4.68	2	5.61	4	6.14	8	6.36	8
$\alpha = 20$	6.25	9	6.07	7	6.86	9	6.61	9
$\alpha = 25$	5.46	5	6.39	9	8.32	10	8.11	10

$\alpha = 30$	6.29	10	7.82	11	9.61	11	9.11	11

Table S9- Mean and final ranks of different α values on the 10, 30, 50 and 100 dimensional CEC'14 problems

d	10-d		30-d		50-d		100-d	
$\alpha = 6$	6.20	9	7.13	9	5.70	6	7.30	11
$\alpha = 8$	5.63	4	5.83	6	6.77	10	5.07	3
$\alpha = 10$	5.90	7	5.57	4	5.57	4	6.33	8
$\alpha = 12$	5.77	5	5.70	5	4.63	1	5.40	5
$\alpha = 14$	5.37	3	4.93	2	5.67	5	4.83	1
$\alpha = 16$	5.10	2	5.00	3	4.97	3	4.87	2
$\alpha = 18$	4.57	1	4.47	1	4.77	2	5.57	6
$\alpha = 20$	5.63	4	5.83	6	5.83	7	5.33	4
$\alpha = 25$	5.80	6	5.90	7	5.87	8	5.80	7
$\alpha = 30$	6.33	10	6.47	8	6.67	9	6.47	9
$\alpha = 4$	6.13	8	7.33	10	7.73	11	7.20	10

Table S10- Mean and final ranks of different α values on the 10, 30, 50 and 100 dimensional CEC'17 problems

d	10-d		30-d		50-d		100-d	
$\alpha = 4$	6.40	10	6.70	10	5.97	8	6.93	9
$\alpha = 6$	5.10	5	6.57	8	5.27	3	5.83	8
$\alpha = 8$	5.43	6	6.00	7	5.73	5	4.93	3
$\alpha = 10$	5.63	7	5.03	3	4.77	1	4.97	4
$\alpha = 12$	5.03	4	5.53	6	4.87	2	5.57	6
$\alpha = 14$	4.50	1	4.87	2	5.80	6	4.33	1
$\alpha = 16$	5.93	8	5.20	4	5.43	4	5.03	5
$\alpha = 18$	4.90	3	4.73	1	5.73	5	4.67	2
$\alpha = 20$	4.53	2	5.40	5	5.90	7	5.77	7
$\alpha = 25$	6.23	9	6.67	9	7.53	10	7.37	10
$\alpha = 30$	6.70	11	7.47	11	7.17	9	8.77	11

Energy Redistribution Rate

The impact and calibration of the ER parameter was assessed on the 10, 30, 50 and 100-dimensinoal CEC’13, CEC’14 and CEC’17 test functions. The performance of each parameter value is ranked based on the mean performance and ranks are shown in Tables S12-S14. The final ranks indicate that lower values consistently exhibit superior performance. ER=1 consistently exhibited the best mean performance for 10, 50 and 100-dimensional CEC’14 functions, in addition, $ER = 1$ provides the best performance for 10 and 100-dimensional CEC’17 test functions. The second-best value was observed as $ER = 5$ where superior performance is attained on the 30-dimensional CEC’14 and CEC’17 functions and for 50-dimensional CEC’17 functions. Besides $ER = 1$ and $ER = 5$, $ER = 10$ is the only value that achieved the best performance for 50-dimensional CEC’13 functions. Hence, $ER = 5$ and $ER = 1$ dominated and generally exhibited superior performance for the experiments conducted on 10, 30, 50 and 100-dimensions on CEC’13, CEC’14 and CEC’17 test suites.

Table S11 - Mean and final ranks of different ER values on the 10, 30, 50 and 100 dimensional CEC'13 problems

d	10-d		30-d		50-d		100-d	
$ER = 1$	6.89	5	5.04	2	5.54	2	3.64	2
$ER = 5$	4.86	1	4.07	1	5.86	3	3.25	1
$ER = 10$	5.61	2	5.32	3	3.68	1	4.93	4
$ER = 15$	8.57	14	5.64	4	6.93	5	4.54	3
$ER = 20$	8.14	10	7.46	5	6.39	4	6.29	5
$ER = 25$	6.96	6	7.75	6	7.29	6	6.89	6
$ER = 30$	6.71	3	8.21	7	8.57	9	8.64	8
$ER = 40$	7.93	8	9.00	9	8.21	8	9.39	9
$ER = 50$	6.82	4	9.36	12	7.86	7	8.50	7
$ER = 75$	7.43	7	8.89	8	10.00	14	10.54	12
$ER = 100$	8.68	15	9.18	11	9.21	10	9.43	10
$ER = 150$	8.07	9	10.68	14	11.32	15	10.32	11
$ER = 200$	8.50	13	10.68	14	9.68	11	10.61	14
$ER = 250$	8.43	12	9.57	13	9.75	13	10.57	13
$ER = 500$	8.29	11	9.14	10	9.71	12	10.86	15

Table S12- Mean and final ranks of different ER values on the 10, 30, 50 and 100 dimensional CEC'14 problems

d	10-d		30-d		50-d		100-d	
$ER = 1$	5.97	1	4.87	2	3.60	1	3.03	1
$ER = 5$	6.53	2	4.67	1	4.30	2	3.30	2
$ER = 10$	6.53	2	7.23	5	6.23	4	4.77	3
$ER = 15$	6.90	4	5.80	3	6.63	5	5.33	4
$ER = 20$	6.87	3	7.77	7	5.90	3	6.50	5
$ER = 25$	7.77	7	7.80	8	6.87	6	6.53	6
$ER = 30$	7.43	5	7.27	6	7.40	8	6.97	7
$ER = 40$	8.33	11	6.93	4	7.30	7	8.53	8
$ER = 50$	7.73	6	9.73	14	8.00	9	8.63	9
$ER = 75$	8.00	9	9.10	10	9.50	11	9.40	10
$ER = 100$	7.80	8	8.53	9	10.47	14	9.77	11
$ER = 150$	8.80	13	9.47	12	11.13	15	10.47	12
$ER = 200$	8.17	10	9.20	11	9.93	12	11.40	15
$ER = 250$	9.07	14	9.60	13	9.03	10	10.80	13
$ER = 500$	8.70	12	8.53	9	10.20	13	11.07	14

Table S13- Mean and final ranks of different ER values on the 10, 30, 50 and 100 dimensional CEC'17 problems

d	10-d		30-d		50-d		100-d	
$ER = 1$	5.67	1	4.50	2	4.53	2	3.07	1
$ER = 5$	6.93	5	3.97	1	4.10	1	3.47	2
$ER = 10$	6.10	2	6.13	3	5.30	3	3.60	3
$ER = 15$	8.40	13	6.87	5	5.87	4	5.10	4
$ER = 20$	8.20	11	6.50	4	6.23	5	7.03	6

$ER = 25$	7.63	6	8.13	7	7.50	6	6.80	5
$ER = 30$	8.47	14	8.33	8	8.27	9	7.30	7
$ER = 40$	6.73	4	8.93	10	7.73	7	8.33	8
$ER = 50$	8.27	12	9.07	12	8.17	8	8.77	9
$ER = 75$	8.07	9	7.60	6	9.10	10	10.67	13
$ER = 100$	8.63	15	9.63	14	10.70	15	10.73	14
$ER = 150$	6.47	3	9.63	14	9.77	13	10.37	12
$ER = 200$	7.87	8	9.00	11	10.07	14	11.63	15
$ER = 250$	7.83	7	8.83	9	9.47	11	10.13	11
$ER = 500$	8.17	10	9.37	13	9.70	12	9.50	10

Further assessment indicates that, as shown in Figures S6 – S7, among the sixteen different values, $ER = 1$ and $ER = 5$ yield significantly better performance in attaining the lowest mean, minimum and standard deviation values for majority of functions across different dimensions of multiple test suites. In addition, we observe that $ER = 1$ and $ER = 5$ exhibit better performance compared to other ER values tested for the majority of the unimodal, multimodal, hybrid and composition functions. Hence, considering the results presented in Figure S6, for the main experiments $ER = 1$ is used.

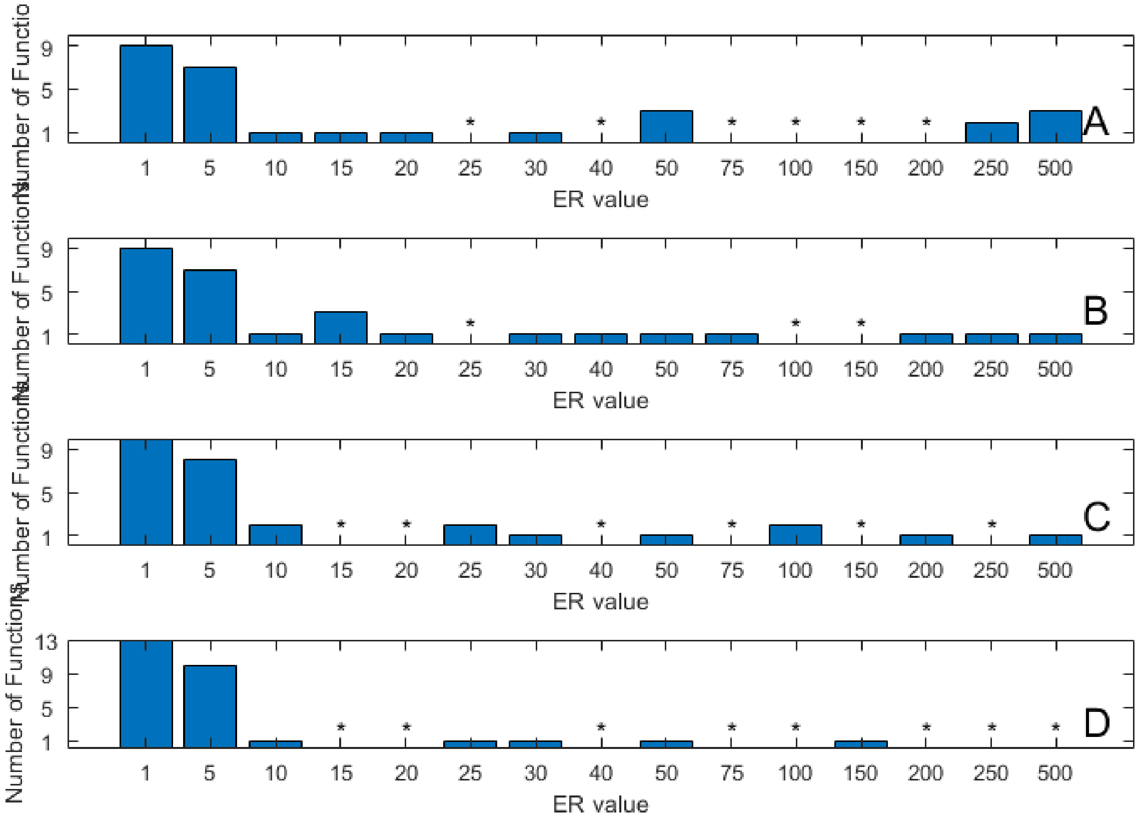

Figure S6-Total number of problems alpha values obtained the lowest mean error value on 10(A), 30(B), 50(C) and 100(D) dimensional CEC'13 problems. * no best solutions found.

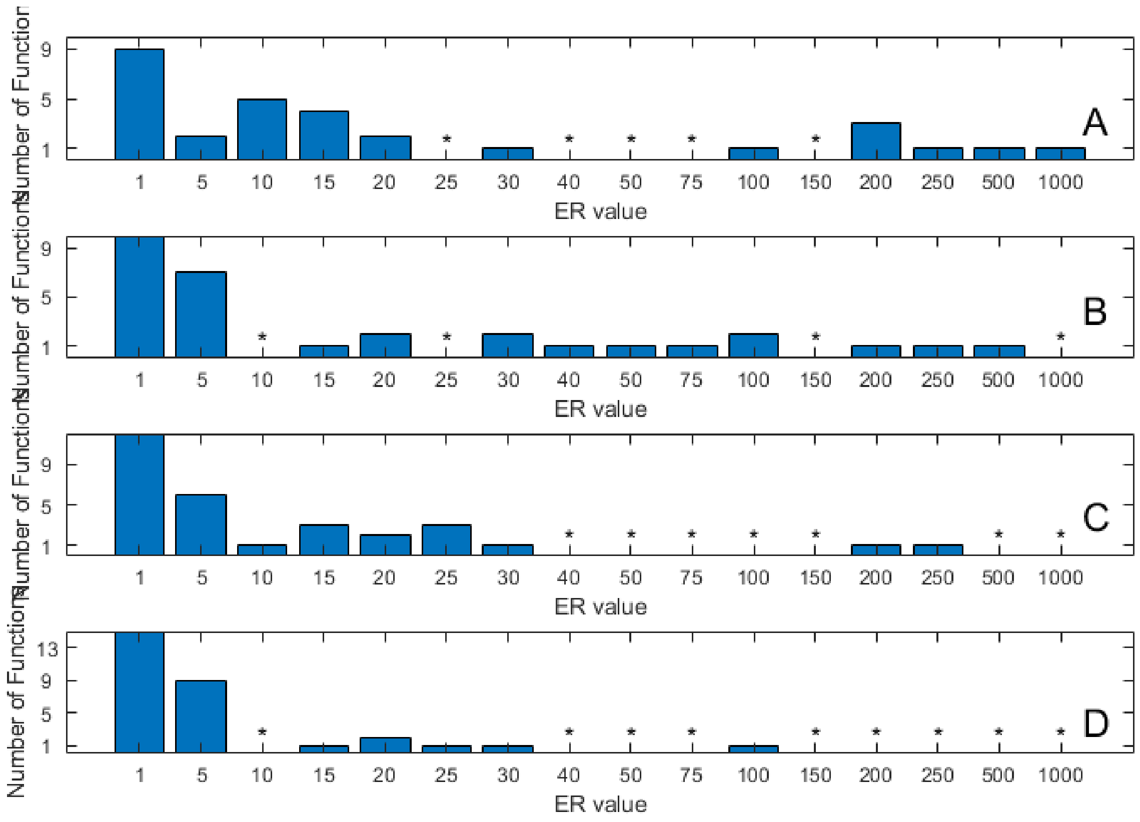

Figure S7-Total number of problems alpha values obtained the lowest mean error value on 10(A), 30(B), 50(C) and 100(D) dimensional CEC'14 problems. * no best solutions found

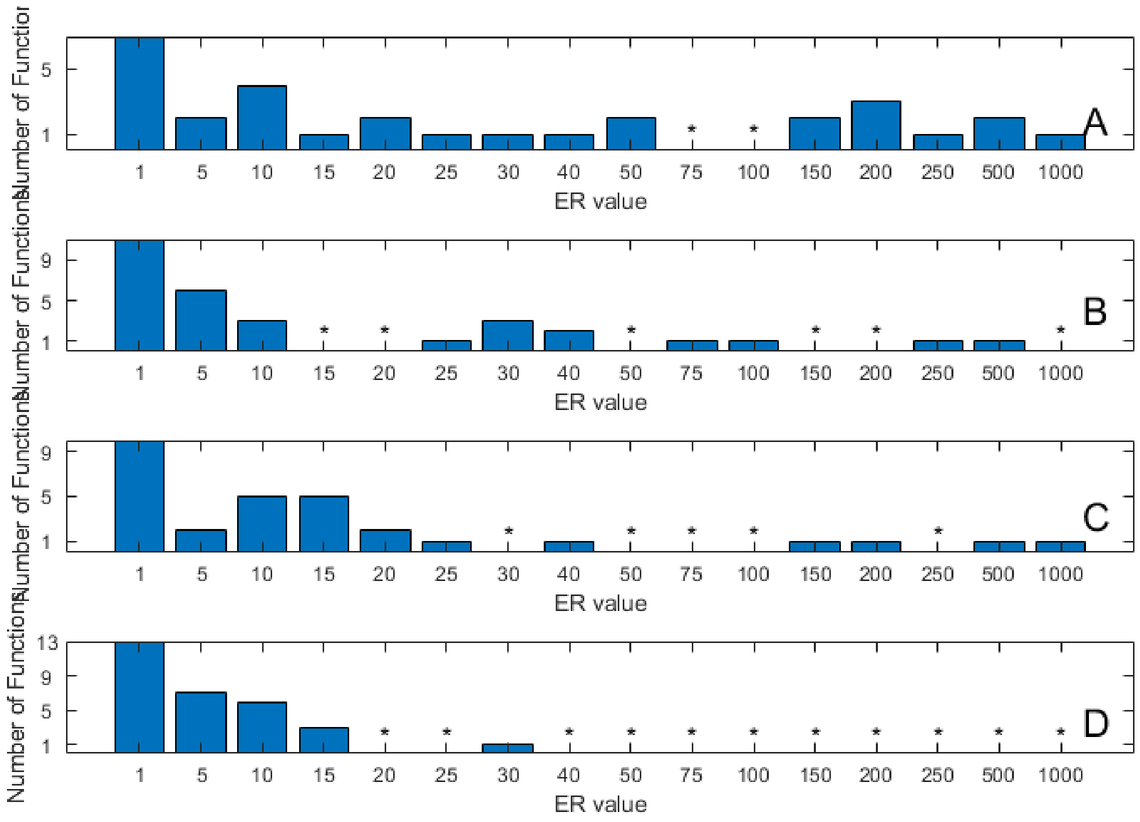

Figure S8-Total number of problems alpha values obtained the lowest mean error value on 10(A), 30(B), 50(C) and 100(D) dimensional CEC'17 problems. * no best solutions found

Lending-Borrowing Reset Rate

The lending-borrowing profile builds over time as particles move in the search space and interact with other particles. After a certain number of iterations, the gap between L and B can be irreversible which at higher level directly affects the value of β, a parameter that is used by the swarm and at an individual level. The mentioned gap causes repetitive rejection or approval (depending on which is higher) for energy borrowing requests of particles. Hence, the aforementioned issue is prevented by periodically resetting the values of L and B to maintain an approximate balance between them. The optimal interval to reset L and B is expected to be short but at the same time, it should provide sufficient time to allow particles to build a lending-borrowing profile. Hence to determine the optimal value, we conducted experiments on the 10, 30 and 50-dimensional CEC'13, CEC'14 and CEC'17 test suites using 17 different values as periods to reset the values of L and B. The obtained results are ranked according to their mean error values and are shown in Table 2-7:9-9. The final ranks indicate that for three test suites across four dimensions only $LB_{rate} = 5$ and $LB_{rate} = 10$ exhibit the best performances compared to rest of the values employed for the LB_{rate} parameter. $LB_{rate} = 5$ achieved the best performance for 50 and 100-dimensional CEC'13, 30, 50 and 100-dimensional CEC'14 and for all dimensions on the CEC'17 test suite. Moreover, $LB_{rate} = 10$ attained the 1st rank on the 10-dimensional CEC'13, CEC'14, and 30-dimensional CEC'13 test functions. Hence, we conclude that $LB_{rate} = 5$ is the optimal value on the three test suites employed and the second-best setting is $LB_{rate} = 10$.

Table S14- Mean and final ranks of different LB_{rate} values on the 10, 30, 50 and 100 dimensional CEC'13 problems

LB_{rate}	10-d		30-d		50-d		100-d	
5	5.36	2	5.07	2	3.25	1	2.82	1
10	4.89	1	5.04	1	4.18	2	4.32	2
15	7.86	6	6.07	3	6.32	3	5.25	3
20	6.43	3	6.25	4	7.18	5	6.75	6
25	9.14	12	6.86	5	6.39	4	6.25	4
30	8.93	11	8.25	7	8.07	6	6.50	5
35	6.79	4	8.36	8	8.21	7	9.29	8
40	8.86	10	7.86	6	9.00	9	8.14	7
45	7.50	5	8.82	9	10.36	12	9.54	9
50	9.18	13	9.68	10	11.61	14	10.18	11
60	7.89	7	10.89	12	10.29	11	10.11	10
70	8.39	8	10.61	11	8.61	8	10.43	12
80	8.50	9	11.36	13	11.14	13	11.54	13
100	10.57	15	11.96	16	9.57	10	12.07	15
150	11.25	17	11.79	14	12.00	15	11.93	14
200	11.21	16	12.25	17	13.21	16	13.89	17
250	10.39	14	11.89	15	13.61	17	13.79	16

Table S15-CEC'14- Mean and final ranks of different LB_{rate} values on the 10, 30, 50 and 100 dimensional CEC'14 problems

LB_{rate}	10-d		30-d		50-d		100-d	
5	5.37	2	4.73	1	2.93	1	2.93	1
10	4.00	1	5.00	2	4.30	2	3.93	2
15	7.80	3	5.67	3	6.27	3	4.87	3
20	8.07	5	6.97	4	6.27	3	6.17	4
25	8.90	9	8.27	6	7.40	5	7.47	5
30	7.90	4	8.43	8	6.73	4	8.07	7
35	8.63	7	9.03	9	9.17	7	7.57	6
40	9.93	12	9.43	11	10.17	10	8.10	8
45	8.43	6	9.23	10	9.33	8	9.57	10
50	9.43	10	8.33	7	10.07	9	8.83	9
60	10.17	13	7.97	5	9.10	6	9.80	11
70	9.93	12	10.50	15	10.70	13	10.90	13
80	8.87	8	9.90	13	10.20	11	10.37	12
100	9.60	11	9.63	12	12.00	16	11.03	14
150	10.53	14	10.47	14	10.43	12	12.73	15
200	9.60	11	12.80	17	11.50	14	13.13	17
250	10.53	14	12.10	16	11.90	15	13.00	16

Table S16-CEC'17- Mean and final ranks of different LB_{rate} values on the 10, 30, 50 and 100 dimensional CEC'17 problems

LB_{rate}	10-d		30-d		50-d		100-d	
5	4.93	1	4.03	1	3.10	1	2.30	1
10	6.10	3	4.17	2	4.43	2	2.83	2
15	6.80	5	4.90	3	5.80	3	4.37	3
20	8.33	8	6.50	4	6.40	4	4.43	4
25	8.03	6	7.43	7	6.87	6	6.27	5
30	6.50	4	7.10	6	6.57	5	8.07	7
35	10.50	13	8.97	8	7.70	7	8.43	8
40	8.30	7	6.80	5	8.97	8	8.67	9
45	9.67	11	9.23	9	9.80	11	7.93	6
50	5.60	2	10.43	11	9.20	9	10.03	10
60	9.87	12	10.47	12	9.77	10	10.87	12
70	8.73	9	9.83	10	9.80	11	10.23	11
80	9.63	10	11.37	15	10.67	12	11.80	13
100	10.87	14	11.33	14	11.83	14	12.00	14
150	11.00	15	11.20	13	12.80	15	12.87	15
200	9.87	12	11.87	16	11.40	13	13.60	16
250	11.63	16	12.83	17	13.37	16	13.77	17

We further assess the performance of the LB_{rate} parameter on the mean, minimum and standard deviation values. Tables S15 – S17 exhibits the parameter values tested for the LB_{rate} and the comparison of the total number of functions each value attained the lowest f_{mean}, $f_{\min}$ and the standard deviation on different dimensions for CEC'13, CEC'14 and CEC'17 test functions. As shown in Tables S15-S17, we also observe improvements in the performance of AHPSO with increased dimensionality when $LB_{rate} = 5$, which indicates that the LB_{rate} parameter is more influential and contributes effectively to the search process in higher dimensional problems. Hence, for the main experiments conducted, $LB_{rate} = 5$ is used.

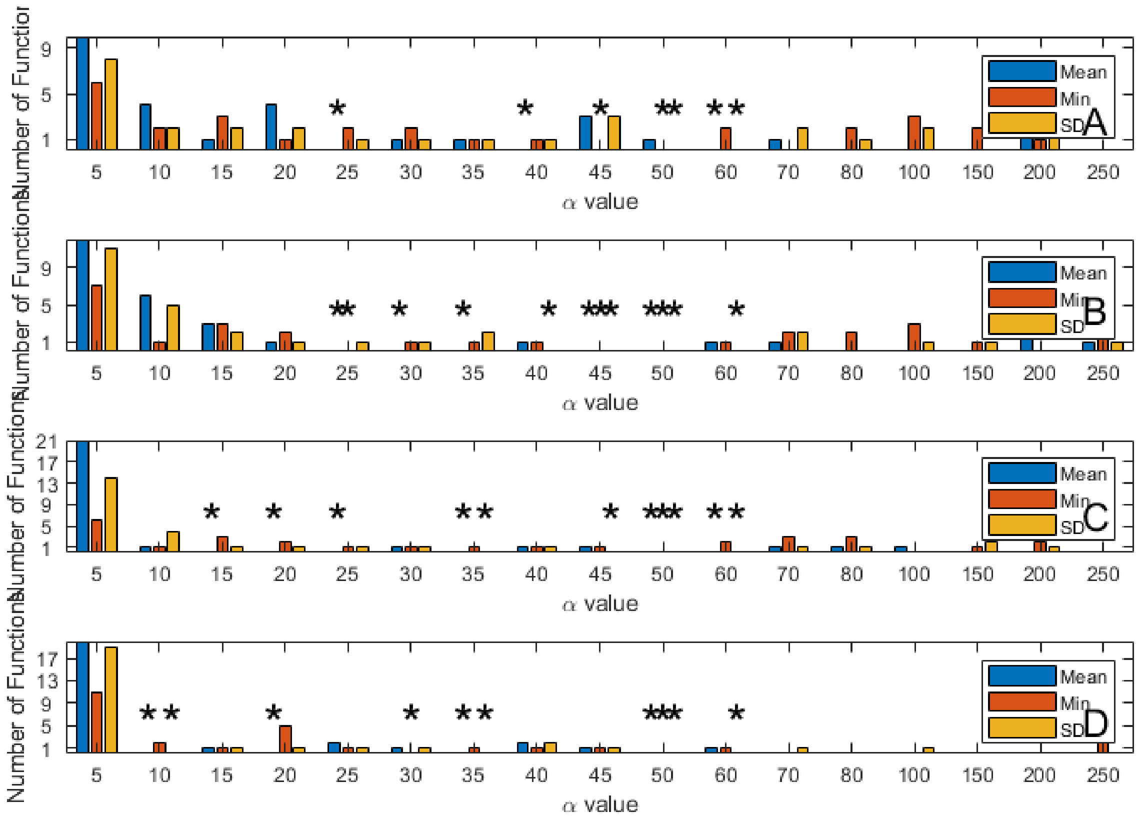

Figure S9-Total number of problems LB_{rate} values obtained the lowest mean error value on 10(A), 30(B), 50(C) and 100(D) dimensional CEC'13 problems. * no best solutions found.

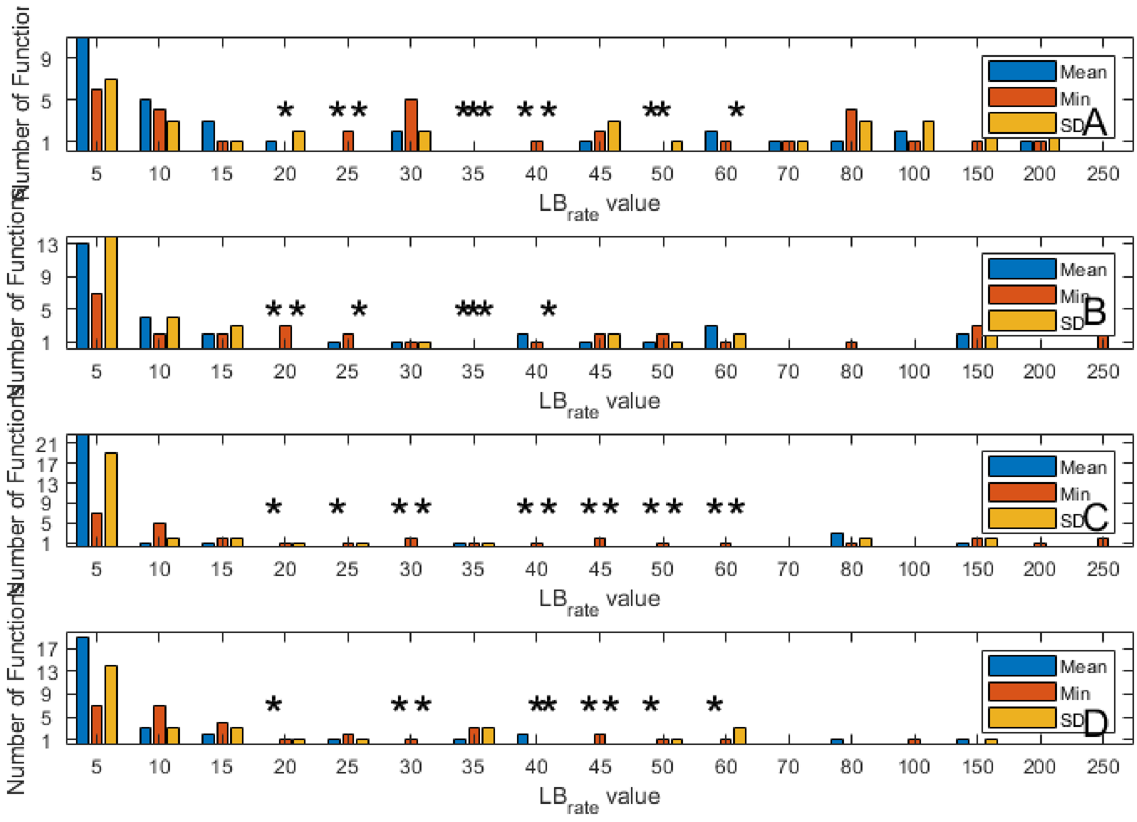

Figure S10- Total number of problems LB_{rate} values obtained the lowest mean error value on 10(A), 30(B), 50(C) and 100(D) dimensional CEC'14 problems. * no best solutions found.

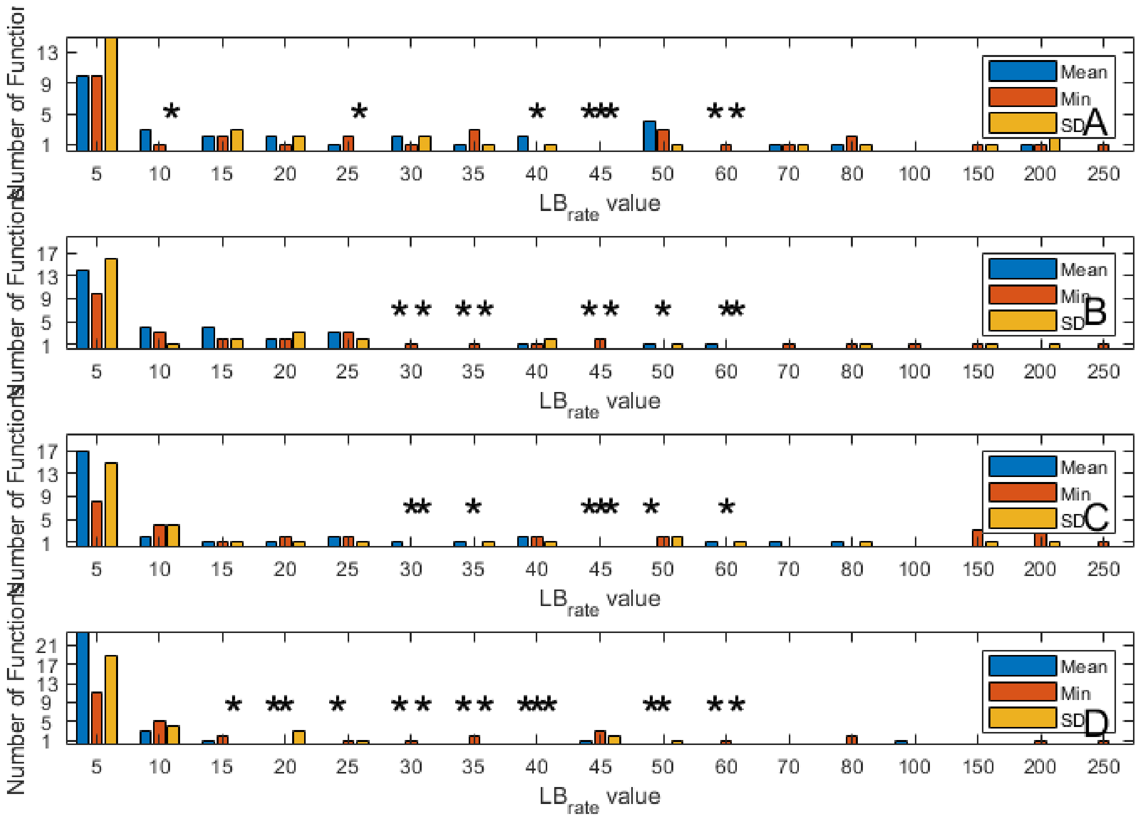

Figure S11- Total number of problems LB_{rate} values obtained the lowest mean error value on 10(A), 30(B), 50(C) and 100(D) dimensional CEC'17 problems. * no best solutions found.

Population Size

To assess the impact of population size on the performance of AHPSO, we have conducted an experiment on the 10, 30, 50 and 100-dimensional CEC’13, CEC’14 and CEC’17 test functions using various population sizes and observed the impact on the overall mean performance. The obtained mean error results across three test suites are ranked and are shown in Tables S18-S20. Our findings indicate that for 30, 50 and 100-dimensional functions on the three test suites, the population size of 60 (n=60) consistently exhibit better performance compared to the rest of the values tested for particle population. On the other hand, for 10-dimensional functions, we generally observe that denser population (e.g., n=80, n=100, n=200) exhibit better performance on the CEC’13, CEC’14 and CEC’17 test functions. However, since n=60 exhibited better mean performance on all test suites and across most dimensions, hence in the main experiments, $n = 60$ will be used.

Table S17-Calibration of population size on the 10, 30, 50 and 100 dimensional CECE’13 problems

n	10-d		30-d		50-d		100-d	
10	11.21	12	12.61	13	12.32	12	12.68	13
15	9.96	11	10.86	12	10.86	11	10.89	12
20	8.86	10	9.61	11	9.54	10	9.93	11
30	6.39	9	7.68	10	7.32	9	8.32	10
35	5.75	8	5.68	5	6.18	7	6.50	9
40	5.07	2	4.79	2	5.71	5	5.89	8
45	5.43	5	5.89	6	5.36	3	5.50	5

50	5.07	2	5.39	3	4.96	2	5.61	6
60	5.21	3	4.68	1	4.71	1	4.50	1
70	5.71	7	5.93	7	5.79	6	4.71	2
80	5.29	4	6.32	9	5.64	4	5.25	4
100	4.75	1	5.54	4	5.79	6	5.11	3
200	5.54	6	6.04	8	6.82	8	5.75	7

Table S18- Calibration of population size on the 10, 30, 50 and 100 dimensional CECE'14 problems

n	10-d		30-d		50-d		100-d	
10	11.27	13	11.83	12	12.13	12	12.47	12
15	9.73	12	10.97	11	10.17	11	10.83	11
20	9.40	11	9.23	10	8.20	10	9.67	10
30	6.70	9	6.67	9	6.50	8	8.07	9
35	6.73	10	6.27	8	6.10	6	6.97	8
40	6.20	8	5.63	5	6.00	5	5.70	7
45	5.83	6	5.67	6	5.70	4	5.27	6
50	5.87	7	5.67	6	4.77	2	5.23	5
60	4.73	2	4.67	1	4.73	1	4.60	1
70	5.20	4	4.93	2	5.03	3	4.63	2
80	4.63	1	5.13	3	6.40	7	4.73	3
100	4.93	3	5.57	4	6.00	5	5.00	4
200	5.27	5	6.17	7	6.67	9	5.23	5

Table S19- Calibration of population size on the 10, 30, 50 and 100 dimensional CECE'17 problems

n	10-d		30-d		50-d		100-d	
10	10.87	13	12.17	13	12.27	12	12.33	13
15	10.30	12	10.23	12	10.63	11	11.10	12
20	7.80	11	9.13	11	9.17	10	9.67	11
30	6.73	10	7.17	10	6.20	8	7.20	10
35	6.67	9	6.80	9	5.83	5	6.23	8
40	6.23	8	6.30	7	6.20	8	4.50	3
45	5.70	7	5.23	4	5.60	4	5.17	5
50	5.47	6	5.97	6	5.00	3	5.00	4
60	5.07	3	3.37	1	4.03	1	4.23	1
70	5.20	5	4.77	2	4.97	2	4.47	2
80	4.57	2	4.87	3	5.93	6	5.80	6
100	5.17	4	5.83	5	6.13	7	5.90	7
200	4.37	1	6.57	8	6.43	9	6.80	9

Paired Particle Mechanism (PPM) and Paired Population Size

The paired particle model proposed in the AHPSO algorithm aimed to delay the loss of population diversity to assist the AHPSO algorithm in prevention of premature convergence. In this section, we assess the impact of the PPM on the population diversity and the overall mean performance on the 30, 50 and 100-dimensional CEC'13, CEC'14 and CEC'17 test suites with

the PPM mechanism enabled and disabled. Table S21 display the average and final ranks for the mean performances of AHPSO algorithm with and without the PPM mechanism. The mean and final ranks reveal that AHPSO with the PPM mechanism achieved better ranks compared to the version without the PPM on the 30,50 and 100-dimensional CEC'13, CEC'14 and CEC'17 test suites. We also observe from Table 2-13 that the PPM mechanism is more effective on the higher dimensional functions in all three test suites.

Table S20- Mean ranks with the PPM mechanism enabled and disabled on the 10, 30, 50 and 100 dimensional CEC'13, CEC'14 and CEC'17 problems

	CEC'13		CEC'14		CEC'17	
	PPM on	PPM off	PPM on	PPM off	PPM on	PPM off
10D	1.60	1.39	1.53	1.43	1.70	1.26
30D	1.42	1.72	1.33	1.63	1.36	1.60
50D	1.31	1.87	1.30	1.76	1.33	1.73
100D	1.26	1.79	1.23	1.73	1.26	1.80

In addition, as shown in Figure S12, the population diversity comparison reveals that the AHPSO algorithm with PPM successfully maintains significantly better population diversity for 30, 50 and 100-dimensional functions. Moreover, Figure S13 shows the convergence rates for the same problem set and it is worth noting that AHPSO with PPM converged to a better or similar mean solution in most cases whilst maintaining a more diverse population. Moreover, Figure S14-S16 displays the total number of functions where each version of AHPSO (with and without PPM) attained the best mean error values over 30 consecutive runs, once again, AHPSO with PPM obtains better mean error values for higher dimensional (50-d and 100-d) functions. The results shown in Table S22 and Figure S17 corroborate the impact of the PPM mechanism and we clearly observe the true impact of the PPM mechanism with increased dimensionality on the overall performance of the AHPSO algorithm.

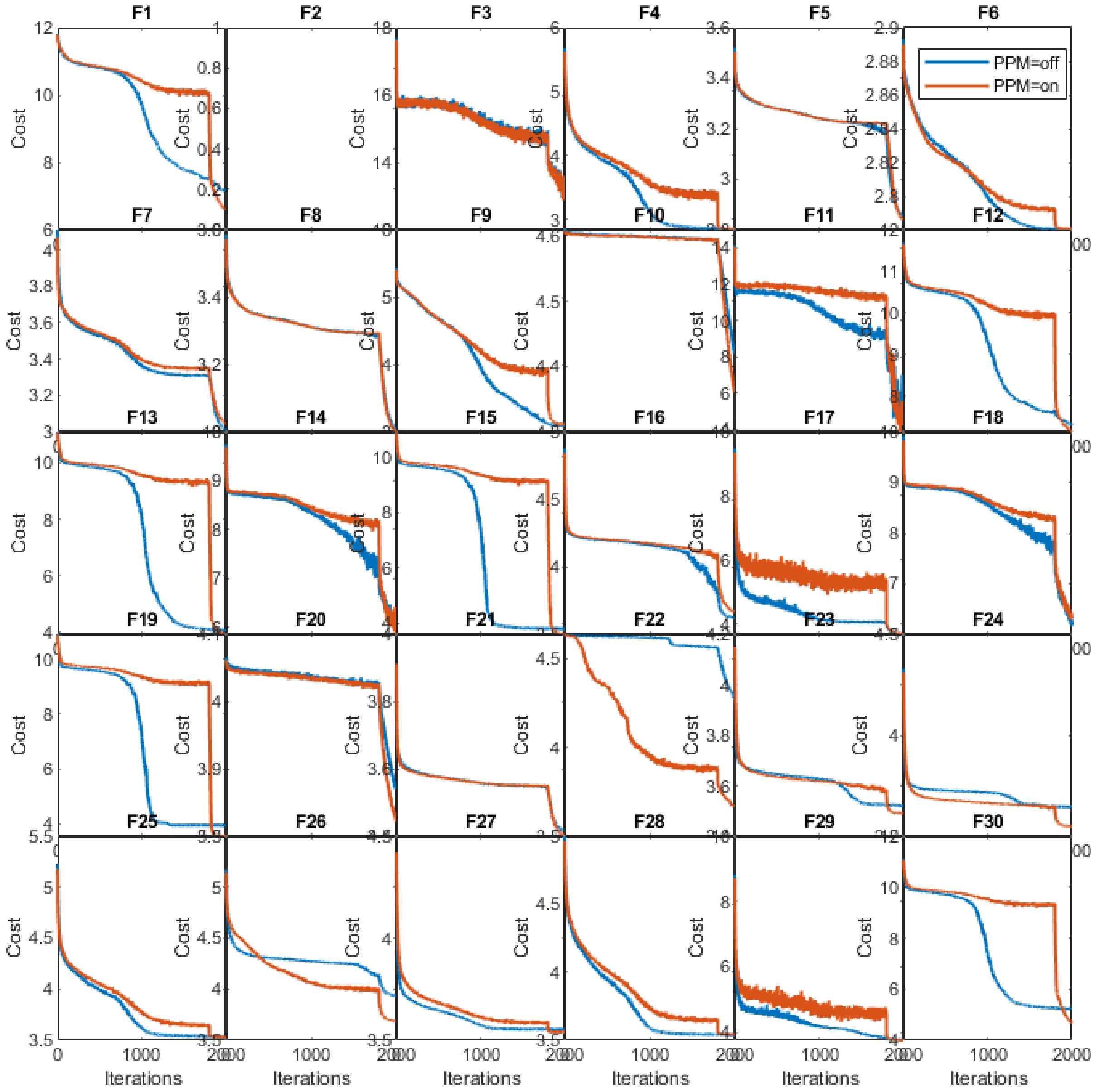

Figure S12- Population diversity comparison of AHPSO with PPM enabled and disabled on the 100 dimensional CEC'17 problems

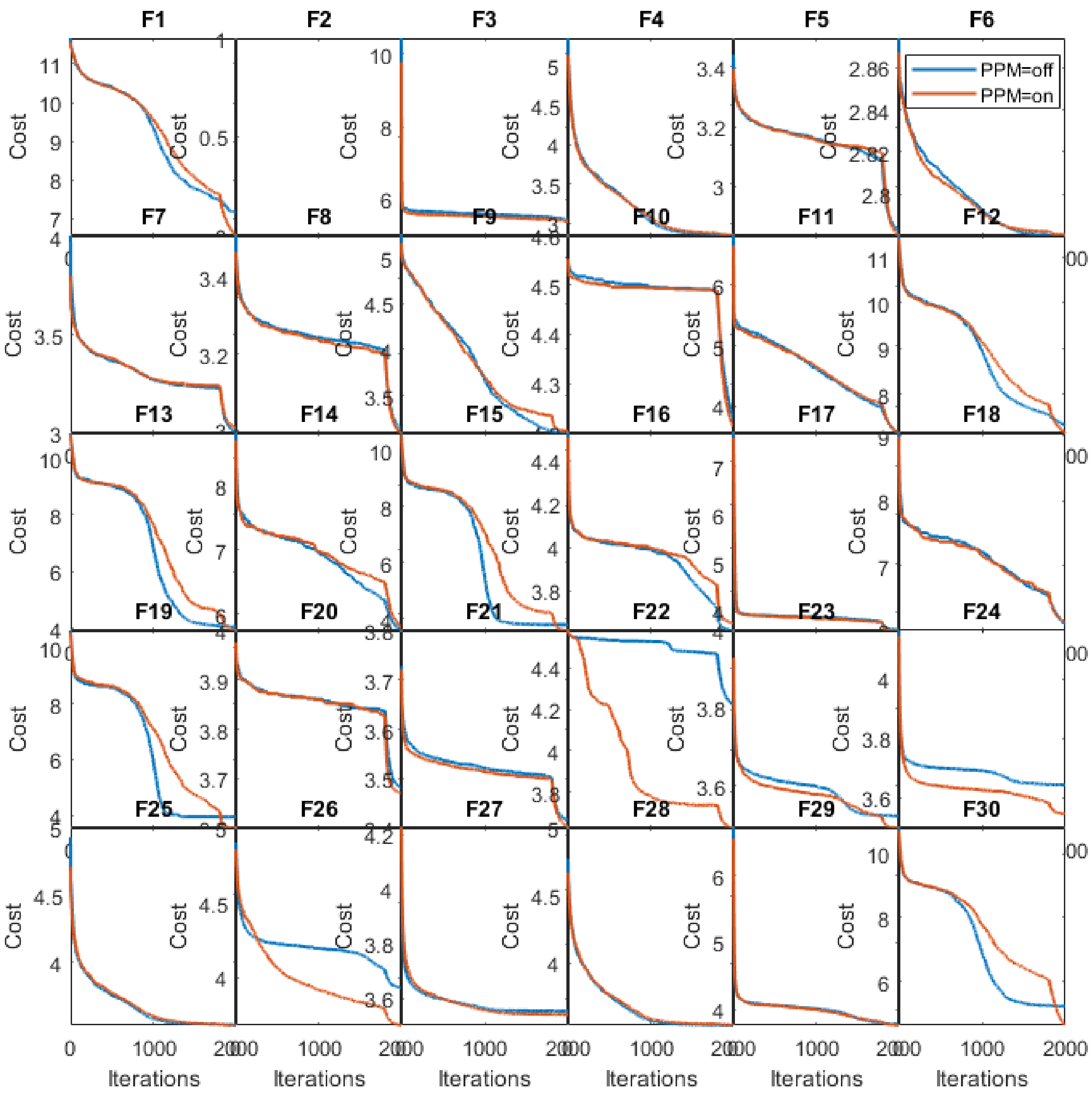

Figure S13- Convergence rate comparison of AHPSO with PPM enabled and disabled on the 100 dimensional CEC'17 problems

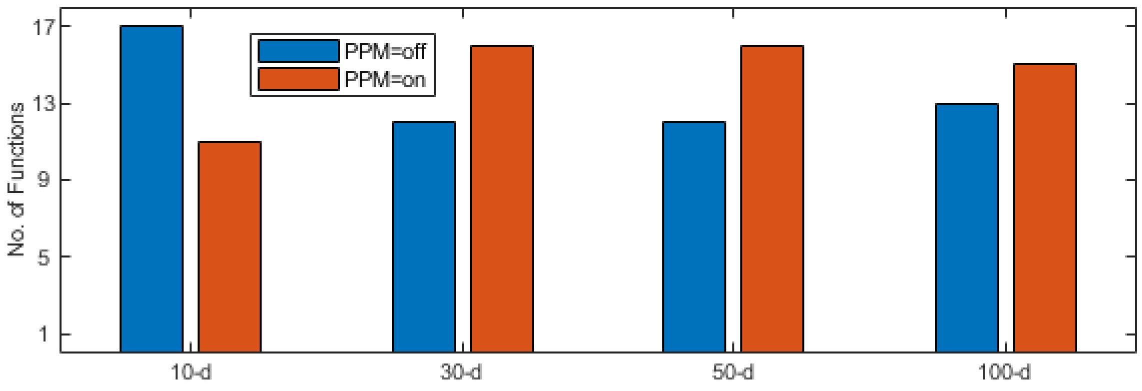

Figure S14- The total number of problems where AHPSO with and without the PPM exhibited the best performance on the 10, 30, 50 and 100-dimensional CEC’13 problems.

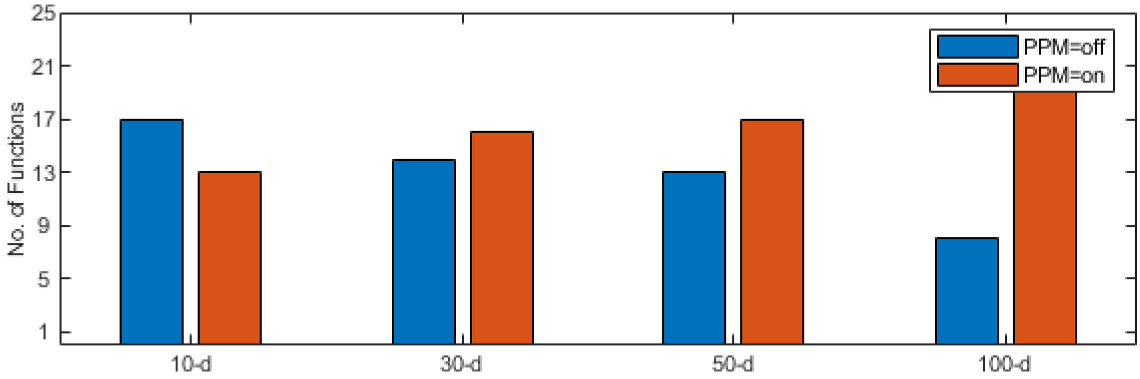

Figure S15- The total number of problems where AHPSO with and without the PPM exhibited the best performance on the 10, 30, 50 and 100-dimensional CEC'14 problems.

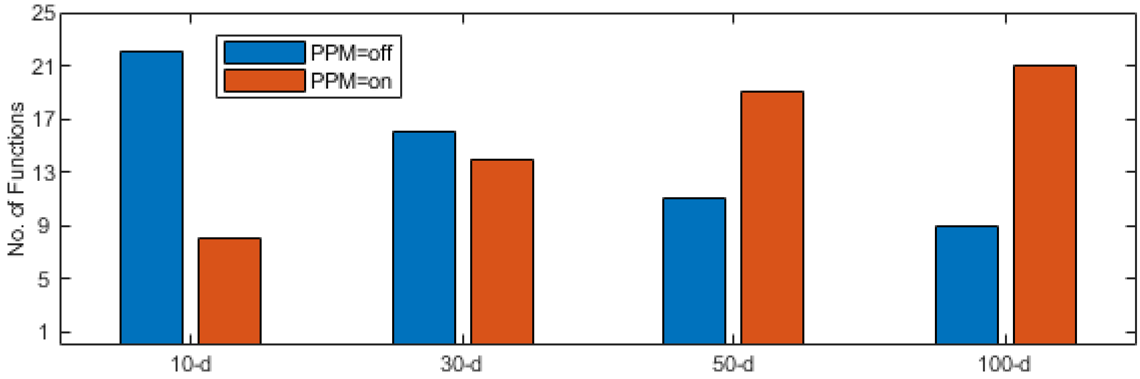

Figure S16- The total number of problems where AHPSO with and without the PPM exhibited the best performance on the 10, 30, 50 and 100-dimensional CEC'17 problems

After verifying the general impact of the PPM mechanism, we conducted a series of experiments to determine the optimal paired particle population that can reduce or increase the impact of the PPM mechanism. Hence, using 11 different paired particle population sizes, we have conducted experiments on the 10, 30, 50 and 100-dimensional CEC'17 test functions. Table S22 displays the mean and final ranks on the obtained mean error values for different paired particle population sizes for 10, 30, 50 and 100-dimensional CEC'17 functions. The results indicate that for 10-dimensional functions $n_{paired} = 16$ performed best, subsequently, for 30, 50 and 100-dimensional functions, $n_{paired} = 10$, $n_{paired} = 30$ and $n_{paired} = 20$ exhibited superior performance compared to the rest of the values tested. The second-best value was observed to be $n_{paired} = 6$ for 10 and 30-dimensional functions. $n_{paired} = 6$ was also observed to exhibit fair performance on the 50 and 100-dimensional functions.

Figure S17 exhibits the total number of functions where each n_{paired} value attained the lowest mean error. Although in Figure S17, we observe that $n_{paired} = 6$ consistently enabled AHPSO to attain lower mean errors for more functions compared to rest of the n_{paired} values employed, however as shown in Table S22, the ranks portray slightly different results. The convergence rates shown in Figure S18 indicate that the lower the n_{paired} value, the faster convergence is attained as PPM aims to delay the population diversity, hence in some cases, the denser paired particle population tend to cause slightly slower convergence however the final converged solution is hardly any different. Although the best mean performance is attained by different n_{paired} values at different dimensions (with slight mean difference). We observe compelling evidence that lower n_{paired} values favour convergence and appear as a more suitable fixed

general setting. Hence, considering the results exhibited in Figure S17 and Figure S18, in the main experiments $n_{paired} = 6$ will be used.

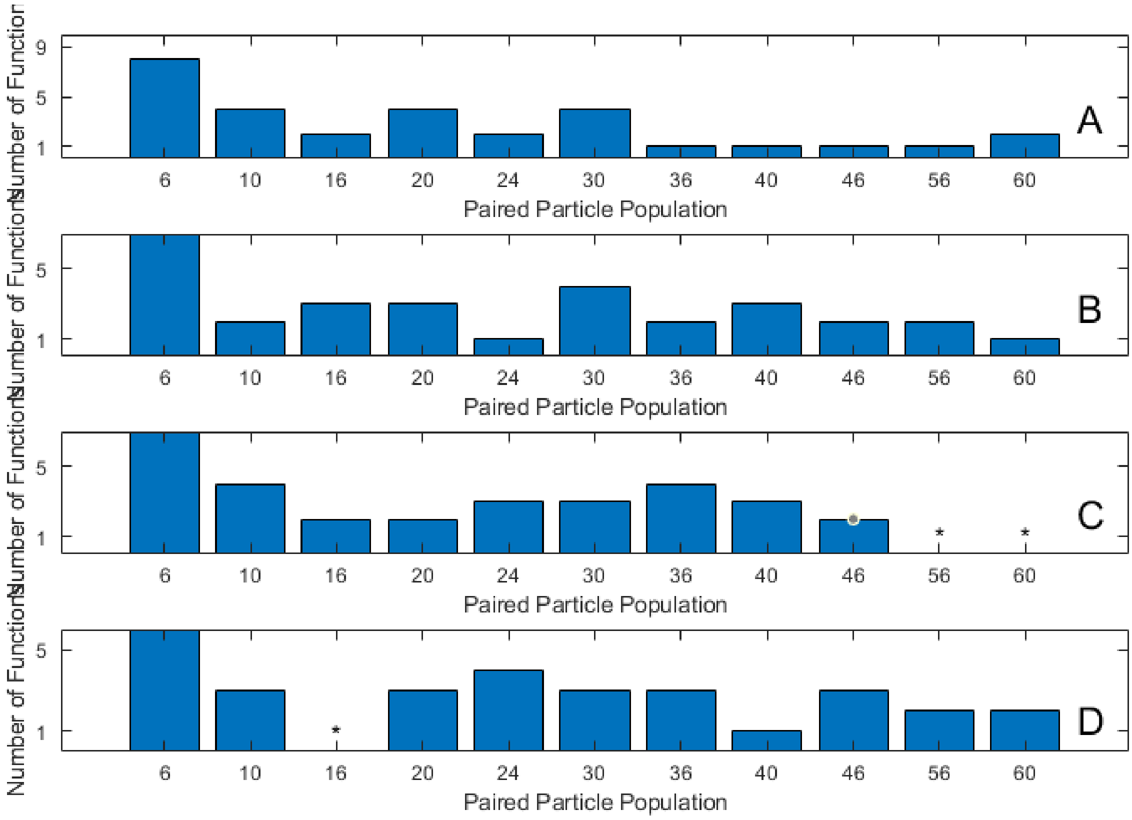

Figure S17- The impact of different paired population sizes on the number of best performances obtained by AHPSO for 10(A), 30(B), 50(C) and 100(D) dimensional CEC’17 problems

Table S21-Mean and final ranks obtained using different paired particle population sizes on the 10, 30, 50 and 100 dimensional CEC'17 problems

n_{paired}	10D		30D		50D		100D	
6	4.57	2	4.90	2	5.57	4	5.37	3
10	5.00	4	4.73	1	5.53	3	5.37	3
16	4.53	1	5.27	3	5.63	5	5.57	6
20	5.27	5	6.17	8	4.97	2	4.50	1
24	5.37	6	5.40	5	5.97	7	5.50	4
30	4.97	3	5.37	4	4.90	1	5.30	2
36	5.87	7	6.60	9	5.57	4	5.53	5
40	6.00	8	5.60	6	6.03	8	6.10	7
46	6.23	9	6.10	7	5.83	6	6.57	8
56	7.67	10	7.07	11	7.03	9	7.30	10
60	7.90	11	6.97	10	7.13	10	7.07	9

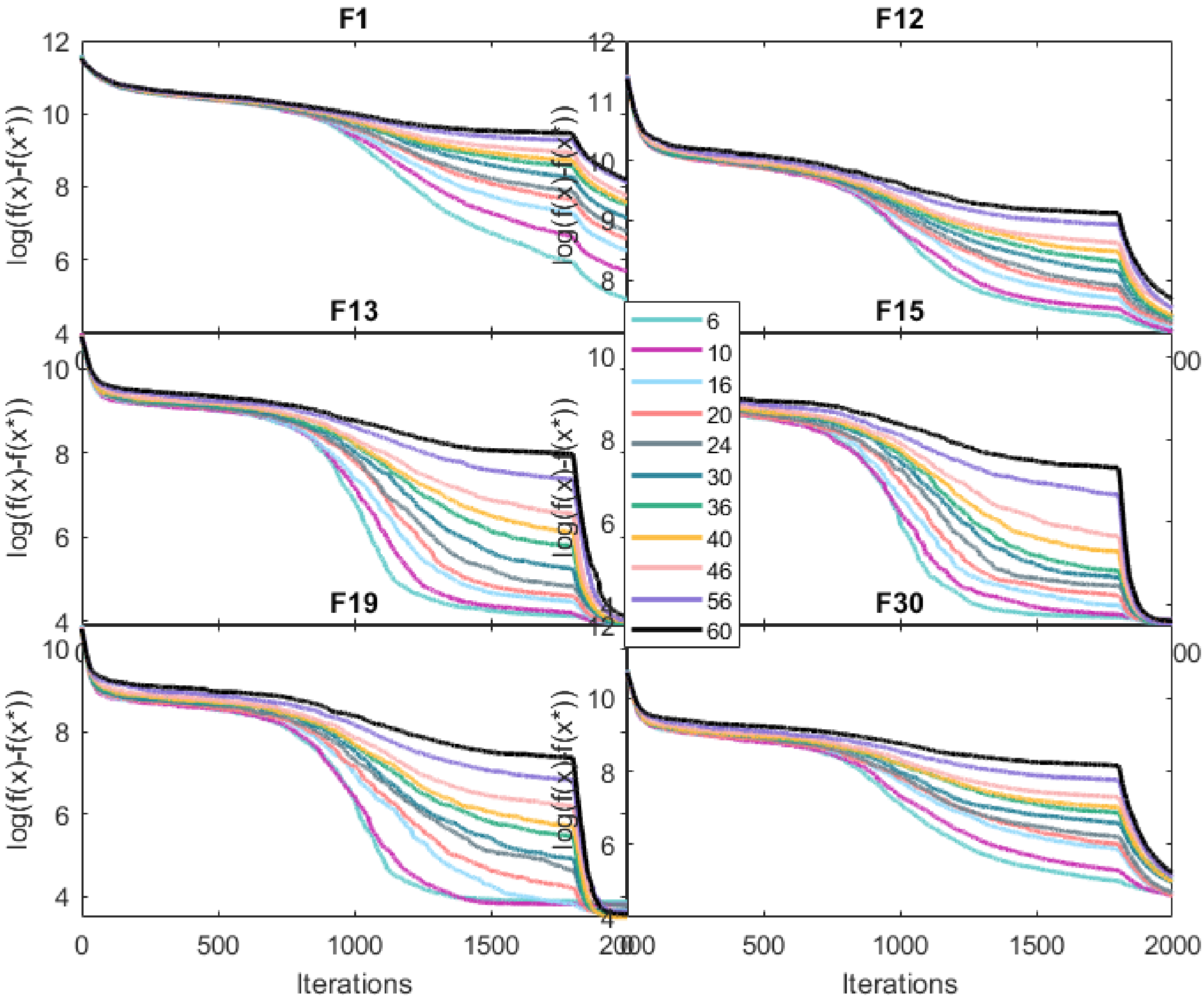

Figure S18-The convergence rate comparison of AHPSO with different population sizes on various 100 dimensional (CEC'17) unimodal, multimodal, hybrid and composition functions.

Beta Parameter

During search, the beta parameter is self-regulated, and its value is always equal to the average altruism value of particles. Beta is employed as a threshold value/criterion to initiate the energy sharing process hence has significant influence on the type of velocity update equation particles adopt. In summary, the beta parameter directly impacts the heterogeneity properties of the AHPSO algorithm. Although the AHPSO algorithm exhibits sufficient performance with the self-regulated version of the beta parameter, in this section we assess the precise impact of beta on the overall performance. In addition, we observe the impact of the balance of the heterogeneity in the swarm during search and the rate of convergence. We have used 12 different values for the beta parameter on the 10,30,50 and 100-dimensional CEC'13, CEC'14 and CEC'17 test functions. The results are ranked according to mean error values and are shown in Tables S23-S25. Overall, the top ranks obtained on the CEC'13 test functions is spread across different beta values however we observe a more stable pattern on the CEC'14 and CEC'17 test suites, as $\beta = 0.7 - 0.8$ exhibit the best performance.

Table S22- Mean and final ranks obtained using different β values on the 10, 30, 50 and 100 dimensional CEC'13 problems

β	10-d	30-d	50-d	100-d

$\beta = 0.05$	6.75	11	7.18	8	6.25	2	6.57	8
$\beta = 0.1$	6.04	8	6.36	5	6.32	4	6.50	7
$\beta = 0.15$	4.89	1	6.75	7	6.89	7	6.50	7
$\beta = 0.2$	5.71	5	6.21	3	6.32	4	5.68	1
$\beta = 0.25$	6.11	9	6.04	2	6.21	1	6.18	4
$\beta = 0.3$	5.86	6	6.36	5	6.46	5	7.00	10
$\beta = 0.4$	6.79	12	7.25	9	6.32	4	6.75	9
$\beta = 0.5$	5.04	2	6.46	6	6.29	3	6.46	6
$\beta = 0.6$	6.00	7	7.25	9	6.71	6	5.93	2
$\beta = 0.7$	6.54	10	6.32	4	7.00	9	6.14	3
$\beta = 0.8$	5.36	4	7.18	8	6.96	8	7.32	11
$\beta = 0.9$	5.25	3	4.64	1	6.25	2	6.43	5

Table S23- Mean and final ranks obtained using different β values on the 10, 30, 50 and 100 dimensional CEC'14 problems

β	10-d		30-d		50-d		100-d	
$\beta = 0.05$	6.97	9	7.23	8	8.47	10	9.00	12
$\beta = 0.1$	7.00	10	7.97	10	8.57	11	8.37	11
$\beta = 0.15$	6.87	8	7.97	10	8.30	9	7.53	10
$\beta = 0.2$	6.30	6	7.80	9	7.37	8	7.03	8
$\beta = 0.25$	6.63	7	7.97	10	7.37	8	7.40	9
$\beta = 0.3$	7.33	11	6.87	7	7.13	7	6.60	7
$\beta = 0.4$	7.53	12	6.43	6	5.37	5	5.97	6
$\beta = 0.5$	5.43	5	4.97	5	5.73	6	5.83	5
$\beta = 0.6$	5.27	3	4.73	3	4.60	3	4.43	3
$\beta = 0.7$	4.67	1	4.47	1	4.23	2	4.13	1
$\beta = 0.8$	5.00	2	4.63	2	3.97	1	4.30	2
$\beta = 0.9$	5.40	4	4.77	4	4.70	4	5.20	4

Table S24- Mean and final ranks obtained using different β values on the 10, 30, 50 and 100 dimensional CEC'17 problems

β	10-d		30-d		50-d		100-d	
$\beta = 0.05$	7.73	10	8.10	9	7.73	9	9.07	11
$\beta = 0.1$	7.50	9	8.00	8	8.20	11	8.97	9
$\beta = 0.15$	8.00	11	8.40	10	8.10	10	9.40	12
$\beta = 0.2$	7.00	7	8.47	11	8.40	12	9.03	10
$\beta = 0.25$	7.00	7	6.90	6	7.23	7	7.30	7
$\beta = 0.3$	7.37	8	7.07	7	7.47	8	7.53	8
$\beta = 0.4$	5.20	5	5.00	4	6.00	5	6.30	6
$\beta = 0.5$	5.27	6	6.03	5	6.10	6	4.57	5
$\beta = 0.6$	4.83	4	4.40	2	4.53	3	3.97	4
$\beta = 0.7$	4.20	2	4.53	3	4.37	2	3.30	2
$\beta = 0.8$	4.10	1	4.37	1	3.10	1	3.33	3
$\beta = 0.9$	4.67	3	4.53	3	4.57	4	3.03	1

The heterogeneity in the AHPSO algorithm is attained by the four velocity update equations denoted as $v_1 \dots v_4$ which allow particles to have distinct influences whilst moving in the search space. As previously mentioned, the beta parameter directly impacts the balance of the velocity update equation adopted by particles. The minimum percentage of adoption for each velocity update equation is expected to be 20% and the maximum is 35%, approximately during the entire search process. The stated minimum and maximum values allow each velocity update equation to be employed sufficiently to enable the required heterogeneity and maximise the performance of the algorithm. Figure S19 exhibits how the heterogeneity of the AHPSO algorithm is affected by different beta values. We can clearly observe from Figure S19 that the values between 0.05 and 0.6 do not yield the expected balance between the velocity update equations and in all cases, v_4 dominates the search process by 37% to 53% and as the value of the beta increases, the usage of v_2 increases in parallel, whilst the usage of v_4 decreases. Subsequently v_2 initiates dominating the search process. We consistently observe the expected heterogeneity on the 100-dimensional functions when $\beta = 0.7 - 0.75$, where each velocity update equation is exploited within the tolerated range. We have conducted further experiments using $\beta = 0.7 \text{ and } 0.75$ on different dimensions (10-d, 30-d 50-d) and results exhibited nominal difference in the heterogeneity during the search process across various dimensions hence, a stable heterogeneity is maintained using $\beta = 0.7 \text{ and } 0.75$ in most cases. Hence, $\beta = 0.75$ is used for the main experiments detailed in the paper.

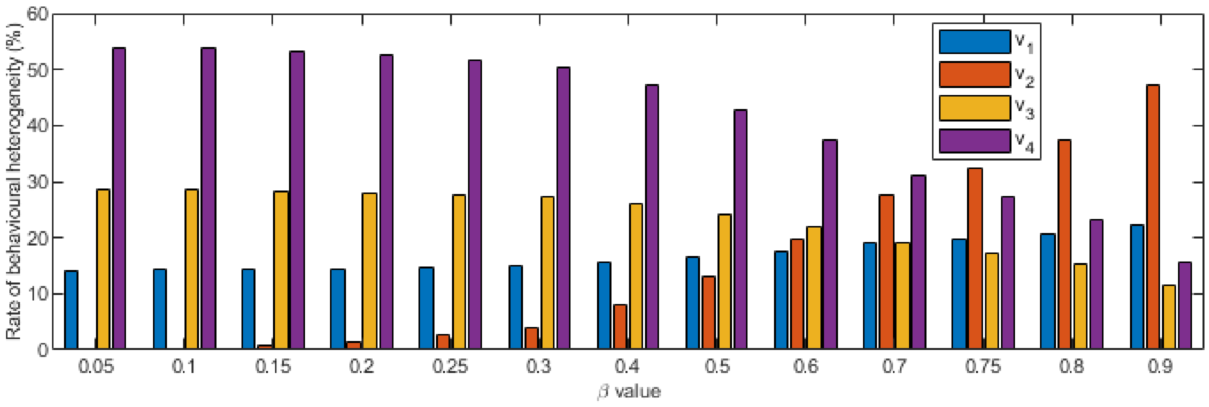

Figure S19-The impact of the β on the behavioural heterogeneity.
